# High-throughput fabrication of nanofibrous yarns produced by alternating-current electrospinning

**DOI:** 10.1038/s44172-026-00737-x

**Published:** 2026-07-22

**Authors:** Jaroslav Mikule, Ondrej Friedrich, Juan Pablo Perez Aguilera, Petr Zabka, Eva Kuzelova Kostakova, Jan Valtera, Sarka Hauzerova, Josef Skrivanek, Vera Jencova, Ondrej Batka, Laszlo Meszaros, Jan Milucky, Manikandan Sivan, Jaroslav Beran, David Lukas

**Affiliations:** 1 Technical University of Liberec, Faculty of Science, Humanities and Education, Department of Chemistry, Liberec, Czech Republic; 2 Technical University of Liberec, Faculty of Mechatronics, Informatics and Interdisciplinary studies, Liberec, Czech Republic; 3 Technical University of Liberec, Faculty of Mechanical Engineering, Department of Textile Machine Design, Liberec, Czech Republic; 4 Budapest University of Technology and Economics, Faculty of Mechanical Engineering, Department of Polymer Engineering, Budapest, Hungary

**Keywords:** Nanoscale materials, Biomaterials, Mechanical engineering

## Abstract

Nanofiber yarns offer unique opportunities for developing next-generation flexible materials that combine nanoscale functionality with macroscopic structural integrity. Their high surface-to-volume ratio, fine open porosity, and fibrous morphology enable applications in tissue engineering, drug delivery, filtration, sensors, energy storage, and functional textiles. However, the transition of nanofiber yarn technology from laboratory to industrial scale has been limited by low productivity and poor control over morphology, linear density, twist, and tensile strength. Here, we report a simple and robust method with strong scale-up potential for the continuous production of 100% nanofiber yarns using high-throughput needleless and collectorless alternating-current (AC) electrospinning. A plume of nanofibers generated from a rotating disc spinning-electrode is continuously deposited on a rotating drum and withdrawn through a spinning triangle, where a twirling device imparts controlled twist. Using this setup, nanofiber yarns composed of poly(vinyl butyral) (PVB), poly(ε-caprolactone) (PCL), polyamide 46 (PA46), and poly(vinyl alcohol) (PVA) were produced at speeds up to 55 m min⁻¹ with a 200 mm disc spinning-electrode. Scanning electron microscopy revealed densely packed nanofibrils within the yarn cross-sections and a dominant pore size around 1 µm. The yarns were braided and woven into scaffolds supporting cell adhesion and proliferation. This high-performance alternating-current electrospinning approach effectively bridges the gap between electrospun fibers—whose two of three characteristic dimensions reside in the nanoscale—and macroscopic textile manufacturing, thereby enabling a practical and scalable pathway for the industrial production of nanofiber yarns for biomedical and advanced material applications.

## Abstract (web summary)

Jaroslav Mikule and colleagues developed a continuous AC-electrospinning method for producing core-free, 100% nanofiber yarns. The method forms yarns from several polymers at up to 55 m min⁻¹ and enables their processing into braided and woven scaffolds that support fibroblast proliferation.

## Introduction

Threads and yarns have served as essential structural elements for more than 50,000 years, providing mechanical robustness and flexibility and representing one of the earliest technological innovations of humankind^1–3^. Conventional staple-fiber yarns behave as friction-dominated assemblies in which numerous inter-fiber contacts generate substantial normal stresses under load, giving rise to high tensile strength^4^. Translating this long-established concept into the nanoscale, nanofiber yarns (NFYs) exhibit unique mechanical and functional properties arising from their high surface-to-volume ratio and nanoscale porosity^4^, while electrospinning enables tunable chemistry^5^ and nanotopography^6^. Advances in electrospinning and in the understanding of nanoscale fiber assembly have markedly expanded NFY fabrication and characterization^7^. Unlike nanofiber mats, whose planar geometry is fixed by the deposition process, NFYs retain high flexibility and formability, decoupling fiber generation from downstream shaping and enabling multiscale material design for textile and biomedical architectures. These characteristics have driven increasing interest in NFYs, including composite and core-free variants, for biomedical applications, where their fibrous architecture resembles the extracellular matrix (ECM) and promotes cell attachment and growth^8–10^. NFYs have therefore emerged as promising scaffolds and drug-delivery platforms; hollow NFYs can provide prolonged therapeutic release^11^, while direct incorporation of bioactive molecules enables multifunctional regenerative systems^12^.

Direct-current (DC) electrospinning remains the most established route for producing nanofibers and has also been widely used for NFY fabrication^13^. Building on the foundational work of Formhals^14–16^, decades of innovations—including conjugated electrostatic spinning and multi-nozzle systems—have improved yarn uniformity and mechanical stability^17–22^.

An important advance was the introduction of collectorless alternating-current (AC) electrospinning by Lukáš and co-workers^23^, later reviewed by Stanishevsky^24^. AC electrospinning exploits alternating jet polarity to generate periodic self-neutralization of fibers, forming a transient *virtual collector* several centimetres from the electrode^23^. The dynamics of this charge recombination zone—governed by field frequency and waveform—strongly affect fiber trajectories and plume morphology^25^ and, when combined with needleless electrodes, enable substantially higher nanofiber production rates than comparable DC electrospinning approaches^26^. This principle has also been applied to the fabrication of composite NFYs by wrapping an AC-generated nanofibrous plume around a supporting core thread^27^, thereby combining nanoscale functionality with macroscale mechanical integrity^28–30^, but still relying on the core to provide continuous yarn formation.

Despite the advances, a major unresolved challenge remains: the continuous high-throughput fabrication of mechanically processable, core-free nanofiber yarns composed entirely of electrospun nanofibers. Previously reported DC-based NFY fabrication strategies are constrained by relatively low solution feed rates and modest collection speeds, with representative yarn take-up speeds often below 2 m min⁻¹ and reaching about 10 m min⁻¹ only in post-drawing-assisted configurations^31,32^. These limitations arise in part from the electrostatic nature of DC electrospinning, where emitted polymer jets and deposited fibers typically carry charges of the same polarity and undergo Coulombic repulsion, promoting jet divergence and spatial dispersion of the forming fibers. This makes it difficult to generate a compact, mechanically coherent nanofibrous plume suitable for direct transformation into continuous linear structures. AC electrospinning provides a route to overcome these limitations through periodic charge reversal and self-neutralization, producing a compact, electrically neutral nanofibrous plume and enabling substantially higher nanofiber production rates. However, previously reported AC-electrospinning-based NFYs have remained core–shell composite structures, in which an AC-generated nanofibrous sheath is formed around a supporting core thread. Although this core provides the principal macroscale continuity and mechanical integrity, its presence may limit applications requiring fully nanofibrous, core-free yarn architectures^31^.

To address this gap, we developed a needleless, collectorless AC electrospinning strategy that directly transforms an electrically neutral nanofibrous plume into continuous 100% nanofiber yarns through a controlled spinning triangle and false-twist/twirling mechanism. This approach eliminates the need for a supporting core, enables control over yarn morphology, linear density and twist-related structure, and achieves production speeds approaching two orders of magnitude above those reported for representative conventional DC NFY-forming methods. In the Results section, we describe the spinning setup, analyse spinning-triangle dynamics, characterize yarn morphology and tensile behavior, demonstrate processability into braided and woven structures, and evaluate cell proliferation on fully nanofibrous scaffolds. Together, these findings establish AC electrospinning as a scalable route for producing nanofiber yarns suitable for advanced technical and biomedical applications.

## Results

**Fig. 1:**
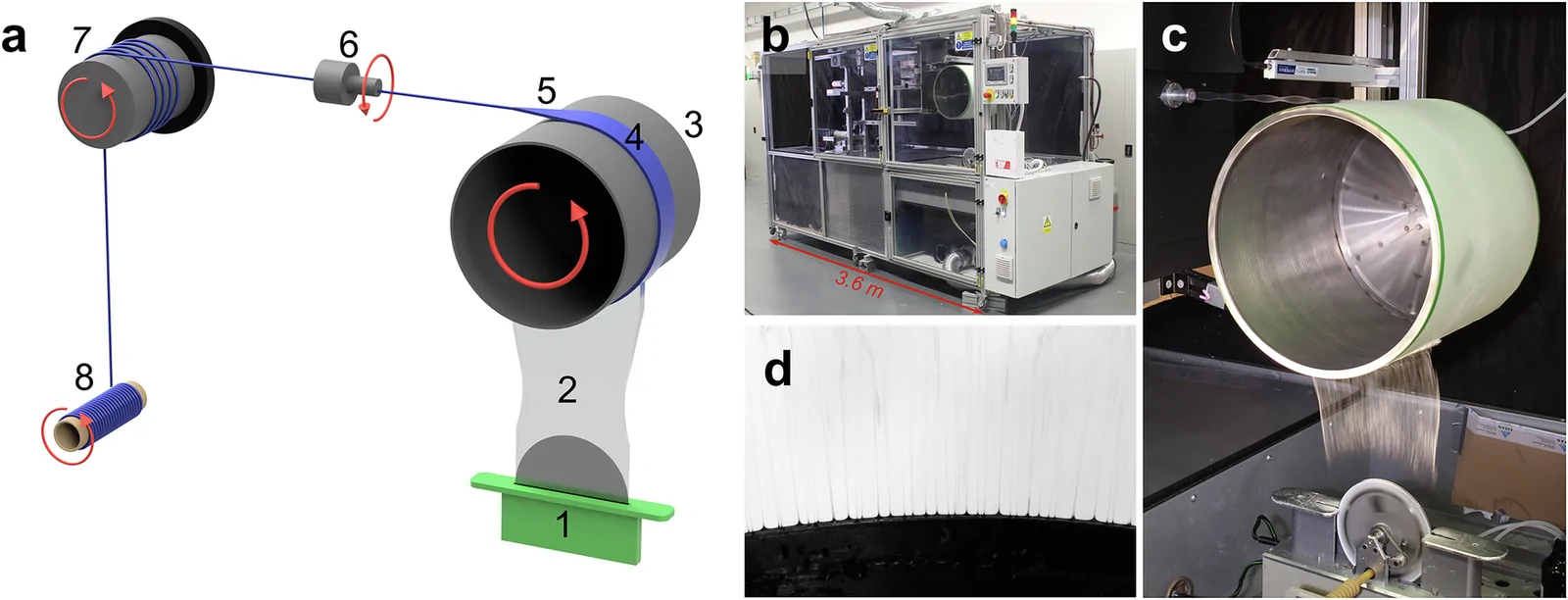
Overview of the experimental setup and nanofiber yarn production process. **a** Schematic representation of the system: (1) rotating disc spinning-electrode with an integrated polymer reservoir, (2) plume of electrospun nanofibers, (3) mechanical collector in the form of a rotating drum, (4) belt of deposited nanofibers on the drum collector, (5) spinning triangle, (6) twirling device, (7) galette, and (8) bobbin with the wound nanofiber yarn. **b** Photograph of the production line for continuous fabrication of 100% nanofibrous yarns. **c** Disc-type AC spinning electrode generating a stable curtain-shaped nanofiber plume during operation. **d** Formation of multiple equidistant polymer jets along the disc circumference due to electrohydrodynamic instabilities.

We developed a technique for the continuous production of nanofiber yarns, schematically illustrated and photographically documented in Fig. 1a–c. The approach integrates several patented innovations that extend our previous work on high-throughput electrospinning technologies^23,27,33–35^. Firstly, in this method, nanofibers are generated using a collectorless and needleless alternating-current (AC) electrospinning system equipped with a rotating disc electrode. A distinctive feature of this configuration is the formation of a nanofibrous plume, resembling a thin, veil-like curtain of interconnected fibers (Fig. 1c). Driven by an electric wind, the plume propagates upward approximately along the plane of the rotating disc spinning-electrode^23^. The spinning-electrode and polymer-solution reservoir are integrated into a single compact unit, an integrated spinning-electrode, that ensures stable and continuous operation. During rotation, the polymer solution is drawn from the reservoir to the disc edge, where multiple, evenly spaced polymeric jets emerge due to electrohydrodynamic instabilities on the liquid surface^36^. The excess polymer solution not utilized in fiber formation is recirculated back into the reservoir through the electrode’s rotation. This self-cleaning and self-feeding mechanism maintains a steady-state fiber formation regime in which the disc edge is continuously refreshed by new polymer solution while residual solid nanofiber fragments are simultaneously removed. Supplementary Movie 1 illustrates the process dynamics in real time.

Secondly, the electrically neutral plume of nanofibers generated by AC electrospinning is collected as a narrow, fluffy belt on an electrically inactive rotating drum. This clearly distinguishes the process from DC electrospinning, which typically produces compact, electrostatically flattened, and dense nanofiber layers. The cylindrical surface of the custom-built drum collector was covered with parchment paper (40 g·m⁻²), to facilitate uniform fiber deposition an first of all optimal adhesion for the nanofiber web (see Supplementary Movie 2). The narrow width of the nanofiber belt, which forms the base of the spinning triangle, is determined by the perpendicular alignment of the spinning-electrode and collector drum axes, together with the extraordinary narrowness and coherence of the nanofiber plume generated by the disc-electrode. The plume resembles a thin, smoke-like curtain/veil with an exceptionally small cross-sectional thickness—an attribute difficult to achieve with DC electrospinning. In DC systems, the electrospinning jets and deposited fibers diverge due to electrostatic repulsion between fibers carrying charges of the same polarity, whereas in AC electrospinning the periodic reversal of polarity effectively suppresses this effect, resulting in a compact and cohesive nanofiber flowing plume.

The substrate covering the surface of the collector drum provides sufficient mechanical adhesion of the nanofiber layer to the rotating surface. The strength of this adhesion is crucial for the formation and stabilization of the base of the spinning triangle. By adjusting the circumferential speed of the collector drum, the areal mass of the deposited nanofiber belt can be precisely controlled, thereby determining the fineness and uniformity of the resulting nanofiber yarn.

Thirdly, a key step in nanofiber yarn formation is the twisting of the nanofiber belt into a continuous yarn. During this process, a tensile force is required to detach the nanofiber belt from the drum surface, while a torque must be applied to impart twist and consolidate the fibers into a coherent yarn structure. However, the fluffy nanofibrous material accumulated on the rotating drum adheres strongly to any solid surface, which prevents direct mechanical engagement. As a result, torque is transmitted through the nanofibrous belt itself, without direct mechanical contact with any rigid tool. This transmission arises from the collective mechanical response of the densely entangled nanofiber network, in which inter-fiber friction, van der Waals interactions, and geometric confinement within the spinning triangle—initially characterized by only a negligible shear moment—enable the propagation of shear stresses across the belt. The path extending along the spinning triangle, through the newly formed nanofibrous yarn and toward its anchoring in the twirling device, may be viewed as an axis of progressively increasing shear modulus, as the nanofibrous material becomes increasingly compact, denser, and richer in frictional and van der Waals contacts. The efficiency of this mechanism depends on the fiber density and belt thickness.

**Fig. 2:**
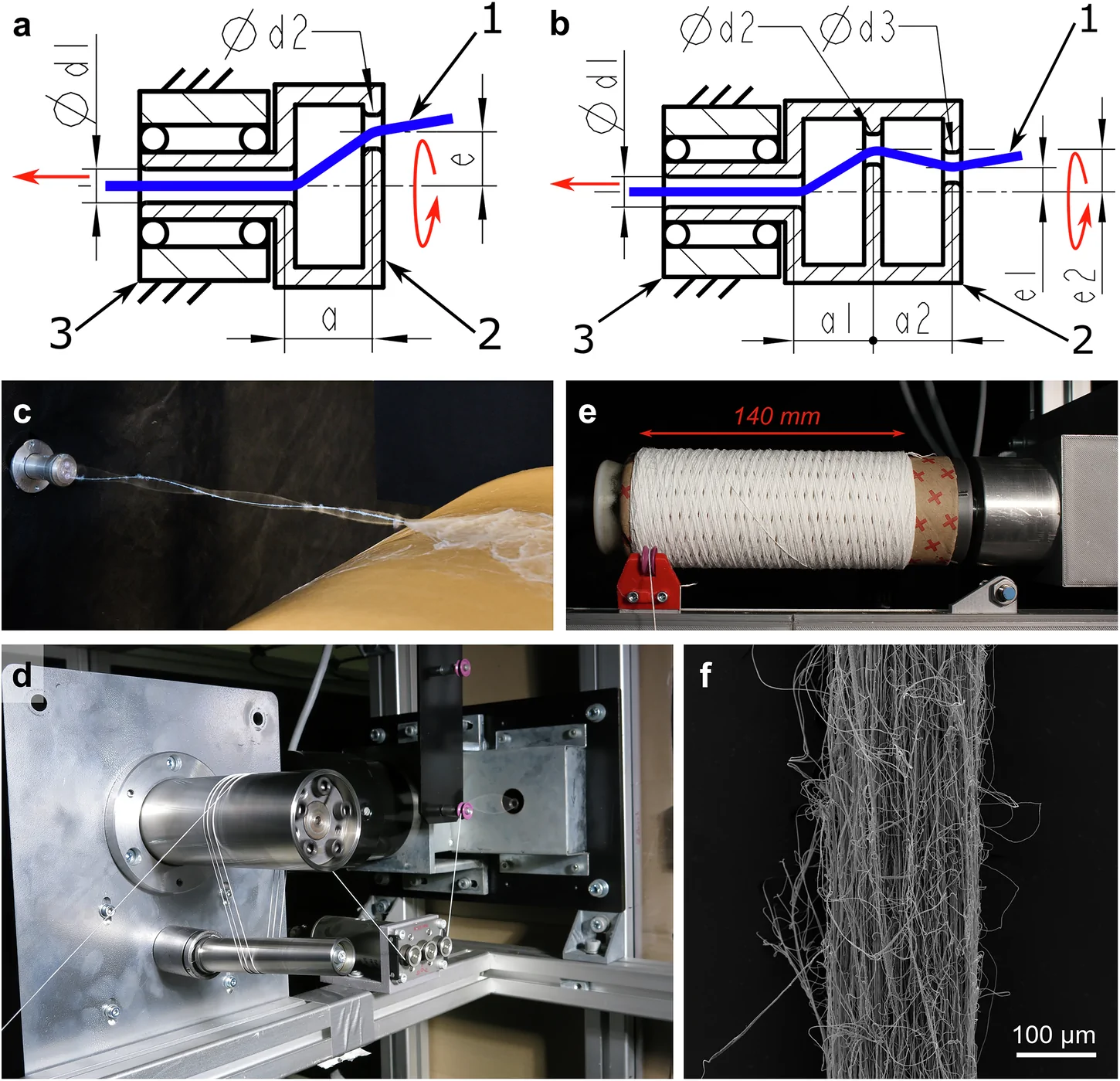
Twirling device and formation of nanofibrous yarn. **a**, **b** Schematics of single-stage and two-stage twirling devices in which the twisted nanofiber bundle moves from right to left (red arrow). **a** Single-stage design equipped with one ceramic guide eyelet. **b** Two-stage design with two ceramic guide eyelets providing enhanced yarn stability. The eyelet eccentricities are denoted e₁, e₂; the distances from the outlet tube are a, a₁, a₂; and the eyelet diameters are d₁–d₃. **c** Section of the spinning triangle showing the twirling device on the left and the ballooning yarn in between. **d** Galette used for yarn guiding and tension control. **e** Spool with continuously wound 100% poly(vinyl butyral) (PVB) nanofibrous yarn. **f** Scanning electron micrograph of a poly(vinyl butyral) (PVB) nanofibrous yarn produced at 55 m min⁻¹ revealing pronounced alignment of individual fibers along the yarn axis.

In our system, the tensile force required for peeling the nanofibrous belt from the drum surface is not applied independently, but arises as a direct consequence of the torque transmitted through the nanofibrous structure. This coupling is primarily determined by the rotational speed of the twirling device, and the geometry of the setup, particularly by the relative position of the drum collector, its surface, and the twirling device, which together define the shape of the spinning triangle and the direction of force transmission. In this sense, the coupling is intrinsic to the selected system geometry. A qualitative experimental indication of effective peeling is provided by the state of the drum surface after separation of the nanofibrous belt. In long-term spinning experiments, we do not observe progressive accumulation of residual nanofibrous material on the drum. The initiation of nanofiber yarn spinning is achieved by allowing the nanofibrous layer on the drum collector to adhere to the end of a guide thread, which is threaded through a twirling device. The twirling device essentially consists of a rotating disc with an eccentric hole (see Fig. 2a–c), which imparts torque to the guide thread through friction. As the guide thread continously rotates at the entrance to the twirling device, it twists the leading edge of the nanofiber belt that has adhered to it on the drum surface. The twisted nanofibrous mass, drawn into the twirling device by the guide thread, becomes capable of transmitting torque—and therefore twist—to the nanofiber spinning triangle even after the guide thread is cut. This mechanism ensures stable and continuous production of nanofiber yarns.

The twirling device induces a *false twist*—a transient local twist generated by yarn friction within the rotating eyelet, which disappears upon release. In an ideal process, the fiber bundle acquires an equal number of opposite “S” and “Z” twists before and after the twirling device, resulting in zero net twist. When this balance is disturbed, a portion of the twist remains, producing a residual twist with local “S” or “Z” character while the average yarn twist remains zero. Variations in parameters such as the wrapping angle, yarn diameter, or frictional conditions can contribute to this effect, ultimately influencing the final yarn structure and mechanical properties. Unlike false twisting of conventional fibers, false twisting in our nanofiber-yarn formation process imposes lateral compressive stresses that yield a high–fiber-volume-density yarn through permanent bundle compaction, which is effectively fixed by van der Waals–mediated inter-fiber cohesion. Equally important, the mechanically plastic nanofibrous mass entering the twirling device undergoes continuous unidirectional rotation, enabling the formation of a stable spinning triangle that supports continuous yarn production.

**Fig. 3:**
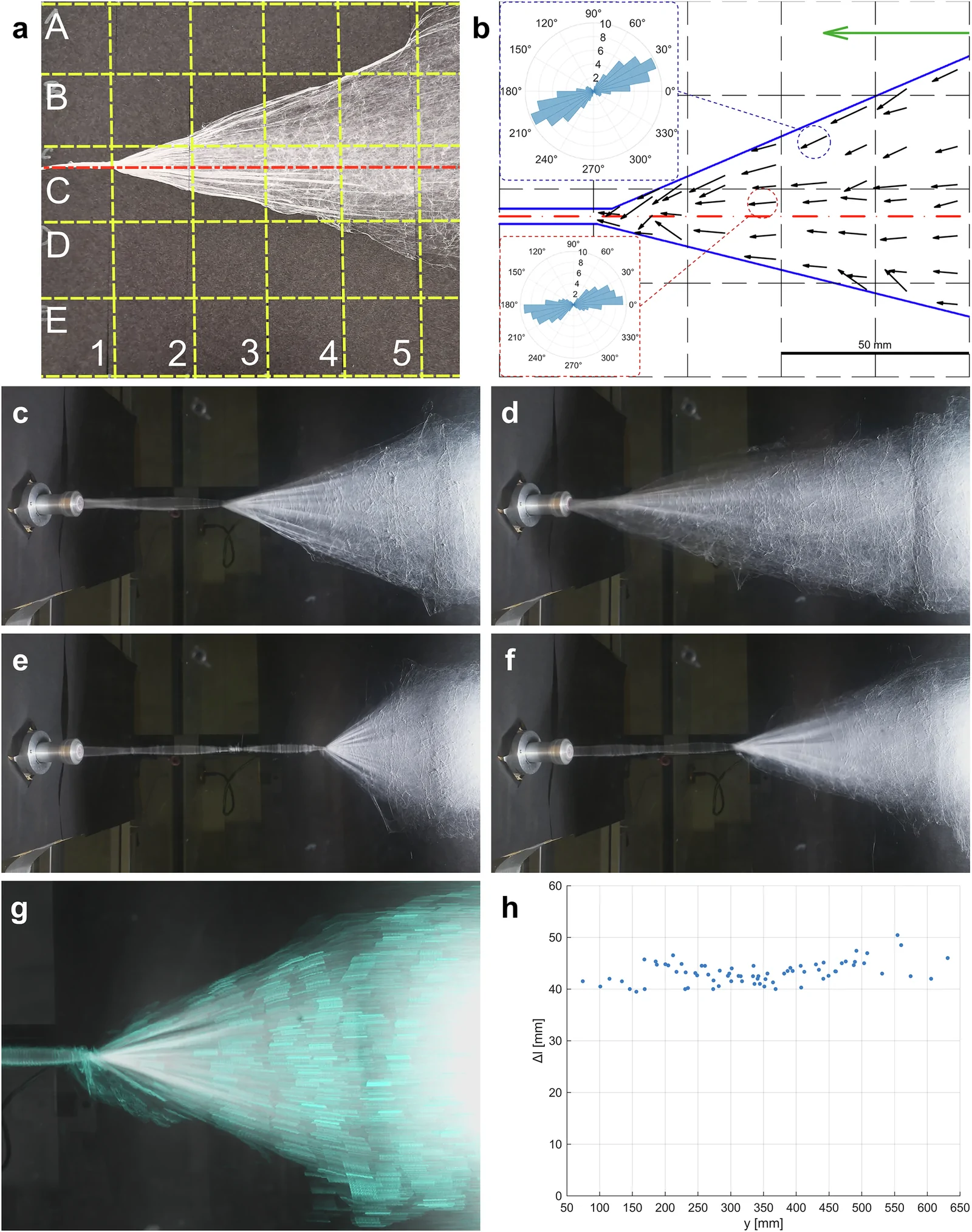
Characterization of the spinning triangle and fiber orientation. **a** Captured image of the spinning triangle divided into 25 × 25 mm square regions for orientation analysis. **b** Corresponding principal rose vector field showing the predominant nanofiber orientation frequencies at selected positions within the triangle. The green arrow indicates perfect fiber alignment along the yarn production axis (0°/180°), while the red dashed line marks the central yarn axis. **c**, **d** Shortest and longest spinning triangles observed at a twirling device speed of 8000 rpm. **e**, **f** Shortest and longest spinning triangles observed at 16 000 rpm, recorded over a 5 min interval demonstrating process stability. **g** Visualization of nanofiber motion within the spinning triangle at 16 000 rpm, captured over 1 s. **h** Plot of fiber displacement (Δl) versus distance (y) showing no significant drawing effect, as all tracked protrusions follow comparable path lengths per second, independent of their location along the y-axis perpendicular to the spinning-triangle direction.

As described above, the nanofiber belt is continuously withdrawn from the rotating drum collector and twisted within the so-called spinning triangle, a critical zone fundamental to yarn spinning across all fiber systems. (see Fig. 2c, Fig. 3 and Supplementary Movie 3). The geometric and morphological characteristics of the spinning triangle are of fundamental importance for optimizing nanofiber yarn quality. They govern how the nanofibers are twisted into the final yarn structure—determining parameters such as curvature, torsion, and mutual fiber arrangement (e.g., degree of anisotropy)^37–39^. The nanofiber arrangement within the spinning triangle strongly influences the resulting yarn morphology and key performance parameters, including tensile strength, hairiness, and linear density uniformity.

In conventional ring-spinning systems, a flat ribbon of fibers—analogous to the nanofiber belt collected on our drum—passes through the front roller nip, where twist is imparted to the fibers by the rotating traveler or by a false-twist device^40^. In such systems, the spinning triangle has a well-defined width and a base fixed by the roller nip. In our setup, the rotating drum collector provides an analogous function through frictional and adhesive interactions with the nanofibres, entirely in the absence of mechanical nipping. These interactions furnish a stable base for the nanofiber belt, maintaining a geometrically controlled and dynamically stable spinning triangle during continuous yarn formation.

To elucidate the stability of the spinning triangle in our configuration, we propose that its base is anchored to the drum collector substrate through a combination of mechanical interlocking, van der Waals interactions, and electrostatic attraction acting in concert. At present, these contributions cannot be directly resolved experimentally and are therefore formulated as physically motivated hypotheses. We assume that the mechanical component of adhesion originates from the geometric interlocking of nanofiber segments with the surface of the substrate. The prominence of van der Waals interactions stems from the exceptionally large specific surface area of the nanofibers, whereas electrostatic attraction is attributed to triboelectric charging occurring during the detachment of the nanofibrous layer from the substrate. The electrostatic component can be effectively controlled using an ionization bar (Meech 924/PSv2, 320 mm in length, ±7.5 kV) positioned above the base of the spinning triangle. The frictional conditions at the triangle base must be optimized by appropriate selection of the substrate surface. Excessive adhesion prevents the nanofiber belt from being detached by the advancing, freshly twisted yarn, whereas insufficient adhesion leads to the collapse of the spinning triangle and uncontrolled rolling of the nanofiber belt across the drum surface. Backing paper provides the required balance for all polymers evaluated in this study.

The freshly formed nanofibrous yarn rotates at high speed between the twirling device and the tip of the spinning triangle, giving rise to the formation of a so-called yarn balloon. This behavior occurs because the yarn behaves as a pre-stressed, linear, and flexible structure^41^ (see Fig. 2c). The stability of both the yarn balloon and the spinning triangle is substantially enhanced by a rotating galette (Technical University of Liberec) positioned approximately 200 mm downstream of the twirling device, where it securely grips the forming yarn (Supplementary Movie 4). The galette defines a downstream boundary condition for the twisting process, acting as a tension stabilizer and twist brake. Without this stabilization, twist propagation toward the spinning triangle leads to dynamic instability. A mechanical tension sensor placed in front of the galette continuously monitors the yarn tension (Fig. 2d).

Twist accumulation in the region behind the twirling device can generate a torque large enough to overcome the frictional resistance within the twirling eyelet, transferring an opposite twist toward the tip of the spinning triangle and thereby inducing dynamic instability. The rotating galette effectively suppresses this effect by acting as a twist brake while maintaining a constant yarn take-up speed. Together with the base of the spinning triangle, it defines two effective clamping points between which the nanofiber yarn receives the false twist, thereby contributing to the stabilization and uniformity of the twisting process.

Lastly, the resulting nanofibrous yarn is continuously wound onto the take-up spool. The above-described process enables the efficient transfer of nearly 100% of the nanofibers deposited on the substrate covering the drum collector into the formed yarn. This method of nanofiber yarn production is highly robust with respect to the type of polymer used and the efficiency of fiber spinning, as demonstrated here for poly(vinyl butyral) (PVB), polyamide 46 (PA46), poly(ε-caprolactone) (PCL), and poly(vinyl alcohol) (PVA) (see Table 1).

**Table 1:** Process parameters and resulting production rates of nanofibrous yarns fabricated from different polymers

Process parameter			Value	
Material	PA46	PCL	PVA	PVB
Ambient temperature [°C]	19	27	27	24
Relative humidity [%]	60	40	30	40
VTS 38 high-voltage transformer input/output V_PP_^a^ [V/kV]	250/47	250/47	250/47	250/47
Frequency [Hz]	100	50	50	50
Drum rotation speed [m/min]	0.8	4	2	7.1
Twirling device rotation speed [rpm]	6000	10000	9000	16 000
Eccentricity of eyelets [mm]	5; 6,2	5; 6,2	5; 6,2	5; 6,2
Winding speed [m/min]	0.85	6	2.2	9.4
Production speed [m/min]	0.85	6	2.2	9.4
Solid mass productivity [g/h]	1.6	13.1	4.1	18.2
Polymer solution consumption rate [g/h]	11.1	119.1	41.1	140.0
Linear density / Fines [tex]	30.4	36.4	31.11	32.28

The table summarizes key processing conditions for AC electrospinning of polyamide 46 (PA46), poly(ε-caprolactone) (PCL), poly(vinyl alcohol) (PVA), and poly(vinyl butyral) (PVB). The data highlight the robustness and versatility of the AC electrospinning process across a wide range of polymer chemistries.

^a^V_PP_ is the peak-to-peak voltage between the most positive and most negative peaks of a waveform.

### Productivity

The production performance of nanofibrous yarns prepared from PVB, PA46, PCL, and PVA polymers is summarized in Table 1 and Table 2. The listed parameters include the linear and mass production rates, as well as the polymer solution consumption rate, which directly reflects the conversion efficiency into solid nanofibrous material. A key metric for scaling this technology to an industrial level is the production speed, which ranged from 0.85 to 9.4 m min⁻¹, accompanied by relatively high linear densities of 30.4–36.4 tex. These processing conditions were selected to obtain yarns within the targeted fineness range of 30–40 tex and correspond to routine stable operating conditions for continuous yarn formation across all investigated materials. However, the process allows for straightforward adjustment of the linear density across a broader range; in particular, coarser yarns up to ~100 tex can be readily produced by reducing the winding speed, along with appropriate adjustments of the drum rotation speed and the twirling device rotation speed, while maintaining the desired yarn quality. A remarkable feature of our process is its operational stability, enabling continuous yarn formation for at least one hour without interruption, as evidenced by a 300 m long wound spool (Fig. 2e). This stability was confirmed for each material by repeated experiments (typically 10–50 runs), consistently yielding yarns of comparable quality and resulting in the cumulative production of several hundreds of meters to kilometers of yarn. In general, higher linear densities were associated with increased process stability, as the larger amount of fibrous material in the yarn reduces the likelihood that local inhomogeneities arising during the spinning process lead to yarn breakage, whereas at fineness below of 20 tex the stability gradually decreases. Furthermore, a dedicated experiment with PVB nanofibrous yarn demonstrated a maximum achievable production speed of 55 m min⁻¹, corresponding to a yarn fineness of 5 tex (Fig. 2f). Beyond this threshold, the process became unstable, and yarn breakages were observed in the region of the twirling device.

**Table 2:** Operational parameters of the AC electrospinning setup

Parameter	Value
Diameter of disc spinning electrode [mm]	200
Thickness of disc spinning electrode [mm]	1
Disc rotation speed [rpm]	30
Distance between spinning electrode and drum collector [mm]	230
Diameter of drum collector [mm]	430
Gear pump speed (solution dosing) [rpm]	60
Gear pump speed (solution return) [rpm]	100
Magnetic stirrer speed [rpm]	200

### Analysis of the spinning triangle

**Fig. 4:**
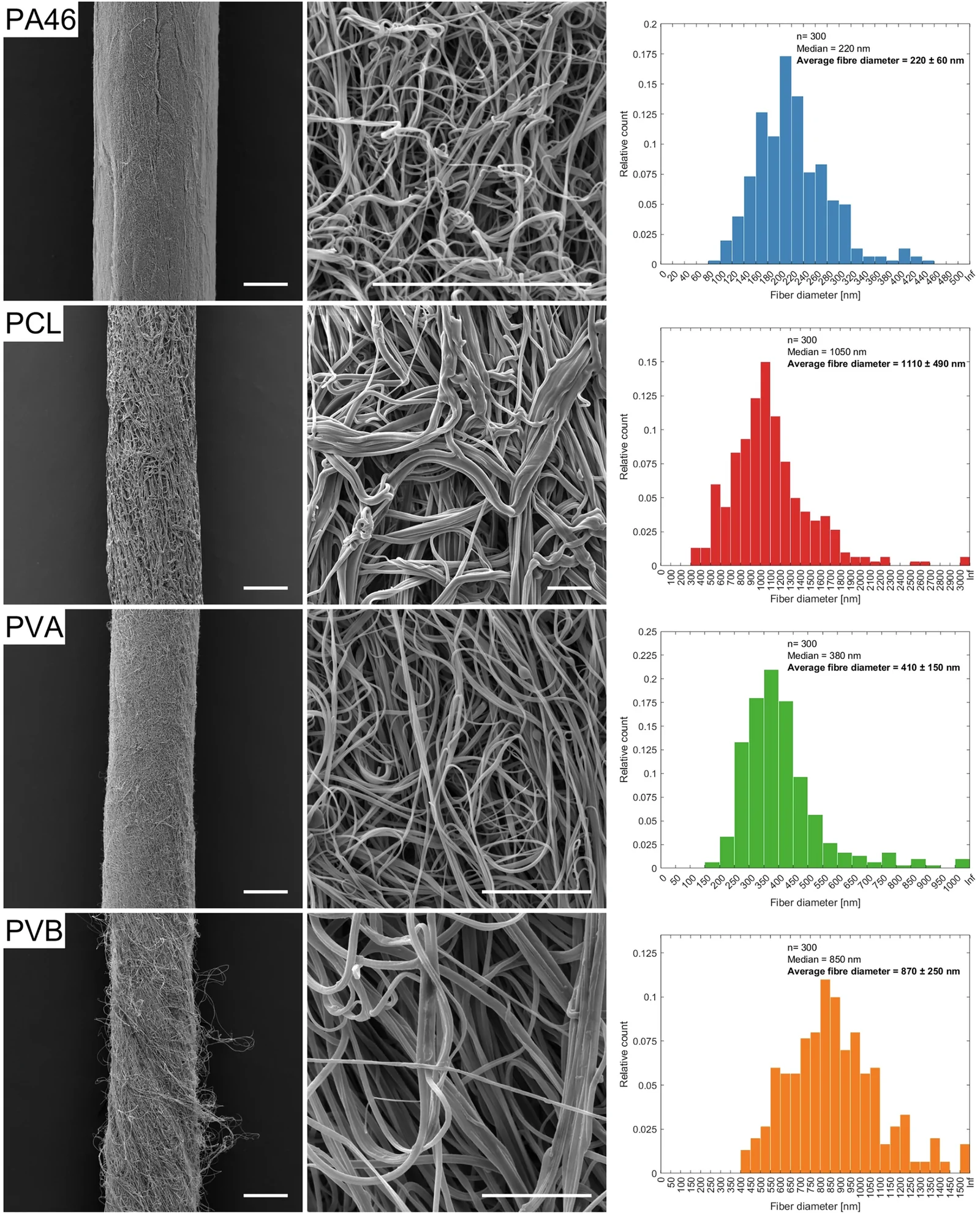
Morphology and fiber diameter distributions of nanofibrous yarns prepared from different polymers. Left: SEM images of representative yarn segments made from PA46, PCL, PVA, and PVB (scale bar: 200 µm). Center: High-magnification SEM micrographs showing the nanofibrous architecture and interfiber connections within each yarn (scale bar: 20 µm). Right: Corresponding fiber diameter distribution histograms, illustrating the characteristic fiber diameter ranges and mean fiber sizes for each polymer system.

To investigate the fiber orientation within the spinning triangle, a sample was collected by halting the PA46 nanofibrous yarn production and carefully transferring the intact spinning triangle onto a sheet of paper without disturbing the fiber arrangement (Fig. 4a). The paper with adhered nanofibers was subsequently sectioned into square samples (25 × 25 mm) to enable precise localization and analysis of in-plane anisotropy based on scanning electron microscopy (SEM) images.

The result of this analysis is a so-called roses of directions describing the frequency of orientation of nanofiber segments in individual directions^42^. The roses of directions were then used to create a vector field graphically representing the spatial distribution of the prevailing nanofiber orientation. Orientations of these vectors, that we will call here principal rose vectors, are determined by the angles of the roses of directions with the highest frequency values, and magnitudes of the vectors are determined by the values of maximum frequencies, see Fig. 3b. The 180^o^ direction corresponds to the direction of yarn production.

The analysis of planar anisotropy revealed a pronounced local fiber alignment directed toward the apex of the spinning triangle. Fibers located near the triangle sides exhibited lower local variance in orientation compared with those near the triangle axis, as indicated by the longer principal rose vectors (Fig. 3b). More substantial fiber rearrangement was observed in regions further from the axis, which can be attributed to the “S-type” twist generated during yarn formation. In this configuration, the upper side of the spinning triangle is preferentially detached from the collector drum, whereas the lower side remains stabilized by its contact with the drum surface (Fig. 3a). The intersection of principal rose vectors at the apex region, corresponding to the yarn formation zone, confirms the presence of an “S” twist, determined by the rotation direction of the twirling device (for more details see Supplementary).

The kinematics and stability of the spinning triangle were examined through five-minute video recordings. The observed instability manifests as a fluctuating triangle height, which varies with the rotational speed (rpm) of the twirling device (Fig. 3c–f). With increasing rotational speed, the height of the spinning triangle systematically decreases, reflecting the higher number of twists transmitted per unit time to the nanofiber bundle being detached from the collector drum.

We employed optical method for tracking the trajectories of nanofibrous strands per unit time. The approach relies on analyzing local changes in pixel brightness within 30-fps video sequences. Pixels exhibiting large local brightness variations—arising where protruding fibers cast shadows under oblique illumination—are highlighted in cyan. This visualization delineates the paths of individual fiber protrusions over time. A representative image obtained during a 1 s observation period is shown in Fig. 3g, where the visualized displacements of individual protrusions within the spinning triangle converge toward its apex, as expected from the yarn formation dynamics. Interestingly, the magnitudes of these displacements are nearly identical across the observed field, indicating no significant relative elongation or drawing effect of the nanofibrous material within the spinning triangle. This observation is consistent with the data presented in Fig. 3h, showing uniform path lengths of tracked protrusions.

### Fiber diameters

Scanning electron microscopy confirmed the fibrous structure of yarns prepared from all four investigated polymers. The yarns made from PA46, PVA, and PVB consisted predominantly of nanofibers with average diameters of (220 ± 60 nm), (410 ± 150 nm), and (870 ± 250 nm), respectively, whereas the biodegradable PCL yarn was composed mainly of micron-scale fibers with a broad diameter distribution of (1140 ± 490 nm) (Fig. 4). For all materials, a varicose fiber morphology and interfiber bonding were observed, which are typical features of alternating current (AC) electrospinning^23^. In the case of PVA, occasional bead-like defects were detected, while for the other polymers such defects were either very rare or absent. Scanning electron microscopy analysis further revealed a non-uniform twist distribution along the yarn axis for all nanofibrous yarn types (see Supplementary). This phenomenon may result from partial slippage of residual twist within the twirling device during yarn formation.

### Post-treatment and surface modification

**Fig. 5:**
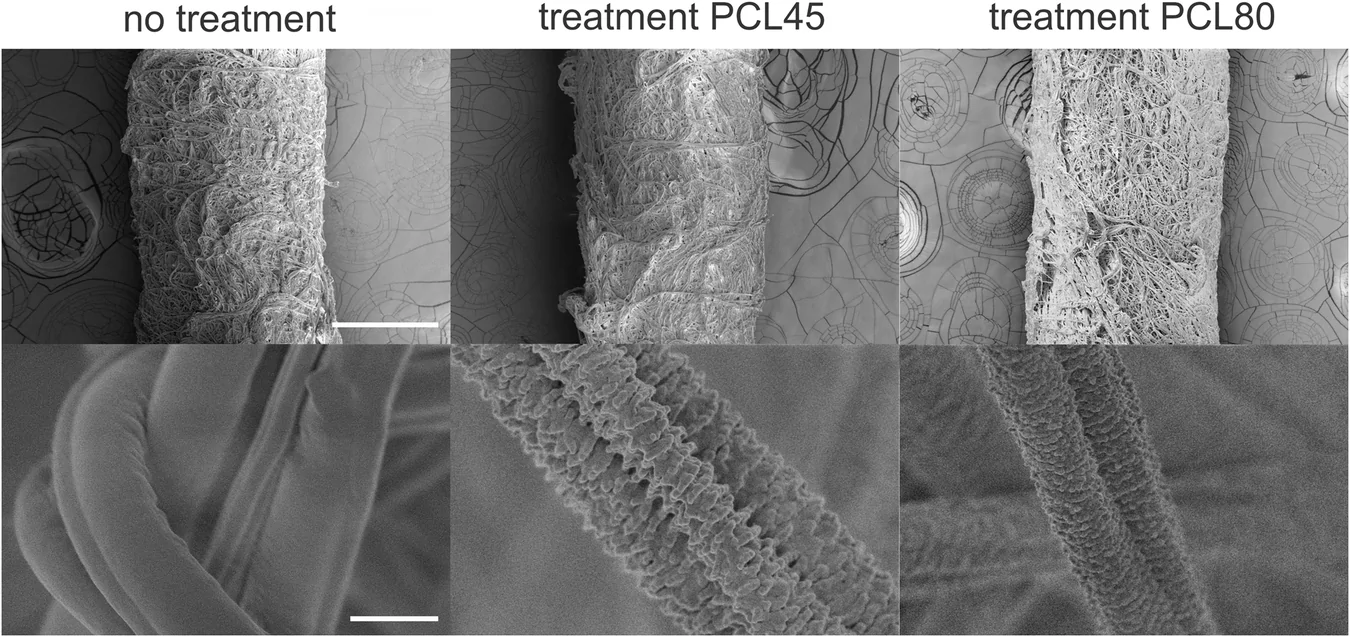
Surface modification of PCL nanofibrous yarns via shish–kebab formation. Top row: SEM micrographs showing the overall morphology of PCL nanofiber yarns before and after post-treatment by spraying diluted PCL solutions of different molecular weights (45 kDa and 80 kDa) (scale bar: 300 µm). The untreated yarn exhibits a relatively smooth surface, whereas treated samples display progressively increased surface roughness. Bottom row: High-magnification SEM images (scale bar: 1 µm) reveal the development of periodic shish–kebab nanostructures—polymer crystalline protrusions growing perpendicularly from the fiber backbone—which substantially increase the specific surface area of the fibers and provide a hierarchical nano–micro texture relevant for biomedical and antimicrobial surface design.

The surface morphology of PCL nanofiber layers can be tailored to form shish–kebab structures, consisting of a cylindrical backbone decorated with a periodic array of polymer crystals several hundred nanometers in size^43^. Here, we demonstrate that nanofibrous yarns can be post-processed in an analogous manner. The shish–kebab modification was induced on initially smooth fibers within PCL nanofiber yarns by spraying a diluted solution of the same polymer (Fig. 5). Specifically, the yarns were treated with a 1 wt% PCL solution prepared in pentyl acetate. The resulting nanoridge morphology markedly increased the effective fiber surface area, which is expected to enhance eukaryotic cell adhesion, making the material promising for biomedical scaffolding applications. Conversely, such nanoscale topographies may also mechanically disrupt microbial extracellular polymeric substances (EPS), mimicking the bactericidal nanostructures naturally occurring on cicada and dragonfly wings^44,45^.

### Fiber density and pore size distribution

The pore size distribution in nanofibrous yarns is a critical parameter influencing not only their biomedical performance, but also their filtration efficiency, and potential applications in sportswear and protective textiles. The analysis follows the definition of pores introduced by Masounave^46^. Consider a two-dimensional cross-section of a nanofibrous structure, in which individual fibers appear as circular or elliptical shapes, separated by voids. For analytical simplicity, the fiber cross-sections can be represented by points corresponding to their centroids. In this model, a pore is defined as a circle whose center lies anywhere within the reference area, and whose radius *r* equals the distance from the pore center to the nearest fiber centroid. The probability density function *f(r)* of the pore radii is then expressed as

1 $$f\left(r\right)=2\pi {N}_{S}r{e}^{-\pi {N}_{S}{r}^{2}}.$$

where *N*_S_ is the number of fibers per unit area of the cross-section. The most probable pore radius *r*_p_ corresponds to the maximum of the probability density function *f*(*r*). It can be expressed as

2 $${r}_{p}=\frac{1}{\sqrt{2\pi {N}_{S}}}\cdot$$

**Fig. 6:**
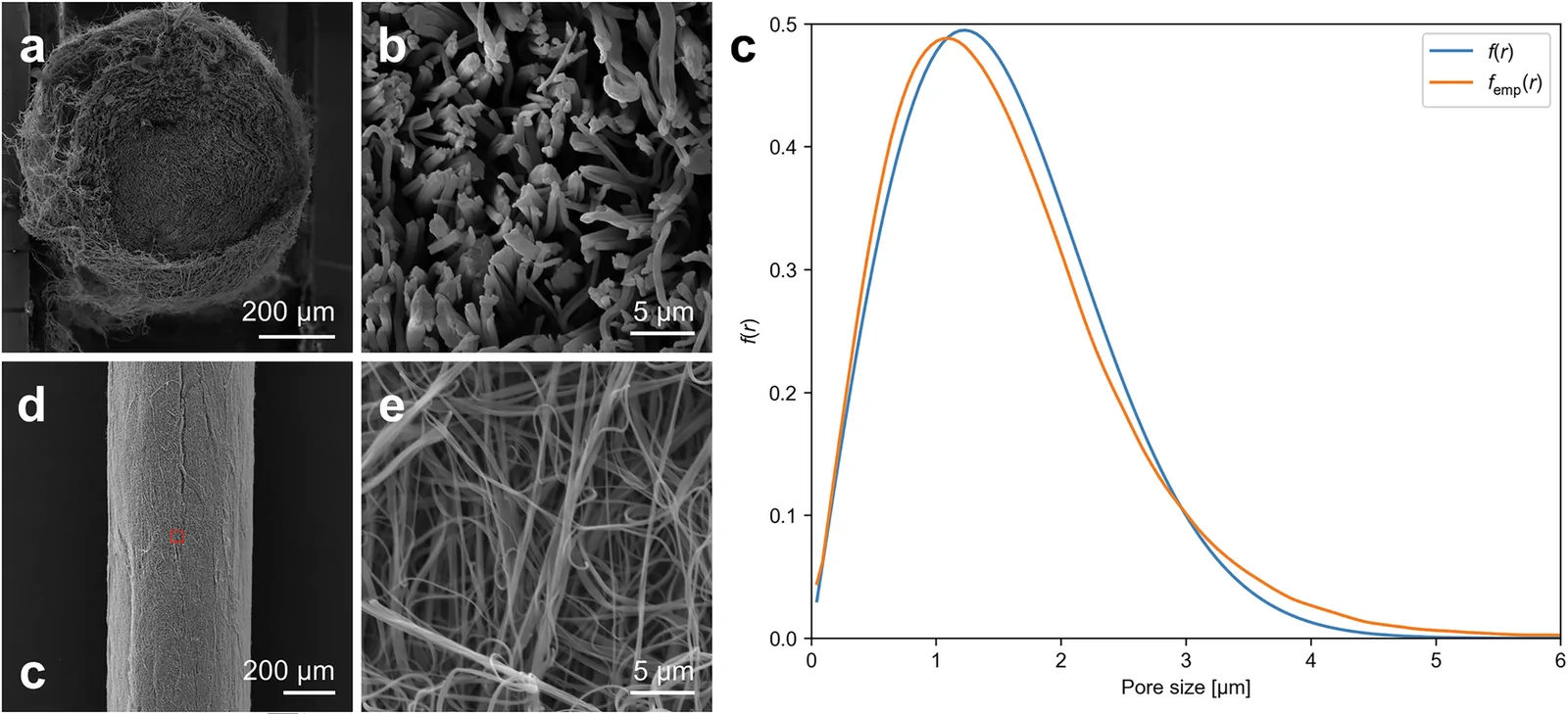
Structural characterization of nanofibrous yarn cross-sections and pore geometry. **a** SEM cross-sectional image of a PVB nanofibrous yarn, showing its densely packed internal structure. **b** High-magnification SEM view of the fiber cross-sections within the same yarn, revealing the compact arrangement and interfiber voids. **c** Probability density function of the pore radius, calculated from the measured fiber number density N_S_ = 0.106 μm⁻². The blue curve shows the Poisson-based analytical prediction averaged over multiple images, whereas the orange curve represents the corresponding Monte Carlo average obtained from the real data. **d** SEM image of a PA46 nanofibrous yarn analyzed for fiber average curvature and twist; the red square marks the position of the analyzed region within the yarn. **e** Representative SEM image of the marked region used for the fiber curvature and twist analysis, showing the local orientation and entanglement of nanofibers.

A perpendicular cross-section of a PVB nanofibrous yarn obtained using a Tescan Vega SEM is shown in Fig. 6a,b. In total, *n* = 1270 fiber endpoints, including merged structures typical of AC-type nanofibrous systems, were identified within a cumulative analyzed area of 11 995 µm², across four images, corresponding to a fiber number density *N*_S_ = 0.106 μm^−2^. Based on this value, the probability density function *f*(*r*) of the pore radius was calculated (Fig. 6c), yielding a most probable pore radius of *r*_p_ = 1.25 μm. However, analysis of the extracted point processes revealed deviations from an ideal Poisson distribution; therefore, complementary Monte Carlo simulations based on the real data were performed, yielding a most probable pore radius of *r*_p_ = 1.13 μm. A detailed stereological analysis of the point processes for individual images is provided in the Supplementary. It should be noted that the nanofibrous yarn exhibits pronounced anisotropy, as demonstrated by the analysis of the spinning triangle. Consequently, the applied pore model does not account for anisotropic effects that may arise in other orientations of the fibrous structure. A rigorous stereological description of such an anisotropic system would require isotropic sections; however, these are not experimentally accessible in the present case. Non-perpendicular sections tend to disintegrate without reinforcement, while resin embedding introduces capillary-induced artefacts that can significantly alter the fiber distribution. For these reasons, the analysis was restricted to cross-sections perpendicular to the yarn axis, and the results should be interpreted within this limitation. Despite this constraint, such sections remain highly relevant for applications where transport occurs along the yarn axis—such as liquid imbibition described by the Lucas–Washburn relation^47^—as well as for filtration systems based on nanofibrous yarns, where particle penetration predominantly occurs perpendicular to the yarn axis.

### Curvature and torsion of surface nanofibers

Direct observation of the internal arrangement of nanofibers within a yarn is not yet feasible with current microscopic techniques. However, the structural parameters of the fibers can be estimated from the outer surface layers of the yarn. The crimping of nanofibers on the yarn surface was evaluated using stereological estimates of their average curvature $\bar{{{\boldsymbol{k}}}}$ and average torsion $\bar{{{\boldsymbol{\tau }}}}$, following the approach proposed by De Hoff^48^. The estimators of the average torsion and curvature are defined as

3 $$\left[\bar{\tau }\right]=\frac{\pi }{2}\frac{{I}_{A}}{{N}_{L}},\left[\bar{k}\right]=\frac{\pi }{2}\frac{{T}_{A}}{{N}_{L}},$$

where *I*_A_ represents the number of inflection points per unit area of the projection, *T*_A_ denotes the number of tangent contacts of the test line per unit area, and *N*_L_ is the number of intersections between uniformly random isotropic test lines and the projection of the fibrous system, normalized per unit length of the test line (see Supplementary).

The mean values of curvature and torsion were determined for a PA46 nanofibrous yarn. The SEM micrographs used for this morphological analysis are presented in Fig. 6d, e. The image shown in Fig. 6e is an enlarged view of the region highlighted in Fig. 6d, positioned approximately at the central portion of the yarn surface to minimize potential distortions arising from the yarn’s cylindrical geometry.

The measured average curvature of the surface nanofibers was 0.10 ± 0.02 μm⁻¹, which corresponds to the curvature of a circle with a radius of r = 10.4 μm. The average torsion value of the surface fibers was determined to be 0.032 ± 0.003 μm⁻¹. These structural parameters can be compared to those of a perfect helical fiber, which is characterized by constant curvature *k* and constant torsion *τ*. For such a helix, the following relationships hold

4 $$k=\frac{a}{{a}^{2}+{b}^{2}},\tau =\frac{b}{{a}^{2}+{b}^{2}},$$

where *a* represents the helix radius, and the pitch of the helix equals *2πb*. From these equations, the helix parameters *a* and *b* can be expressed as:

5 $$b=\frac{\tau }{{k}^{2}+{\tau }^{2}},a=\frac{k}{{k}^{2}+{\tau }^{2}}.$$

By substituting the experimentally determined values of curvature and torsion into Eq. (5), the corresponding helix parameters were calculated as *a* = 9.32 μm and *2πb* = 19.70 μm. These values describe a moderately coiled helical geometry of the surface fibers. It can therefore be concluded that such crimped nanofibers on the yarn surface within the untwisted nanofibrous yarn are unable to efficiently transfer stress during the initial stage of deformation, as the applied force is first dissipated through fiber straightening rather than direct load-bearing. The pronounced curvature and torsion of the surface fibers distinguish these nanofibrous yarns from conventional multifilament yarns, which consist of two orders of magnitude fewer, more uniformly oriented filaments of significantly larger diameters. This fundamental structural difference likely underlies the unusual mechanical response observed for nanofibrous yarns.

The estimation of curvature and torsion presented here is based on measurements of nanofibers located at the yarn surface. This approach implicitly assumes that the outer layers are representative, at least qualitatively, of the internal fibrous structure. However, it should be noted that radial variations in fiber packing density, stress state, and local rearrangement may occur between the yarn surface and its core. The reported curvature and torsion values should therefore be interpreted primarily as descriptors of the surface and near-surface structure. A fully resolved characterization of the internal three-dimensional fiber arrangement would require advanced volumetric imaging techniques, which are beyond the scope of the present study.

### Tensile deformation test

**Fig. 7:**
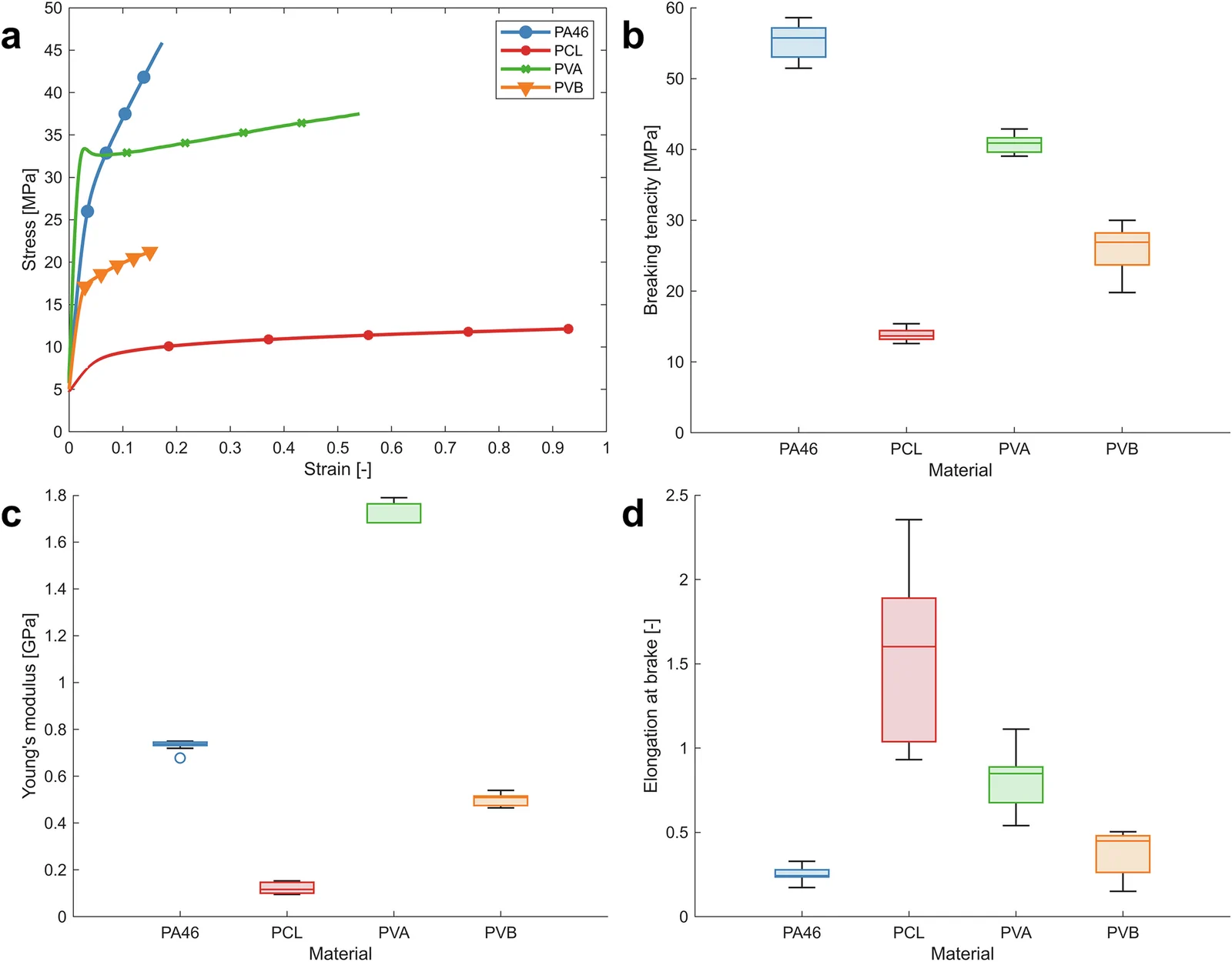
Mechanical properties of nanofibrous yarns. **a** Representative stress–strain curves of PA46, PCL, PVA, and PVB yarns. **b** Box plots of breaking tenacity for each material. **c** Box plots of Young’s modulus, highlighting the high rigidity of PVA compared to the other polymers. **d** Box plots of elongation at break, showing the highest extensibility for PCL yarns and limited elongation for PA46, PVA, and PVB. For **b**–**d**, mechanical properties were determined from *n* = 10 independent yarn measurements per material.

For both practical applications and the processing of nanofibrous yarns into more complex planar or three-dimensional structures, their mechanical properties are of fundamental importance—particularly tensile strength, elongation, and flexibility. Tensile testing revealed pronounced differences in mechanical response across polymer types (Fig. 7). The PA46, PVA, and PVB yarns displayed elastic deformation only within a very small range of relative strain (ε < 0.02), whereas plastic deformation was dominant in the PCL nanofibrous yarn. PA46 reached the highest tensile strength (55 ± 2 MPa), albeit with a relatively low elongation at break. PVA showed lower strength but a substantially higher Young’s modulus (1.71 ± 0.05 GPa), indicative of its high rigidity. By contrast, PCL displayed an exceptionally large strain-to-failure (150 ± 50%) and a correspondingly low modulus and strength. PVB occupied an intermediate regime, combining moderate strength with limited extensibility. Overall, these results demonstrate that variations in polymer chemistry translate into markedly different mechanical behaviors—from brittle-strong (PA46, PVA) to soft-elastic (PCL)—thus providing a broad design space for tuning the functional properties of nanofibrous yarns.

### Braiding of nanofibrous yarns

**Fig. 8:**
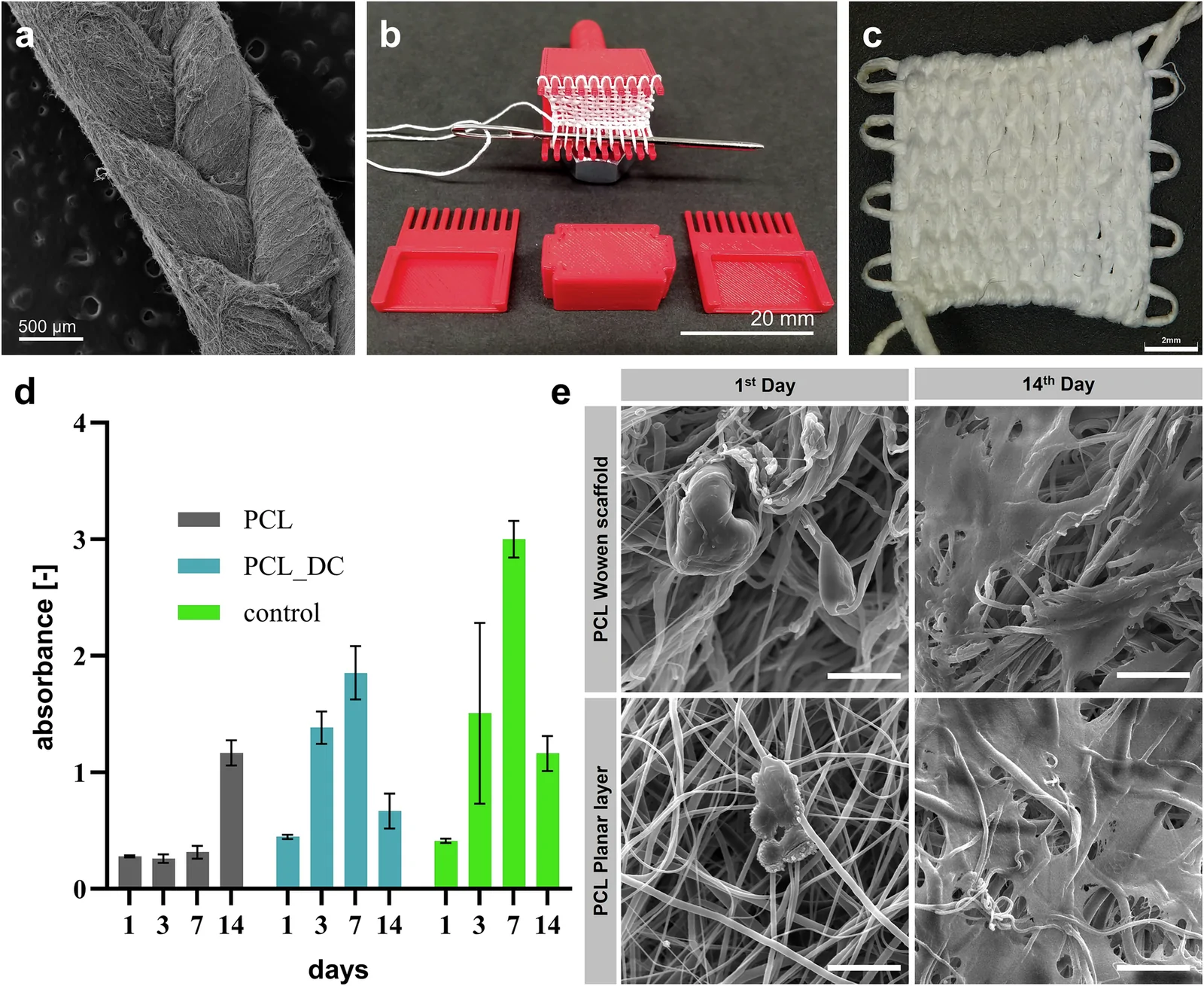
Braiding, weaving, and biological evaluation of nanofibrous yarn scaffolds. **a** SEM micrograph of a braided nanofibrous yarn produced from PVB, showing the ordered interlacing of nanofibrous filaments (scale bar, 500 µm). **b** Custom weaving frame designed for the fabrication of PCL nanofibrous yarn scaffolds, equipped with two 3D-printed combs to ensure uniform yarn spacing (scale bar, 20 mm). **c** Resulting woven PCL scaffold, illustrating the flexibility and structural integrity of the nanofibrous yarn network (scale bar, 2 mm). **d** Cell viability analysis based on absorbance measurements at specified time points. Three substrates were evaluated: a woven PCL scaffold, a planar PCL layer prepared by direct-current electrospinning (PCL_DC), and a control (tissue-culture plastic). The viability of 3T3 fibroblasts was assessed on days 1, 3, 7, and 14 post-seeding. Data are presented as mean ± s.d.; n = 4 independent samples per substrate and time point. **e** SEM images showing distinct cell proliferation and morphology on the woven PCL scaffold compared with the planar PCL layer, confirming enhanced cellular integration and spreading on the 3D nanofibrous structure (scale bar, 20 µm).

To demonstrate the processability of nanofibrous yarns into more complex textile architectures, we fabricated braided yarns, see Fig. 8a. Braided structures are highly attractive for numerous advanced applications due to their combination of mechanical robustness, hierarchical pore organization, flexibility, and design versatility^28^. These characteristics make braided nanofibrous materials particularly suitable for biomedical uses, such as surgical sutures. For example, Wu et al.^49^ reported that nanoyarn-based braided textiles significantly enhanced cell adhesion and proliferation compared with braids made from commercial microfibrous yarns. However, the industrial braiding process involves guiding the yarn through multiple mechanical eyelets and tensioning elements. As a result, braiding nanofibrous yarns without structural damage presents a significant technological challenge, since the delicate nanofibrous sheath is inherently susceptible to abrasion and mechanical disruption.

### Woven tissue-engineering scaffolds

Tissue engineering relies on the coordinated interplay between biomaterial scaffolds, cells, and bioactive molecules. Nanofibrous scaffold architectures are engineered to recapitulate key features of the native extracellular matrix (ECM), thereby supporting cell adhesion, migration, proliferation, and differentiation^50^. Woven textiles produced from nanofibrous yarns enable precise control over pore size, porosity, and mechanical response by tuning yarn characteristics—fineness, diameter, twist, and extensibility—and by adjusting weaving density^51,52^. Here, we fabricated woven scaffolds from PCL nanofibrous yarns (Fig. 8b, c). The warp density was 9–10 yarns·cm⁻¹ and the weft density 18–20 yarns·cm⁻¹, producing an open, porous architecture that facilitates nutrient transport and cell infiltration. Each scaffold measured ~10 × 10 mm, dimensioned to fit 12-mm culture wells, and was constructed from nanofibrous yarns with a linear density of 62.50 tex. These woven constructs were subsequently used as tissue-engineering scaffolds in cell-culture assays and mechanical testing.

### Cell proliferation assay

Poly(ε-caprolactone) (PCL) was selected as a model polymer for biological evaluation due to its well-established biocompatibility and widespread use in tissue engineering. The purpose of the experiment was to assess the effect of scaffold architecture—woven nanofibrous yarns versus planar electrospun layers—on cell behavior, rather than to compare different polymer chemistries. By maintaining a constant material composition, the influence of structural organization on cell adhesion and proliferation could be isolated. The interaction of 3T3 mouse fibroblasts with the tested materials was evaluated on two types of PCL substrates: (i) woven scaffolds fabricated entirely from nanofibrous yarns, and (ii) planar nonwoven nanofibrous layers produced by direct-current (DC) electrospinning of the same polymer. The well bottom of the culture plate served as the standard control. In total, 20 woven and 20 planar samples were prepared for testing. The planar nanofibrous mat consisted of nano- and microfibers with an average diameter of 700 ± 830 nm, reflecting the broad diameter distribution arising from the selected solvent system. A comparable structural heterogeneity was also observed in the nanofibrous yarns, which consisted predominantly of micron-scale fibers with a similarly wide diameter distribution (Fig. 4). Therefore, the comparison between planar and woven scaffolds primarily reflects differences in structural organization rather than fiber size distribution. Initially, cell adhesion was markedly lower on the woven scaffolds than on both the planar nanofibrous layers and the tissue-culture plastic control (Fig. 8d). This behavior can be attributed to the open-mesh architecture of the woven scaffolds, which allows cells to penetrate through the pores and settle on the bottom of the culture well, preventing complete surface coverage during the initial adhesion phase. As shown by the absorbance-based viability assay, the woven scaffolds exhibited slow initial proliferation up to day 7, followed by a phase of exponential cell growth observed on day 14. In contrast, both the planar DC-spun nanofibrous layer and the control surface of the culture plate supported rapid proliferation within the first few days post-seeding, reaching a plateau and showing a decrease in viability by day 14, likely due to full confluence. Scanning electron microscopy analysis confirmed the formation of a confluent fibroblast layer on all tested materials after 14 days, in agreement with the results of the cell viability assay (Fig. 8e).

## Discussion

This work demonstrates a robust, high-throughput route to continuous core-free NFYs by coupling needleless AC electrospinning with a false-twist twirling unit. The neutral, cohesive nanofiber plume produced by AC electrospinning enables direct mechanical take-off without an electrically active collector, supporting yarn formation across diverse polymers and production speeds up to 55 m·min⁻¹. The maximum attainable speed is governed by plume stability, with mechanical rupture occurring near withdrawal velocities of ~100 m·min⁻¹. Fineness of NFYs in the tens to hundreds of tex is readily accessible through coordinated adjustment of take-off and winding speeds, highlighting the versatility of the manufacturing regime. Considering that many biomedical devices—grafts, sutures, ligament or tendon substitutes, nerve conduits—require lengths of meters to tens of meters, the demonstrated production speeds are already compatible with practical manufacturing demands.

Mechanistic analysis shows that axial drawing within the spinning triangle is negligible, as fibers converge along similar path lengths per unit time. Yarn orientation is therefore determined upstream—by plume dynamics and take-off kinematics—rather than by in-triangle stretching. Increasing drum speed enhances nanofibre alignment but also reduces linear density and thus absolute strength, revealing a trade-off that can be resolved only by increasing nanofiber mass flux from the spinning electrode.

The process is inherently scalable: enlarging disc diameter, deploying multi-disc or wire-based electrodes can increase output while maintaining reliable yarn formation. Twist instabilities seen at the highest speeds can be mitigated through improved friction management and active tension control, and the integration of in-line imaging and feedback regulation represents a natural step toward industrial robustness.

Microstructural analyses reveal deformation mechanisms distinct from conventional multifilaments. Moderately coiled helical nanofibers on yarn surfaces straighten during early tensile loading, explaining the hierarchy of mechanical behavior and providing a basis for systematic tuning. Post-processing further expands this design space; for instance, converting smooth PCL fibers to shish–kebab morphologies markedly increases surface area and offers routes to modulating adhesion, mechanics, and functionalization.

Morphological analysis revealed that the produced yarns consist of densely packed nanofibers with characteristic diameters of approximately 220 ± 60 nm (PA46), 410 ± 150 nm (PVA), 870 ± 250 nm (PVB), and 1140 ± 490 nm (PCL), with the latter showing a broader distribution extending into the microscale. Cross-sectional analysis of PVB yarn yielded a fiber number density of *N*_S_ ≈ 0.106 μm⁻² and a most probable pore radius of *r*_p_ ≈ 1.25 μm, confirming a fine and interconnected porous architecture.

The internal structural organization of the yarn was further characterized through stereological analysis of surface nanofibers, yielding an average curvature of ~0.10 μm⁻¹ and torsion of ~0.032 μm⁻¹. These values correspond to a moderately coiled helical geometry of the fibers, which plays a key role in the mechanical response of the yarn, particularly during the initial deformation stage dominated by fiber straightening.

Mechanical testing demonstrated a wide tunability of properties depending on polymer type, ranging from high-strength, low-elongation behavior in PA46 ( ≈ 55 MPa tensile strength) to highly extensible responses in PCL (elongation up to ≈150%). This variability highlights the flexibility of the approach in tailoring yarn performance for specific applications.

Finally, the performance of 3D woven NFY scaffolds demonstrates the tissue engineering potential of this platform. Although these constructs showed delayed early adhesion due to initial cell settlement through the pores, proliferation progressed rapidly thereafter and reached confluence by day 14, comparable to planar controls. This decoupling of early occupancy from later 3D colonization underscores the unique architectural control available through yarn fineness, twist, and textile topology.

In conclusion, the AC-electrospinning/false-twist strategy bridges nanoscale fiber formation with macroscale textile manufacturing, delivering scalable, core-free nanofibrous yarns with tuneable structure, topology, and mechanics. We anticipate that this approach will not only accelerate the industrial-scale production and adoption of nanofibrous yarns, but also stimulate further research into their properties, processing, and applications.

## Methods

### Materials

Poly(vinyl butyral) (PVB; Mowital® B 60 H, average molecular weight 60,000 g·mol⁻¹) was obtained from Kuraray (Japan). Polyamide 46 (PA46; Stanyl® TW341) was supplied by Envalior (Germany). Poly(ε-caprolactone) (PCL; PURASORB® PC 08, average molecular weight 45,000 g·mol⁻¹, PCL45) was purchased from Corbion (Netherlands). PCL with an average molecular weight of 80,000 g·mol⁻¹ (PCL80), poly(vinyl alcohol) (PVA; Mowiol® 20-98, average molecular weight 125,000 g·mol⁻¹), and pentyl acetate ( ≥ 99%) were obtained from Sigma-Aldrich (Germany). Ethanol was purchased from VWR Chemicals (Germany), while acetic acid (AA; 99.8% p.a.), formic acid (FA; 98% p.a.), and acetone were supplied by PENTA (Czech Republic).

### Preparation of polymer solutions

A 13 wt% PVB solution was prepared by dissolving PVB in ethanol. A 14 wt% PA46 solution was prepared using a 1:1 (w/w) mixture of AA and FA. Both PVB and PA46 solutions were magnetically stirred at 400 rpm and room temperature (22 ± 1 °C) for 12 h to ensure complete dissolution. PVA was dissolved in deionized water to obtain a 10 wt% solution, stirred at 90 °C and 400 rpm for 24 h until fully dissolved. A 13 wt% PCL solution was prepared from a blend of PCL45 and PCL80 (1:2 w/w) using a ternary solvent mixture of AA, FA, and acetone (5:6:4 w/w/w). The mixture was stirred at 45 °C and 400 rpm for 4 h before electrospinning. For surface shish-kebab modification, 1 wt% solutions of PCL45 and PCL80 were prepared separately in pentyl acetate at 400 rpm at 45 °C for 12 h until partially dissolved.

### AC electrospinning apparatus

**Fig. 9:**
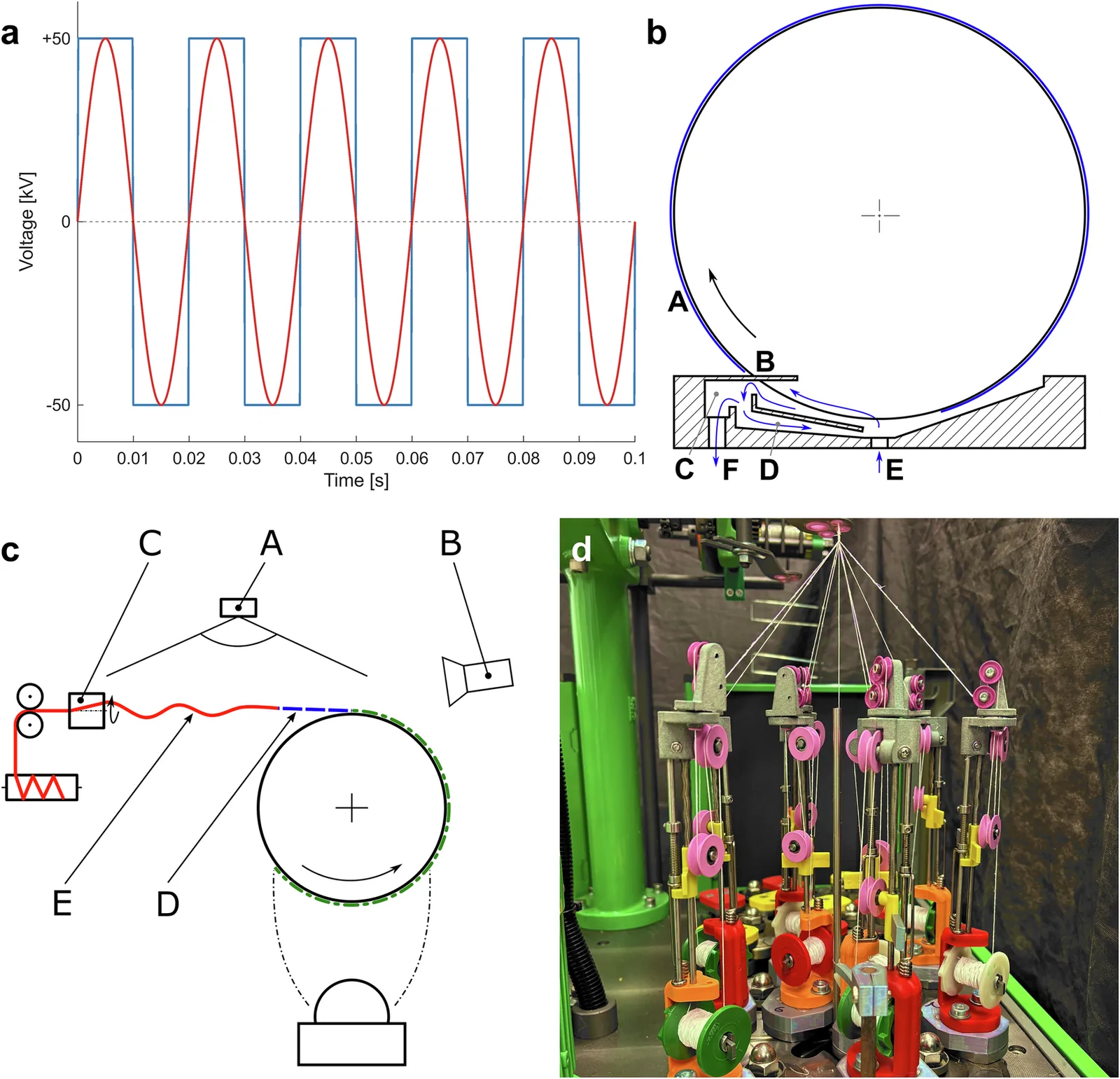
AC electrospinning setup, spinning triangle imaging arrangement and braiding machine. **a** Voltage waveforms for AC electrospinning generated by the TREK 50/12 high-voltage amplifier. Waveform shapes illustrating a sinusoidal (red) and a step-function (blue) input signal, both at 50 Hz. **b** Schematic illustration of the spinning electrode and polymer solution circulation system: (A) liquid film of the polymer solution on the disc surface; (B) passage of the disc through the wiping slit; (C) overflow of the polymer solution; (D) recirculation of part of the solution onto the disc edge; (E) supply of the polymer solution from an external reservoir; and (F) closed-loop return of the solution driven by a gear pump. **c** Schematic of the imaging setup for observing the spinning triangle and yarn formation: (A) camera for twist-triangle observation; (B) illumination source; (C) twisting device; (D) spinning triangle; and (E) ballooning yarn. **d** Braiding machine EY-RA-8-4 (110 II) equipped with an eight-carrier arrangement, used for fabricating braided nanofiber yarns. The original ceramic eyelet guides were replaced with pulleys to minimize friction.

A custom-designed needleless alternating current (AC) electrospinning setup was used for all experiments. The system employed a VTS 38 high-voltage transformer (KPB Intra, Czech Republic) with a conversion ratio of 35,000 V/230 V. The transformer was powered and controlled by a programmable Kikusui PCR1000LE power supply (Kikusui Electronics, Japan). The applied voltage waveform was user-defined as a superposition of the first, third, and fifth sinusoidal harmonics with an amplitude ratio of 1: 0.22: 0.05. For all experiments, the root-mean-square (RMS) input voltage to the transformer was maintained at 304–308 V, corresponding to an RMS output voltage of 45.1–46.8 kV at the spinning electrode. The peak voltage reached 57 kV (measured) and 53.7 kV (theoretical). Alternatively, high-voltage waveforms can be generated using a high-voltage amplifier TREK 50/12 (Advanced Energy, USA), which provides precise control of AC output in the range 0 to ±50 kV. This device enables amplification of externally supplied waveforms, including sinusoidal and step-function signals (Fig. 9a).

The disc-type spinning-electrode was developed at the Technical University of Liberec (Czech Republic). The disc had a diameter of 200 mm and a thickness of 1 mm in the spinning region. It was partially immersed in the polymer solution reservoir, with approximately three-eighths of its circumference exposed as the fiber-spinning zone from which multiple polymer jets were generated. The polymer solution was continuously supplied to the disc edge by the disc rotation (0.1–100 rpm) through the polymer bath. The reservoir was equipped with a 2.5 mm-wide slit through which the disc passed, ensuring a uniform liquid film on its circumference during all experiments. The solution level in the reservoir was maintained by an overflow system that kept the liquid height constant (Fig. 9b). Owing to the small working volume of the reservoir, the polymer solution was continuously circulated from an external tank using a gear pump (RM-GJ-N25, Micropump Inc., USA), while the excess solution was returned via a secondary gear pump, forming a closed circulation loop. Polymeric solution in the external reservoir could be continuously stirred using a magnetic stirrer to maintain homogeneity. All components of the polymer solution handling system were designed, insulated, and grounded to ensure safe operation under high alternating voltage.

The plume of nanofibers generated from the disc spinning electrode was carried radially outward by the electric wind and deposited onto a rotating drum collector (diameter 430 mm), forming a continuous nanofiber strip 100–300 mm in width. The collector (Technical University of Liberec, Czech Republic) was positioned 230 mm above the disc electrode. The same geometric configuration was maintained for all experiments to ensure reproducibility of the electrospinning process.

The formation of the spinning triangle and the twisting of the yarn were achieved using a custom-built twirling device developed at the Technical University of Liberec (Czech Republic). The device operated at rotational speeds ranging from 100 to 20,000 rpm and was positioned tangentially to the upper part of the rotating drum collector. The twirling mechanism consisted of eccentric eyelet/eyelets (Ascotex, United Kingdom) through which the forming yarn was guided. To increase the maximum momentum transfer while maintaining the same eyelet eccentricity, a double-eyelet version of the twirling device was also designed and employed (Fig. 9c).

The process parameters used in this study were selected based on a combination of prior experience with AC electrospinning systems and systematic preliminary experiments aimed at identifying a stable operating regime for continuous yarn formation (tab. 1 and tab. 2). Electrical parameters (voltage amplitude, frequency, and electrode–collector distance) were initially chosen according to established AC electrospinning conditions and subsequently refined to ensure stable plume formation. Mechanical parameters governing yarn formation, such as the rotational speed of the twirling device, drum surface velocity, and take-up speed, were adjusted iteratively to achieve a stable spinning triangle and continuous yarn production. The process operates within a stability window defined by the balance between nanofiber production rate, adhesion to the collector, and torque-driven yarn formation. Within this window, moderate variations in parameters do not significantly affect process stability.

### Video recording of the spinning triangle

The twisting region of the nanofiber ribbon was recorded using a imaging system to analyze the formation and dynamics of the spinning triangle. A schematic of the experimental arrangement is shown in Fig. 9c. The region between the drum collector and the twirling device was illuminated by a high-intensity LED light source (Andoer LM135Bi, 135 W, color temperature 5600 K) and imaged using a Camera Module 3 (Raspberry Pi Foundation) connected to a Raspberry Pi microcomputer. The camera enabled Full HD video acquisition and still image capture at a resolution of up to 12 MP. Its key advantage is the full programmability of all imaging parameters, allowing precise control of exposure time, gain, and frame rate. To achieve high contrast between the nanofiber structure and the background, all visible mechanical components were coated in a dark matte color, whereas the imaging region was illuminated in the direction of yarn motion. This illumination geometry enhanced the visibility of the nanofiber surface texture and the evolving shape of the spinning triangle.

### Post-process treatment

Pristine PCL nanofiber yarns were stretched and mounted onto a bamboo embroidery hoop (diameter 180 mm) to maintain uniform tension along their length. The mounted yarns were then uniformly sprayed with a 1 wt% PCL45 or PCL80 solution prepared in pentyl acetate using a fine mist spray bottle until fully wetted, representing a shish–kebab-type post-treatment process. Excess liquid was gently removed from the yarns using filter paper to prevent fiber displacement and abundand swelling. Finally, the nanofiber yarns were air-dried under laboratory conditions (22 ± 2 °C, 50 ± 5 % relative humidity) inside a fume hood.

### Scanning electron microscopy investigation

Scanning electron microscopy (SEM) was used to examine the surface morphology, fiber orientation, and shish–kebab structures of the nanofibrous yarns. Samples were carefully mounted on aluminum stubs using conductive carbon adhesive tape to ensure stable fixation and reliable electrical contact. Prior to imaging, all specimens were sputter-coated with a ≈ 10 nm thick layer of gold using a Q150RS Plus sputter coater (Quorum Technologies, UK) to minimize surface charging and enhance image contrast. SEM images were acquired using a VEGA3 SBU EasyProbe microscope (Tescan, Czech Republic) equipped with a tungsten filament electron source and an Everhart–Thornley secondary electron detector (YAG scintillator). Imaging was performed in high-vacuum mode (SE) at an accelerating voltage of 15 kV. Detailed visualization and characterization of the shish–kebab structures were performed using a Helios 5 PFIB Cxe microscope (Thermo Fisher Scientific, Czech Republic). Representative areas were selected from multiple positions along each yarn to ensure statistical significance. Fiber diameters were determined in ImageJ software, with mean values calculated from 300 individual measurements obtained from five SEM micrographs per sample.

Fiber anisotropy and orientation were further analyzed in ImageJ using the *DiameterJ* and *OrientationJ* plugins^53^, enabling automated quantification of fiber alignment distributions within the nanofibrous yarns. The local orientation of nanofiber segments was computed from the coresponding binarized SEM micrograph and expressed as an angular distribution in the range 0–180°, accounting for the axial symmetry of fiber orientation. The orientation data were represented as directional histograms (“roses of directions”), where the frequency of fiber segments falling within each angular bin was evaluated. The principal rose vector was defined as the vector corresponding to the dominant orientation, i.e., the angular bin with the maximum frequency. The direction of the vector is given by this dominant angle, while its magnitude is proportional to the normalized maximum frequency. Normalization was performed with respect to the total number of detected fiber segments in the analyzed region, allowing direct comparison between different spatial locations within the spinning triangle. This representation provides a compact visualization of both the preferred orientation and the degree of alignment of the nanofibrous structure.

The porosity of the nanofibrous structure was evaluated from the spatial distribution of fiber endpoints identified in cross-sectional micrographs. The endpoint density was determined for each image and used as a stereological descriptor of the network. To characterize the pore size distribution beyond the assumption of complete spatial randomness, i.e., the Poisson point process, an numerical approach based on Monte Carlo sampling was employed. For each image, a set of 10^6^ random test points was uniformly distributed within the observation window. For each test point, the radius of the largest empty circle not intersecting any fiber endpoint was determined as the minimum distance to the nearest endpoint. The resulting set of radii was used to estimate the pore size probability density function, from which the most probable pore size was obtained. Edge effects were taken into account in this procedure. Final values were obtained as weighted averages of the individual image-level results, with weights proportional to the number of detected endpoints.

### Tensile tests

Tensile properties of the nanofibrous yarns were evaluated using a LabTest 6.0051 universal testing machine (LaborTech, Czech Republic) equipped with manual capstan grips and a 10 N load cell (AST, Germany). The load cell complied with EN ISO 7500-1, providing accuracy class 1 from 0.030 N and class 0.5 from 0.1 N. Force and elongation data were recorded at a sampling frequency of 50 Hz. Each test was conducted with an initial gauge length of 250 mm, a crosshead speed of 250 mm·min⁻¹, and a preload of 15 cN to ensure uniform yarn alignment and tension prior to loading. All measurements were performed under controlled laboratory conditions (22 ± 2 °C, 50 ± 5 % relative humidity). For each yarn type, the average tensile strength and elongation at break were calculated as the mean values from ten independent specimens.

### Braiding machine

Braided nanofibrous threads were fabricated using an EY-RA-8-4 (110 II) braiding machine (Hsiang Chuan Machinery, Taiwan) equipped with eight custom-modified yarn carriers (Technical University of Liberec, Czech Republic) designed to handle fine and mechanically sensitive nanofibrous yarns. The braiding system comprised eight peripheral carriers and a central yarn guided through a fixed unwinding device, which was not mechanically braided by the carriers. This configuration produced a balanced and symmetric braid structure. Eight peripheral nanofibrous yarn was mounted on an individual carrier under precisely controlled tension to prevent filament damage and to ensure uniform interlacing during the braiding process. The production speed of the braided threads ranged from 0.09 to 0.25 m·min⁻¹, depending on the yarn composition and its stability during continuous operation. The resulting braids exhibited a nominal diameter of 1.5–1.7 mm, and the braid angle—defined as the angle between the yarn path and the braid axis—was approximately 15–25°, as determined from SEM micrographs of the braided structures (Fig. 8a). These parameters resulted in a compact yet flexible braid architecture suitable for subsequent mechanical and morphological analyses.

### Woven scaffolds for tissue-engineering assays

Woven nanofibrous scaffolds were fabricated using a custom miniature weaving frame assembled from 3D-printed comb elements. Each scaffold was produced from two nanofibrous yarn segments (20–25 cm each). The first yarn was threaded in a zigzag pattern behind alternating comb teeth (1 mm spacing) to form the warp, while the second yarn served as the weft and was interlaced through the warp using a fine sewing needle to generate a plain-weave structure. Manual insertion of the weft allowed precise yarn placement while minimizing mechanical stress on the fragile nanofibrous filaments. After weaving, the constructs were gently released from the comb frame to avoid deformation. The total fabrication time for a single scaffold was approximately 15–20 minutes.

### Cell proliferation assay

NIH/3T3 mouse fibroblasts (ATCC CRL-1658; American Type Culture Collection, USA) were used to evaluate cell adhesion and proliferation on the nanofibrous scaffolds. Cells were cultured in Dulbecco’s modified Eagle’s medium (DMEM) supplemented with 10% fetal bovine serum (FBS) and maintained at 37 °C in a humidified atmosphere containing 5% CO₂. Cell adhesion and proliferation were evaluated on days 1, 3, 7, and 14 after seeding. Cell growth was quantified using the Cell Counting Kit-8 assay (CCK-8; Dojindo, Germany), and cell morphology and viability were examined by fluorescence microscopy and scanning electron microscopy. On each sampling day, five specimens from each material type were collected for analysis, comprising four cell-seeded specimens and one unseeded control. Cell viability and proliferation were assessed using a 10% CCK-8 solution in complete culture medium. Samples were incubated for 2 h at 37 °C in a CO₂ incubator and subsequently fixed in 2.5% glutaraldehyde in phosphate-buffered saline (PBS) for microscopic analysis by SEM and FM.

The NIH/3T3 cell line was obtained directly from ATCC, and no additional in-house authentication was performed. According to the supplier’s quality-control information, mycoplasma contamination was not detected; no additional in-house mycoplasma testing was performed. No commonly misidentified cell lines were used in this study.

Cells adhering to woven and planar scaffolds were stained with DAPI (nuclei; Merck, Czech Republic) and Phalloidin–FITC (actin filaments; Merck, Czech Republic). The stained samples were examined using an Axio Observer fluorescence microscope (Zeiss, Germany) at 10× and 20× magnifications. For imaging, samples were placed on standard glass slides; woven scaffolds were additionally secured between two slides joined with adhesive tape to prevent deformation during observation. For detailed evaluation of cell adhesion and proliferation, SEM analysis was performed. After fixation, samples were dehydrated in a graded ethanol series (60%, 70%, 80%, 90%, 96%, and 100%) and air-dried on Parafilm.

## Supplementary information

Transparent Peer Review file

Supplementary Information

Description of Additional Supplementary Files

Supplementary Movie 1

Supplementary Movie 2

Supplementary Movie 3

Supplementary Movie 4

## Data Availability

The experimental data that support the findings of this study are available in zenodo repository (DOI: 10.5281/zenodo.17812759)^54^.
