# Supplementary material for: High-throughput fabrication of nanofibrous yarns produced by alternating-current electrospinning
Source: Commun Eng. 2026 Jul 22;5:196. doi: 10.1038/s44172-026-00737-x (PMC13759490; doi:10.1038/s44172-026-00737-x)
Supplement: Supplementary file 1 [file 44172_2026_737_MOESM1_ESM.pdf]

nature portfolio

Peer Review File

High-throughput fabrication of nanofibrous yarns produced by alternating-current electrospinning

Corresponding Author: Mr Jaroslav Mikule

This file contains all reviewer reports in order by version, followed by all author rebuttals in order by version.

Attachments originally included by the reviewers as part of their assessment can be found at the end of this file.

Version 0:

Reviewer comments:

Reviewer #1

(Remarks to the Author)
This article reports the development of a high-throughput alternating-current (AC) electrospinning strategy for the continuous fabrication of fully nanofibrous yarns with controlled morphology and mechanical performance. The manuscript reports significant and potentially publishable results; however, the organization and placement of several sections make the presentation difficult to follow. In addition, the following issues should be addressed.

• The introduction is well written and logically structured, but several claims require moderation, clarification of scope, and more careful attribution of prior work.

• A clear overview of AC electrospinning and nanofiber yarns is provided in introduction section; however, the manuscript does not explicitly articulate the research gap or clearly distinguish the novelty of the present work from prior studies. While AC electrospinning, high-throughput fiber production, and nanofiber yarn fabrication have all been reported previously, it remains unclear which specific limitations of existing approaches are addressed here. The authors are encouraged to explicitly define the unmet challenges in current AC electrospinning-based yarn fabrication.

Below are some of the relevant literature:

o Divyabharathi Madheswaran, Manikandan Sivan, Sarka Hauzerova, Eva Kuzelova Kostakova, Vera Jencova, Jan Valtera, Lubos Behalek, Jana Mullerova, Nhung H.A. Nguyen, Lukas Capek, David Lukas, Continuous fabrication of braided composite nanofibrous surgical yarns using advanced AC electrospinning and braiding technology, Composites Communications, Volume 48, 2024, https://doi.org/10.1016/j.coco.2024.101932.

o Mikule J, Friedrich O, Aguilera JP, Zabka P, Kostakova EK, Valtera J, Hauzerová Š, Skrivanek J, Jencova V, Batka O, Meszaros L. High throughput fabrication of nanofibrous yarns produced by AC electrospinning.

o Manikandan Sivan, Divyabharathi Madheswaran, Jan Valtera, Eva Kuzelova Kostakova, David Lukas, Alternating current electrospinning: The impacts of various high-voltage signal shapes and frequencies on the spinnability and productivity of polycaprolactone nanofibers, Materials & Design, Volume 213, 2022, https://doi.org/10.1016/j.matdes.2021.110308.

• In Section “Results” on page 3, line 93-97, the manuscript attributes plume propagation primarily to “electric wind.” This statement would benefit from further clarification or supporting references, as plume dynamics in AC electrospinning are known to depend on multiple factors including field frequency, waveform, and solution properties.

• On page 5, line no 163, it is stated that torque is transmitted “only through the nanofiber mass itself, without physical contact with any external tool.” This is an important and non-intuitive claim, yet it is presented qualitatively. The authors should clarify the physical basis of torque transfer within the nanofiber belt and discuss whether this mechanism has limits with respect to belt thickness or fiber density.

• Page 5, line 165, the assertion that the twisting torque simultaneously provides the tensile force required for detachment is interesting but insufficiently proven. The authors should clarify whether this coupling is intrinsic to the system geometry or dependent on specific process parameters.

• Page 5 & 6, several mechanistic explanations rely on qualitative assumptions derived from conventional yarn mechanics. Incorporating quantitative parameters, clarifying optimization choices, and providing experimental evidence for the stabilizing role of the galette would significantly strengthen the rigor and reproducibility of this part of the manuscript.

• Page 6, line 207, the proposed contributions of mechanical interlocking, van der Waals forces, and electrostatic attraction are described as assumptions rather than experimentally verified mechanisms. While reasonable, these assumptions should be explicitly framed as hypotheses or supported with references, measurements, or indirect evidence.

• The authors provide a list of process parameters; however, the manuscript does not explain how these parameters were

selected or optimized. Please clarify whether they were chosen based on prior literature, preliminary experiments, or systematic optimization, and discuss how sensitive the process is to variations in these parameters.

• Page 9, the use of principal rose vectors is appropriate for visualizing orientation trends; however, the manuscript does not clearly define how these vectors are calculated or normalized. A brief methodological description or reference would improve reproducibility and clarity.

• Page 12, section “Fiber density and pore size distribution.”, the probability density function used to derive the pore radius implicitly assumes a spatially random and isotropic distribution of fibers. Given that the manuscript later demonstrates pronounced fiber alignment and anisotropy within the yarn, the validity of applying an isotropic pore model should be critically addressed.

• The pore size calculation is based on a single SEM cross-section containing 279 fibers within a reference area of 761.76 μm^2. It is unclear whether this region is representative of the entire yarn cross-section or whether multiple locations and yarns were analyzed. The authors should clarify the number of samples and sections evaluated to ensure statistical robustness.

• The manuscript transitions from internal pore statistics to surface fiber curvature and torsion without explicitly justifying how surface measurements reflect internal yarn structure. Given that yarn compaction and stress states may vary radially, this assumption should be stated and discussed more explicitly.

• Caption of Figure 9, “AC electrospinning setup and imaging arrangement” does not describe the Figure 9(d) in its title.

• On page 18, in section “Cell proliferation assay “ the comparison between woven nanofibrous yarn scaffolds and planar DC-electrospun mats is interesting; however, the rationale for selecting only PCL for the biological evaluation is not clearly justified. The authors should clarify whether PCL was chosen as a representative system or whether similar trends are expected for other polymers used in the study.

• On page 18, in section “Cell proliferation assay “ , the planar nanofibrous mat is described as consisting of both nano- and microfibers with a very broad diameter distribution (700 ± 830 nm). Such heterogeneity may significantly influence cell behavior. The authors should discuss how this structural variability affects the comparison with the woven scaffold and whether fiber diameter was intentionally controlled.

• The conclusion section is very brief and does not sufficiently summarize the key findings of the study. In particular, it lacks a clear synthesis of the main quantitative results and performance metrics achieved in this work. The manuscript would benefit from a more elaborate conclusion presented as a dedicated section, highlighting the most significant outcomes, numerical values, and their implications for future applications.

Reviewer #2

(Remarks to the Author)
I co-reviewed this manuscript with one of the reviewers who provided the listed reports. This is part of the Communications Engineering initiative to facilitate training in peer review and to provide appropriate recognition for Early Career Researchers who co-review manuscripts.

Reviewer #3

(Remarks to the Author)
This manuscript presents an interesting and well-executed study on the continuous fabrication of core-free nanofibrous yarns using AC electrospinning. The topic is relevant, the processing concept is technically strong, and the work addresses an important challenge in the field: the transition from nanofiber production to more practical, textile-compatible 1D structures.

The study is clearly beyond a simple proof of concept. The authors demonstrate yarn formation for several polymers, provide morphological and mechanical characterization, and further show the possibility of braiding, weaving, and preliminary biological evaluation. Overall, I find the manuscript valuable and suitable for publication after minor revision.

My main comment concerns the way scalability is currently presented. The reported production rates are promising and clearly indicate strong scale-up potential. At the same time, the current manuscript would benefit from slightly more careful wording in this section, as the experimental evidence remains based on relatively limited operating time and a restricted set of processing conditions.

To further strengthen the manuscript, I recommend that the authors address the scalability claim in a more precise, quantitative way. This could be done through a few relatively small additions:

1/ Add a brief comment on reproducibility.
Even a brief statement indicating whether the reported yarn production was reproduced in independent runs would strengthen the work's engineering relevance. If available, the authors could report the number of replicate experiments and whether similar yarn quality and process stability were consistently achieved.

2/Clarify the distinction between routine operating speed and maximum demonstrated speed.
In the current version, the manuscript reports routine production rates for different polymers and also mentions a maximum speed of 55 m min^{-1} for PVB. It would help the reader if the authors explicitly separate these two cases and indicate more clearly which conditions correspond to stable standard operation and which represent the upper performance limit of the setup.

3/ Provide a short note on continuous-operation stability.
The manuscript already states that continuous production for at least one hour was achieved and shows a spool as evidence. It would be useful to expand this slightly by indicating whether this stability was observed only under selected conditions or more generally across the tested polymer systems (e.g., only for 1 polymer, just for show).

4/. Slightly moderate the wording where appropriate.
If additional quantitative data are not available, I would suggest using slightly more cautious phrasing, such as “shows strong scale-up potential” or “represents a promising route toward scalable production,” rather than making broader claims of scalability or industrial readiness at this stage. The current data clearly support the method's promise, but it has not yet undergone full engineering validation at an industrial level.

Aside from this point, the manuscript is well structured, and the results are presented clearly. I do not see any major scientific weakness that would require substantial additional experiments. The suggested revision is intended primarily to improve the precision of the results' interpretation.

In summary, this is a strong manuscript with clear practical relevance for the nanofiber and textile engineering communities. I recommend publication after minor revision.

Version 1:

Reviewer comments:

Reviewer #1

(Remarks to the Author)
Overall, the revised manuscript clearly improves the manuscript in terms of scientific rigor, scope clarification, and reproducibility. However, the revised version of manuscript does not fully address the main concern regarding the explicit identification of the research gap and novelty of the present work. While additional references were incorporated, the revised text still lacks a clear statement explaining:
• what specific limitations remain in previously reported AC electrospinning-based nanofiber yarn fabrication approaches,
• and what unique scientific or technological advancement is introduced in the present study.

Reviewer #2

(Remarks to the Author)
I co-reviewed this manuscript with one of the reviewers who provided the listed reports. This is part of the Communications Engineering initiative to facilitate training in peer review and to provide appropriate recognition for Early Career Researchers who co-review manuscripts.

Reviewer #3

(Remarks to the Author)
The article has been revised as recommended. I have no further comments.

Open Access This Peer Review File is licensed under a Creative Commons Attribution 4.0 International License, which permits use, sharing, adaptation, distribution and reproduction in any medium or format, as long as you give appropriate credit to the original author(s) and the source, provide a link to the Creative Commons license, and indicate if changes were made.
In cases where reviewers are anonymous, credit should be given to 'Anonymous Referee' and the source.
The images or other third party material in this Peer Review File are included in the article's Creative Commons license, unless indicated otherwise in a credit line to the material. If material is not included in the article's Creative Commons license and your intended use is not permitted by statutory regulation or exceeds the permitted use, you will need to obtain permission directly from the copyright holder.
To view a copy of this license, visit https://creativecommons.org/licenses/by/4.0/

We would like to sincerely thank the reviewer for their careful reading of the manuscript and for the constructive comments and suggestions, which have significantly contributed to improving the clarity and quality of the text.

Reviewers' comments:

Reviewer #1 (Remarks to the Author):

• **Comment 1.** The introduction is well written and logically structured, but several claims require moderation, clarification of scope, and more careful attribution of prior work.

Response to Reviewers comment no. 1:

We agree that several statements in the Introduction were too broad or insufficiently delimited in scope. In response, we revised the Introduction in three ways.

First, we moderated claims that could be read as overly general or overly strong.

Second, we clarified the scope of comparison by specifying when statements refer specifically to reported nanofiber-yarn fabrication routes.

Third, we improved attribution of prior work by more clearly distinguishing foundational studies.

We also revised wording that could be interpreted as universal across all electrospinning platforms, replacing such statements with language specific to NFY-forming approaches where appropriate.

Revised manuscript paragraphs:

Direct-current (DC) electrospinning remains the most established route for producing nanofibers and has also been widely used for nanofiber-yarn (NFY) fabrication. [9]. Building on the foundational work of Formhals [10–12], decades of innovations—including conjugated electrostatic spinning and multi-nozzle systems—have improved yarn uniformity and mechanical stability [13–18]. However, many reported DC-based NFY fabrication strategies remain constrained by relatively low solution feed rates and modest collection speeds, with representative yarn take-up speeds often below 2 m min^{-1} and reaching about 10 m min^{-1} in post-drawing-assisted configurations. [19,20]. These constraints hinder scalability and motivate alternative high-throughput approaches.

An important advance was the introduction of collectorless alternating-current (AC) electrospinning by Lukáš and co-workers [21], later reviewed by Stanishevsky [22]. AC electrospinning exploits alternating jet polarity to generate periodic self-neutralization of fibres, forming a transient *virtual collector* several centimetres from the electrode [21]. The dynamics of this charge-reduced zone—governed by field frequency and waveform—strongly affect fibre trajectories and plume morphology [23] and, when combined with needleless electrodes can enable substantially higher fibre production rates than many conventional DC NFY-forming approaches. This principle also enabled composite NFYs formed by wrapping an AC-generated

plume around a core thread [24], combining nanoscale functionality with macroscale mechanical integrity.

NFYs, including composite and core-less variants, have attracted increasing interest in biomedicine due to their resemblance to the extracellular matrix (ECM). Their fibrous architecture promotes cell attachment and growth, making them promising scaffolds and drug-delivery platforms [25–27]. Hollow NFYs can provide prolonged therapeutic release [28], and direct incorporation of bioactive molecules enables multifunctional regenerative systems [29].

However, NFY production is still restricted by the low throughput of DC electrospinning and by limited control over yarn diameter, linear density, twist, and tensile properties. Moreover, many previously reported yarn-forming strategies rely on a supporting core, which can limit access to fully nanofibrous architectures in applications where continuous nanoscale surface character is desirable [20].

To address these limitations, we developed a high-throughput AC electrospinning method for the continuous fabrication of 100% nanofiber yarns. The method does not require a supporting core, enables control over key yarn morphological features, and achieves production speeds approaching two orders of magnitude above those reported for representative conventional DC NFY-forming methods.

• **Comment 2.** A clear overview of AC electrospinning and nanofiber yarns is provided in introduction section; however, the manuscript does not explicitly articulate the research gap or clearly distinguish the novelty of the present work from prior studies. While AC electrospinning, high-throughput fiber production, and nanofiber yarn fabrication have all been reported previously, it remains unclear which specific limitations of existing approaches are addressed here. The authors are encouraged to explicitly define the unmet challenges in current AC electrospinning-based yarn fabrication.
Below are some of the relevant literature:

o Divyabharathi Madheswaran, Manikandan Sivan, Sarka Hauzerova, Eva Kuzelova Kostakova, Vera Jencova, Jan Valtera, Lubos Behalek, Jana Mullerova, Nhung H.A. Nguyen, Lukas Capek, David Lukas, Continuous fabrication of braided composite nanofibrous surgical yarns using advanced AC electrospinning and braiding technology, Composites Communications, Volume 48, 2024, https://doi.org/10.1016/j.coco.2024.101932.

o Mikule J, Friedrich O, Aguilera JP, Zabka P, Kostakova EK, Valtera J, Hauzerová Š, Skrivanek J, Jencova V, Batka O, Meszaros L. High throughput fabrication of nanofibrous yarns produced by AC electrospinning.

o Manikandan Sivan, Divyabharathi Madheswaran, Jan Valtera, Eva Kuzelova Kostakova, David Lukas, Alternating current electrospinning: The impacts of various high-voltage signal shapes and frequencies on the spinnability and productivity of polycaprolactone nanofibers, Materials & Design, Volume 213, 2022, https://doi.org/10.1016/j.matdes.2021.110308.

Response to Reviewers comment no. 2:

We thank the reviewer for this valuable comment.

Several of the suggested references are already included in the manuscript and cited in the relevant sections of the Introduction. In addition, the work by Divyabharathi Madheswaran et al. (2024) has now been incorporated into the corresponding paragraph.

Revised manuscript paragraph with incorporated cities:

Braiding of nanofibrous yarns. To demonstrate the processability of nanofibrous yarns into more complex textile architectures, we fabricated braided yarns, see Fig 8a. Braided structures are highly attractive for numerous advanced applications due to their combination of mechanical robustness, hierarchical pore organization, flexibility, and design versatility [46].

• **Comment 3.** In Section "Results" on page 3, line 93-97, the manuscript attributes plume propagation primarily to "electric wind." This statement would benefit from further clarification or supporting references, as plume dynamics in AC electrospinning are known to depend on multiple factors including field frequency, waveform, and solution properties.

Response to Reviewers comment no. 3:

We thank the reviewer for this valuable comment.

The cite no. 21, where "electric wind" has been experimentally investigated added.

Revised manuscript paragraphs with incorporated cite:

A distinctive feature of this configuration is the formation of a nanofibrous plume [21], resembling a thin, veil-like curtain of interconnected fibers (Fig. 1c). Driven by an electric wind, the plume propagates upward approximately along the plane of the rotating disc spinning-electrode.

• **Comment 4.** On page 5, line no 163, it is stated that torque is transmitted "only through the nanofiber mass itself, without physical contact with any external tool." This is an important and non-intuitive claim, yet it is presented qualitatively. The authors should clarify the physical basis of torque transfer within the nanofiber belt and discuss whether this mechanism has limits with respect to belt thickness or fiber density.

Response to Reviewers comment no. 4:

We thank the reviewer for highlighting this important and non-intuitive point.

The referenced sentence has now been substantially expanded to clarify the physical basis of torque transfer within the nanofibrous belt.

Revised manuscript paragraph:

However, the fluffy nanofibrous material accumulated on the rotating drum adheres strongly to any solid surface, which prevents direct mechanical engagement. As a result, torque is transmitted through the nanofibrous belt itself, without direct mechanical contact with any rigid tool. This transmission arises from the collective mechanical response of the densely entangled

nanofiber network, in which inter-fiber friction, van der Waals interactions, and geometric confinement within the spinning triangle—initially characterized by only a negligible shear moment—enable the propagation of shear stresses across the belt. The path extending along the spinning triangle, through the newly formed nanofibrous yarn and toward its anchoring in the twirling device, may be viewed as an axis of progressively increasing shear modulus, as the nanofibrous material becomes increasingly compact, denser, and richer in frictional and van der Waals contacts. The efficiency of this mechanism depends on the fiber density and belt thickness.

• **Comment 5.** Page 5, line 165, the assertion that the twisting torque simultaneously provides the tensile force required for detachment is interesting but insufficiently proven. The authors should clarify whether this coupling is intrinsic to the system geometry or dependent on specific process parameters.

Response to Reviewers comment no. 5:

We thank the reviewer for this insightful comment.

The corresponding passage has now been revised and expanded to clarify the nature of the coupling between twisting torque and tensile force.

Revised manuscript paragraph:

In our system, the tensile force required for peeling the nanofibrous belt from the drum surface is not applied independently, but arises as a direct consequence of the torque transmitted through the nanofibrous structure. This coupling is primarily determined by the rotational speed of the twirling device, and the geometry of the setup, particularly by the relative position of the drum collector, its surface, and the twirling device, which together define the shape of the spinning triangle and the direction of force transmission. In this sense, the coupling is intrinsic to the selected system geometry. A qualitative experimental indication of effective peeling is provided by the state of the drum surface after separation of the nanofibrous belt. In long-term spinning experiments, we do not observe progressive accumulation of residual nanofibrous material on the drum.

• **Comment 6.** Page 5 & 6, several mechanistic explanations rely on qualitative assumptions derived from conventional yarn mechanics. Incorporating quantitative parameters, clarifying optimization choices, and providing experimental evidence for the stabilizing role of the galette would significantly strengthen the rigor and reproducibility of this part of the manuscript.

Response to Reviewers comment no. 6:

We thank the reviewer for this constructive suggestion.

The manuscript has been revised to include an expanded explanation of the role of the galette in the system.

Revised manuscript paragraph:

The stability of both the yarn balloon and the spinning triangle is substantially enhanced by a rotating galette (Technical University of Liberec) positioned approximately 200 mm

downstream of the twirling device, where it securely grips the forming yarn (Supplementary Video 4). The galette defines a downstream boundary condition for the twisting process, acting as a tension stabilizer and twist brake. Without this stabilization, twist propagation toward the spinning triangle leads to dynamic instability. A mechanical tension sensor placed in front of the galette continuously monitors the yarn tension (Fig. 2d).

• **Comment 7.** Page 6, line 207, the proposed contributions of mechanical interlocking, van der Waals forces, and electrostatic attraction are described as assumptions rather than experimentally verified mechanisms. While reasonable, these assumptions should be explicitly framed as hypotheses or supported with references, measurements, or indirect evidence.

Response to Reviewers comment no. 7:

We thank the reviewer for this important remark.

The corresponding passage has been revised to more clearly reflect the speculative nature of the proposed mechanisms. We assume that the mechanical component of adhesion originates from the geometric interlocking of nanofiber segments with the surface of the substrate.

Revised manuscript paragraph:

In conventional ring-spinning systems, a flat ribbon of fibers—analogous to the nanofiber belt collected on our drum—passes through the front roller nip, where twist is imparted to the fibers by the rotating traveller or by a false-twist device 37. In such systems, the spinning triangle has a well-defined width and a base fixed by the roller nip. In our setup, the rotating drum collector provides an analogous function through frictional and adhesive interactions with the nanofibres, entirely in the absence of mechanical nipping. These interactions furnish a stable base for the nanofiber belt, maintaining a geometrically controlled and dynamically stable spinning triangle during continuous yarn formation.

To elucidate the stability of the spinning triangle in our configuration, we propose that its base is anchored to the drum collector substrate through a combination of mechanical interlocking, van der Waals interactions, and electrostatic attraction acting in concert. At present, these contributions cannot be directly resolved experimentally and are therefore formulated as physically motivated hypotheses.

• **Comment 8.** The authors provide a list of process parameters; however, the manuscript does not explain how these parameters were selected or optimized. Please clarify whether they were chosen based on prior literature, preliminary experiments, or systematic optimization, and discuss how sensitive the process is to variations in these parameters.

Response to Reviewers comment no. 8:

We thank the reviewer for this helpful comment.

The “AC electrospinning apparatus“ section in Methods has been expanded to clarify how the process parameters were selected.

Additional Methods paragraph:

The process parameters used in this study were selected based on a combination of prior experience with AC electrospinning systems and systematic preliminary experiments aimed at identifying a stable operating regime for continuous yarn formation (tab. 1 and tab. 2). Electrical parameters (voltage amplitude, frequency, and electrode–collector distance) were initially chosen according to established AC electrospinning conditions and subsequently refined to ensure stable plume formation. Mechanical parameters governing yarn formation, such as the rotational speed of the twirling device, drum surface velocity, and take-up speed, were adjusted iteratively to achieve a stable spinning triangle and continuous yarn production. The process operates within a stability window defined by the balance between nanofiber production rate, adhesion to the collector, and torque-driven yarn formation. Within this window, moderate variations in parameters do not significantly affect process stability.

• **Comment 9.** Page 9, the use of principal rose vectors is appropriate for visualizing orientation trends; however, the manuscript does not clearly define how these vectors are calculated or normalized. A brief methodological description or reference would improve reproducibility and clarity.

Response to Reviewers comment no. 9:

We thank the reviewer for this useful comment.

The Methods section, specifically the "Scanning electron microscopy investigation" subsection, has been expanded to clearly describe the calculation and normalization of the principal rose vectors.

Additional Methods paragraph:

Fiber anisotropy and orientation were further analyzed in ImageJ using the DiameterJ and OrientationJ plugins 50, enabling automated quantification of fiber alignment distributions within the nanofibrous yarns.The local orientation of nanofiber segments was computed from the coresponding binarized SEM micrograph and expressed as an angular distribution in the range 0–180°, accounting for the axial symmetry of fiber orientation.The orientation data were represented as directional histograms ("roses of directions"), where the frequency of fiber segments falling within each angular bin was evaluated. The principal rose vector was defined as the vector corresponding to the dominant orientation, i.e., the angular bin with the maximum frequency. The direction of the vector is given by this dominant angle, while its magnitude is proportional to the normalized maximum frequency. Normalization was performed with respect to the total number of detected fiber segments in the analyzed region, allowing direct comparison between different spatial locations within the spinning triangle. This representation provides a compact visualization of both the preferred orientation and the degree of alignment of the nanofibrous structure.

• **Comment 10.** Page 12, section "**Fiber density and pore size distribution.",** the probability density function used to derive the pore radius implicitly assumes a spatially random and isotropic distribution of fibers. Given that the manuscript later demonstrates pronounced fiber alignment and anisotropy within the yarn, the validity of applying an isotropic pore model should be critically addressed.

Response to Reviewers comment no. 10:

We thank the reviewer for raising this important issue.

In the revised manuscript, we have explicitly addressed this limitation. The pore-size analysis is now clearly restricted to cross-sections perpendicular to the yarn axis. At the same time, we acknowledge that the nanofibrous yarn exhibits pronounced anisotropy, as demonstrated by the spinning triangle analysis.

To further strengthen the analysis beyond the assumption of complete spatial randomness (Poisson point process), we complemented the analytical model with Monte Carlo simulations based directly on the experimentally extracted point patterns. This approach allows us to partially account for deviations from ideal randomness, as confirmed by the stereological analysis presented in the Supplementary (Ripley K-function, $L(r) - r$, and pair correlation g-function).

The results show that although local deviations from Poisson behaviour are present, the most probable pore radius obtained from the Poisson model (1.25 μm) and Monte Carlo simulations (1.13 μm) are in close agreement, indicating that the Poisson process approximation provides a reasonable first-order description of pore geometry in the analysed cross-sections.

Revised paragraph:

A perpendicular cross-section of a PVB nanofibrous yarn obtained using a Tescan Vega SEM is shown in Fig. 6a,b. In total, n = 1270 fiber endpoints, including merged structures typical of AC-type nanofibrous systems, were identified within a cumulative analyzed area of 11 995 μm², across four images, corresponding to a fiber number density N_S = 0.106 μm-2. Based on this value, the probability density function *f(r)* of the pore radius was calculated (Fig. 6c), yielding a most probable pore radius of r_p = 1.25 μm. However, analysis of the extracted point processes revealed deviations from an ideal Poisson distribution; therefore, complementary Monte Carlo simulations based on the real data were performed, yielding a most probable pore radius of r_p = 1.13 μm. A detailed stereological analysis of the point processes for individual images is provided in the Supplementary. It should be noted that the nanofibrous yarn exhibits pronounced anisotropy, as demonstrated by the analysis of the spinning triangle. Consequently, the applied pore model does not account for anisotropic effects that may arise in other orientations of the fibrous structure. A rigorous stereological description of such an anisotropic system would require isotropic sections; however, these are not experimentally accessible in the present case. Non-perpendicular sections tend to disintegrate without reinforcement, while resin embedding introduces capillary-induced artefacts that can significantly alter the fiber distribution. For these reasons, the analysis was restricted to cross-sections perpendicular to the yarn axis, and the results should be interpreted within this limitation. Despite this constraint, such sections remain highly relevant for applications where transport occurs along the yarn axis—such as liquid imbibition described by the Lucas–Washburn relation [44]—as well as for filtration systems based on nanofibrous yarns, where particle penetration predominantly occurs perpendicular to the yarn axis.

• **Comment 11.** The pore size calculation is based on a single SEM cross-section containing 279 fibers within a reference area of 761.76 μm². It is unclear whether this region is representative of the entire yarn cross-section or whether multiple locations and yarns were analyzed. The authors should clarify the number of samples and sections evaluated to ensure statistical robustness.

Response to Reviewers comment no. 11:

We thank the reviewer for this important comment regarding statistical robustness.

The analysis has now been refined and extended. Specifically, the pore size evaluation was performed using four independent SEM cross-sectional images rather than a single region. Furthermore, a more detailed stereological analysis has been carried out, and the full methodology and results have been included in the Supplementary Information (Section S3). These additions improve the representativeness and reliability of the reported values.

Revised paragraph:

A perpendicular cross-section of a PVB nanofibrous yarn obtained using a Tescan Vega SEM is shown in Fig. 6a,b. In total, n = 1270 fiber endpoints, including merged structures typical of AC-type nanofibrous systems, were identified within a cumulative analyzed area of 11 995 μm^2, across four images, corresponding to a fiber number density $N_S = 0.106\ \mu m^{-2}$. Based on this value, the probability density function *f(r)* of the pore radius was calculated (Fig. 6c), yielding a most probable pore radius of $r_P = 1.25\ \mu m$. However, analysis of the extracted point processes revealed deviations from an ideal Poisson distribution; therefore, complementary Monte Carlo simulations based on the real data were performed, yielding a most probable pore radius of $r_P = 1.13\ \mu m$. A detailed stereological analysis of the point processes for individual images is provided in the Supplementary. It should be noted that the nanofibrous yarn exhibits pronounced anisotropy, as demonstrated by the analysis of the spinning triangle. Consequently, the applied pore model does not account for anisotropic effects that may arise in other orientations of the fibrous structure. A rigorous stereological description of such an anisotropic system would require isotropic sections; however, these are not experimentally accessible in the present case. Non-perpendicular sections tend to disintegrate without reinforcement, while resin embedding introduces capillary-induced artefacts that can significantly alter the fiber distribution. For these reasons, the analysis was restricted to cross-sections perpendicular to the yarn axis, and the results should be interpreted within this limitation. Despite this constraint, such sections remain highly relevant for applications where transport occurs along the yarn axis—such as liquid imbibition described by the Lucas–Washburn relation [44]—as well as for filtration systems based on nanofibrous yarns, where particle penetration predominantly occurs perpendicular to the yarn axis.

• **Comment 12.** The manuscript transitions from internal pore statistics to surface fiber curvature and torsion without explicitly justifying how surface measurements reflect internal yarn structure. Given that yarn compaction and stress states may vary radially, this assumption should be stated and discussed more explicitly.

Response to Reviewers comment no. 12:

We thank the reviewer for this insightful comment.

A new paragraph in "Curvature and torsion of surface nanofibers" subsection has been added to explicitly address this point.

New manuscript paragraph:

The estimation of curvature and torsion presented here is based on measurements of nanofibers located at the yarn surface. This approach implicitly assumes that the outer layers are representative, at least qualitatively, of the internal fibrous structure. However, it should be noted that radial variations in fiber packing density, stress state, and local rearrangement may occur between the yarn surface and its core. The reported curvature and torsion values should therefore be interpreted primarily as descriptors of the surface and near-surface structure. A fully resolved characterization of the internal three-dimensional fiber arrangement would require advanced volumetric imaging techniques, which are beyond the scope of the present study.

• **Comment 13.** Caption of Figure 9, "AC electrospinning setup and imaging arrangement" does not describe the Figure 9(d) in its title.

Response to Reviewers comment no. 13:

We thank the reviewer for noting this inconsistency.

The caption of Figure 9 has been corrected.

Revised figure caption:

Fig. 9 | AC electrospinning setup, spinning triangle imaging arrangement and braidng machine. *(a) Voltage waveforms for AC electrospinning generated by the TREK 50/12 high-voltage amplifier. Waveform shapes illustrating a sinusoidal (red) and a step-function (blue) input signal, both at 50 Hz. (b) Schematic illustration of the spinning electrode and polymer solution circulation system: (A) liquid film of the polymer solution on the disc surface; (B) passage of the disc through the wiping slit; (C) overflow of the polymer solution; (D) recirculation of part of the solution onto the disc edge; (E) supply of the polymer solution from an external reservoir; and (F) closed-loop return of the solution driven by a gear pump. (c) Schematic of the imaging setup for observing the spinning triangle and yarn formation: (A) camera for twist-triangle observation; (B) illumination source; (C) twisting device; (D) spinningt triangle; and (E) ballooning yarn. (d) Braiding machine EY-RA-8-4 (110 II) equipped with an eight-carrier arrangement, used for fabricating braided nanofiber yarns. The original ceramic eyelet guides were replaced with pulleys to minimize friction.*

• **Comment 14.** On page 18, in section "Cell proliferation assay " the comparison between woven nanofibrous yarn scaffolds and planar DC-electrospun mats is interesting; however, the rationale for selecting only PCL for the biological evaluation is not clearly justified. The authors should clarify whether PCL was chosen as a representative system or whether similar trends are expected for other polymers used in the study.

Response to Reviewers comment no. 14:

We thank the reviewer for this important comment.

The corresponding section has been revised to clarify the rationale for selecting PCL for the biological evaluation.

Revised paragraph:

Cell proliferation assay. Poly(ε-caprolactone) (PCL) was selected as a model polymer for biological evaluation due to its well-established biocompatibility and widespread use in tissue engineering. The purpose of the experiment was to assess the effect of scaffold architecture—woven nanofibrous yarns versus planar electrospun layers—on cell behaviour, rather than to compare different polymer chemistries. By maintaining a constant material composition, the influence of structural organization on cell adhesion and proliferation could be isolated. The interaction of 3T3 mouse fibroblasts with the tested materials was evaluated on two types of PCL substrates: (i) woven scaffolds fabricated entirely from nanofibrous yarns, and (ii) planar nonwoven nanofibrous layers produced by direct-current (DC) electrospinning of the same polymer.

• **Comment 15.** On page 18, in section "Cell proliferation assay ", the planar nanofibrous mat is described as consisting of both nano- and microfibers with a very broad diameter distribution (700 ± 830 nm). Such heterogeneity may significantly influence cell behavior. The authors should discuss how this structural variability affects the comparison with the woven scaffold and whether fiber diameter was intentionally controlled.

Response to Reviewers comment no. 15:

We thank the reviewer for this important observation.

The corresponding section has been revised to address the effect of fiber diameter heterogeneity.

Revised paragraph:

The planar nanofibrous mat consisted of nano- and microfibers with an average diameter of 700 ± 830 nm, reflecting the broad diameter distribution arising from the selected solvent system. A comparable structural heterogeneity was also observed in the nanofibrous yarns, which consisted predominantly of micron-scale fibers with a similarly wide diameter distribution (Fig. 4). Therefore, the comparison between planar and woven scaffolds primarily reflects differences in structural organization rather than fiber size distribution. Initially, cell adhesion was markedly lower on the woven scaffolds than on both the planar nanofibrous layers and the tissue-culture plastic control (Fig. 8d).

• **Comment 16.** The conclusion section is very brief and does not sufficiently summarize the key findings of the study. In particular, it lacks a clear synthesis of the main quantitative results and performance metrics achieved in this work. The manuscript would benefit from a more elaborate conclusion presented as a dedicated section, highlighting the most significant outcomes, numerical values, and their implications for future applications.

Response to Reviewers comment no. 16:

We thank the reviewer for this valuable suggestion.

The Conclusion section has been revised and substantially expanded. It now provides a clearer synthesis of the key findings, including the main quantitative results and performance metrics achieved in this study, as well as their broader implications for future applications.

Revised conclusion:

Microstructural analyses reveal deformation mechanisms distinct from conventional multifilaments. Moderately coiled helical nanofibers on yarn surfaces straighten during early tensile loading, explaining the hierarchy of mechanical behaviour and providing a basis for systematic tuning. Post-processing further expands this design space; for instance, converting smooth PCL fibres to shish–kebab morphologies markedly increases surface area and offers routes to modulating adhesion, mechanics, and functionalization.

Morphological analysis revealed that the produced yarns consist of densely packed nanofibers with characteristic diameters of approximately 220 ± 60 nm (PA46), 410 ± 150 nm (PVA), 870 ± 250 nm (PVB), and 1140 ± 490 nm (PCL), with the latter showing a broader distribution extending into the microscale. Cross-sectional analysis of PVB yarn yielded a fiber number density of NS ≈ 0.106 μm^{-2} and a most probable pore radius of rp ≈ 1.25 µm, confirming a fine and interconnected porous architecture.

The internal structural organization of the yarn was further characterized through stereological analysis of surface nanofibers, yielding an average curvature of ~0.10 μm^{-1} and torsion of ~0.032 μm^{-1}. These values correspond to a moderately coiled helical geometry of the fibers, which plays a key role in the mechanical response of the yarn, particularly during the initial deformation stage dominated by fiber straightening.

Mechanical testing demonstrated a wide tunability of properties depending on polymer type, ranging from high-strength, low-elongation behaviour in PA46 (≈55 MPa tensile strength) to highly extensible responses in PCL (elongation up to ≈150%). This variability highlights the flexibility of the approach in tailoring yarn performance for specific applications.

Finally, the performance of 3D woven NFY scaffolds demonstrates the tissue engineering potential of this platform. Although these constructs showed delayed early adhesion due to initial cell settlement through the pores, proliferation progressed rapidly thereafter and reached confluence by day 14, comparable to planar controls. This decoupling of early occupancy from later 3D colonization underscores the unique architectural control available through yarn fineness, twist, and textile topology.

Reviewer #2 (Remarks to the Author):

I co-reviewed this manuscript with one of the reviewers who provided the listed reports. This is part of the Communications Engineering initiative to facilitate training in peer review and to provide appropriate recognition for Early Career Researchers who co-review manuscripts.

Reviewer #3 (Remarks to the Author):

This manuscript presents an interesting and well-executed study on the continuous fabrication of core-free nanofibrous yarns using AC electrospinning. The topic is relevant, the processing concept is technically strong, and the work addresses an important challenge in the field: the transition from nanofiber production to more practical, textile-compatible 1D structures.

The study is clearly beyond a simple proof of concept. The authors demonstrate yarn formation for several polymers, provide morphological and mechanical characterization, and further show the possibility of braiding, weaving, and preliminary biological evaluation. Overall, I find the manuscript valuable and suitable for publication after minor revision.

My main comment concerns the way scalability is currently presented. The reported production rates are promising and clearly indicate strong scale-up potential. At the same time, the current manuscript would benefit from slightly more careful wording in this section, as the experimental evidence remains based on relatively limited operating time and a restricted set of processing conditions.

Aside from this point, the manuscript is well structured, and the results are presented clearly. I do not see any major scientific weakness that would require substantial additional experiments. The suggested revision is intended primarily to improve the precision of the results' interpretation.

In summary, this is a strong manuscript with clear practical relevance for the nanofiber and textile engineering communities. I recommend publication after minor revision.

To further strengthen the manuscript, I recommend that the authors address the scalability claim in a more precise, quantitative way. This could be done through a few relatively small additions:

• **Comment 17/** Add a brief comment on reproducibility.
Even a brief statement indicating whether the reported yarn production was reproduced in independent runs would strengthen the work's engineering relevance. If available, the authors could report the number of replicate experiments and whether similar yarn quality and process stability were consistently achieved.

Response to Reviewers comment no. 17:

We thank the reviewer for this helpful suggestion.

The corresponding paragraph has been revised, and the requested information regarding reproducibility has been added.

Revised paragraph:

Productivity. The production performance of nanofibrous yarns prepared from PVB, PA46, PCL, and PVA polymers is summarized in Table 1. The listed parameters include the linear and mass production rates, as well as the polymer solution consumption rate, which directly reflects the conversion efficiency into solid nanofibrous material. A key metric for scaling this technology to an industrial level is the production speed, which ranged from 0.85 to 9.4 m min^{-1}, accompanied by relatively high linear densities of 30.4–36.4 tex. These processing conditions were selected to obtain yarns within the targeted fineness range of 30–40 tex and correspond to routine stable operating conditions for continuous yarn formation across all investigated materials. However, the process allows for straightforward adjustment of the linear density across a broader range; in particular, coarser yarns up to ~100 tex can be readily produced by reducing the winding speed, along with appropriate adjustments of the drum rotation speed and the twirling device rotation speed, while maintaining the desired yarn quality. A remarkable feature of our process is its operational stability, enabling continuous yarn formation for at least one hour without interruption, as evidenced by a 300 m long wound spool (Fig. 2e). This stability was confirmed for each material by repeated experiments (typically 10–50 runs), consistently yielding yarns of comparable quality and resulting in the cumulative production of several hundreds of meters to kilometers of yarn. In general, higher linear densities were associated with increased process stability, as the larger amount of fibrous material in the yarn reduces the likelihood that local inhomogeneities arising during the spinning process lead to yarn breakage, whereas at fineness below of 20 tex the stability gradually decreases. Furthermore, a dedicated experiment with PVB nanofibrous yarn demonstrated a maximum achievable production speed of 55 m min^{-1}, corresponding to a yarn fineness of 5 tex (Fig. 2f). Beyond this threshold, the process became unstable, and yarn breakages were observed in the region of the twirling device.

• **Comment 18**/Clarify the distinction between routine operating speed and maximum demonstrated speed.

In the current version, the manuscript reports routine production rates for different polymers and also mentions a maximum speed of 55 m min^{-1} for PVB. It would help the reader if the authors explicitly separate these two cases and indicate more clearly which conditions correspond to stable standard operation and which represent the upper performance limit of the setup.

Response to Reviewers comment no. 18:

We thank the reviewer for this helpful comment.

The manuscript has been revised to more clearly distinguish between routine operating speeds and the maximum demonstrated speed.

Revised paragraph:

Productivity. The production performance of nanofibrous yarns prepared from PVB, PA46, PCL, and PVA polymers is summarized in Table 1. The listed parameters include the linear and mass production rates, as well as the polymer solution consumption rate, which directly reflects the conversion efficiency into solid nanofibrous material. A key metric for scaling this

technology to an industrial level is the production speed, which ranged from 0.85 to 9.4 m min^{-1}, accompanied by relatively high linear densities of 30.4–36.4 tex. These processing conditions were selected to obtain yarns within the targeted fineness range of 30–40 tex and correspond to routine stable operating conditions for continuous yarn formation across all investigated materials. However, the process allows for straightforward adjustment of the linear density across a broader range; in particular, coarser yarns up to ~100 tex can be readily produced by reducing the winding speed, along with appropriate adjustments of the drum rotation speed and the twirling device rotation speed, while maintaining the desired yarn quality. A remarkable feature of our process is its operational stability, enabling continuous yarn formation for at least one hour without interruption, as evidenced by a 300 m long wound spool (Fig. 2e). This stability was confirmed for each material by repeated experiments (typically 10–50 runs), consistently yielding yarns of comparable quality and resulting in the cumulative production of several hundreds of meters to kilometers of yarn. In general, higher linear densities were associated with increased process stability, as the larger amount of fibrous material in the yarn reduces the likelihood that local inhomogeneities arising during the spinning process lead to yarn breakage, whereas at fineness below of 20 tex the stability gradually decreases. Furthermore, a dedicated experiment with PVB nanofibrous yarn demonstrated a maximum achievable production speed of 55 m min^{-1}, corresponding to a yarn fineness of 5 tex (Fig. 2f). Beyond this threshold, the process became unstable, and yarn breakages were observed in the region of the twirling device.

• **Comment 19**/ Provide a short note on continuous-operation stability.

The manuscript already states that continuous production for at least one hour was achieved and shows a spool as evidence. It would be useful to expand this slightly by indicating whether this stability was observed only under selected conditions or more generally across the tested polymer systems (e.g., only for 1 polymer, just for show).

Response to Reviewers comment no. 19:

We thank the reviewer for this valuable suggestion.

The corresponding passage has been expanded to clarify the conditions under which continuous-operation stability was achieved.

Revised paragraph:

Productivity. The production performance of nanofibrous yarns prepared from PVB, PA46, PCL, and PVA polymers is summarized in Table 1. The listed parameters include the linear and mass production rates, as well as the polymer solution consumption rate, which directly reflects the conversion efficiency into solid nanofibrous material. A key metric for scaling this technology to an industrial level is the production speed, which ranged from 0.85 to 9.4 m min^{-1}, accompanied by relatively high linear densities of 30.4–36.4 tex. These processing conditions were selected to obtain yarns within the targeted fineness range of 30–40 tex and correspond to routine stable operating conditions for continuous yarn formation across all investigated materials. However, the process allows for straightforward adjustment of the linear density across a broader range; in particular, coarser yarns up to ~100 tex can be readily produced by reducing the winding speed, along with appropriate adjustments of the drum

rotation speed and the twirling device rotation speed, while maintaining the desired yarn quality. A remarkable feature of our process is its operational stability, enabling continuous yarn formation for at least one hour without interruption, as evidenced by a 300 m long wound spool (Fig. 2e). This stability was confirmed for each material by repeated experiments (typically 10–50 runs), consistently yielding yarns of comparable quality and resulting in the cumulative production of several hundreds of meters to kilometers of yarn. In general, higher linear densities were associated with increased process stability, as the larger amount of fibrous material in the yarn reduces the likelihood that local inhomogeneities arising during the spinning process lead to yarn breakage, whereas at fineness below of 20 tex the stability gradually decreases. Furthermore, a dedicated experiment with PVB nanofibrous yarn demonstrated a maximum achievable production speed of 55 m min^{-1}, corresponding to a yarn fineness of 5 tex (Fig. 2f). Beyond this threshold, the process became unstable, and yarn breakages were observed in the region of the twirling device.

• **Comment 20**/. Slightly moderate the wording where appropriate. If additional quantitative data are not available, I would suggest using slightly more cautious phrasing, such as “shows strong scale-up potential” or “represents a promising route toward scalable production,” rather than making broader claims of scalability or industrial readiness at this stage. The current data clearly support the method's promise, but it has not yet undergone full engineering validation at an industrial level.

Response to Reviewers comment no. 20:

We thank the reviewer for this thoughtful recommendation.

The wording throughout the manuscript has been carefully revised to adopt a more cautious and balanced tone.

Response to Reviewer #1, Comment 1

We agree that the research gap and the novelty of the present work must be stated in a more explicit and focused manner. In the firstly revised manuscript, we clarified several limitations of previously reported nanofiber-yarn fabrication approaches in the Introduction, page 2. However, in response to the Reviewer's further comment, we now recognize that these points should be brought together into a stronger and more direct statement of the unresolved gap and the specific contribution of the present study.

The firstly revised Introduction already identified three important limitations in the prior literature.

First, many DC-based nanofiber-yarn fabrication strategies remain limited by relatively low solution feed rates and modest collection speeds, with representative yarn take-up speeds often below 2 m min^{-1} and reaching approximately 10 m min^{-1} only in post-drawing-assisted configurations.

Second, nanofiber-yarn production remains limited by insufficient control over yarn diameter, linear density, twist, and tensile properties.

Third, many previously reported yarn-forming approaches rely on a supporting core, which restricts access to fully nanofibrous architectures in applications where a continuous nanoscale surface and fully nanofibrous bulk structure are desirable. These limitations are now explicitly discussed in the revised Introduction.

In addition, the revised manuscript distinguishes the present study from earlier AC-electrospinning-based yarn concepts. Previous AC electrospinning work demonstrated the formation of nanofibrous plumes and, importantly, composite nanofiber yarns produced by wrapping an AC-generated plume around a core thread. While this represented a significant advance, it did not fully solve the problem of producing continuous, mechanically processable, core-free, 100% nanofibrous yarns at high throughput. The remaining technological gap was therefore not merely the use of AC electrospinning itself, but the conversion of an AC-generated nanofibrous plume into a continuous yarn composed entirely of nanofibers, with controllable morphology and production speeds relevant to textile-scale processing.

To make this distinction clearer, we have further refined the Introduction in the secondly revised manuscript.

Text in the firstly revised manuscript Introduction:

To address these limitations, we developed a high-throughput AC electrospinning method for the continuous fabrication of 100% nanofiber yarns. The method does not require a supporting core, enables control over key yarn morphological features, and achieves production speeds approaching two orders of magnitude above those reported for representative conventional DC NFY-forming methods.

The revised statement in Introduction now reads:

"Despite the advances, a major unresolved challenge remains: the continuous high-throughput fabrication of mechanically processable, core-free nanofiber yarns composed entirely of

electrospun nanofibers. Previously reported DC-based NFY fabrication strategies are constrained by relatively low solution feed rates and modest collection speeds, with representative yarn take-up speeds often below 2 m min^{-1} and reaching about 10 m min^{-1} only in post-drawing-assisted configurations [32,33]. These limitations arise in part from the electrostatic nature of DC electrospinning, where emitted polymer jets and deposited fibers typically carry charges of the same polarity and undergo Coulombic repulsion, promoting jet divergence and spatial dispersion of the forming fibers. This makes it difficult to generate a compact, mechanically coherent nanofibrous plume suitable for direct transformation into continuous linear structures. AC electrospinning provides a route to overcome these limitations through periodic charge reversal and self-neutralization, producing a compact, electrically neutral nanofibrous plume and enabling substantially higher nanofiber production rates. However, previously reported AC-electrospinning-based NFYs have remained core–shell composite structures, in which an AC-generated nanofibrous sheath is formed around a supporting core thread. Although this core provides the principal macroscale continuity and mechanical integrity, its presence may limit applications requiring fully nanofibrous, core-free yarn architectures [32].

To address this gap, we developed a needleless, collectorless AC electrospinning strategy that directly transforms an electrically neutral nanofibrous plume into continuous 100% nanofiber yarns through a controlled spinning triangle and false-twist/twirling mechanism. This approach eliminates the need for a supporting core, enables control over yarn morphology, linear density and twist-related structure, and achieves production speeds approaching two orders of magnitude above those reported for representative conventional DC NFY-forming methods."

We believe that this revised formulation now more clearly identifies the remaining limitations of previous nanofiber-yarn fabrication approaches and defines the unique scientific and technological advancement of the present study: the direct, high-throughput, continuous production of core-free 100% nanofiber yarns by AC electrospinning, combined with controllable yarn formation through a spinning-triangle and false-twist/twirling mechanism.

Review comments of Revised Version of Manuscript

Overall, the revised manuscript clearly improves the manuscript in terms of scientific rigor, scope clarification, and reproducibility. However, the revised version of manuscript does not fully address the main concern regarding the explicit identification of the research gap and novelty of the present work. While additional references were incorporated, the revised text still lacks a clear statement explaining:

- what specific limitations remain in previously reported AC electrospinning-based nanofiber yarn fabrication approaches,
- and what unique scientific or technological advancement is introduced in the present study.
