# Supplementary material for: High-throughput fabrication of nanofibrous yarns produced by alternating-current electrospinning
Source: Commun Eng. 2026 Jul 22;5:196. doi: 10.1038/s44172-026-00737-x (PMC13759490; doi:10.1038/s44172-026-00737-x)
Supplement: Supplementary file 2 [file 44172_2026_737_MOESM2_ESM.pdf]

Supplementary information

High throughput fabrication of nanofibrous yarns produced by alternating-current electrospinning

Jaroslav Mikule[1,3]*, Ondrej Friedrich[2], Juan Pablo Perez Aguilera[1,3], Petr Zabka[2], Eva Kuzelova Kostakova[1], Jan Valtera[2], Sarka Hauzerova[1], Josef Skrivanek[2], Vera Jencova[1], Ondrej Batka[2], Meszaros Laszlo[4], Jan Milucky[2], Manikandan Sivan[1], Jaroslav Beran[2] and David Lukas[1]*.

[1]*Technical University of Liberec, Faculty of Science, Humanities and Education, Department of Chemistry, Liberec, Czech Republic, EU*
[2]*Technical University of Liberec, Faculty of Mechanical Engineering, Department of Textile Machine Design, Liberec, Czech Republic, EU*
[3]*Technical University of Liberec, Faculty of Mechatronics, Informatics and Interdisciplinary studies, Liberec, Czech Republic, EU*
[4]*Budapest University of Technology and Economics, Faculty of Mechanical Engineering, Department of Polymer Engineering, Budapest, Hungary, EU*

Contents of Supplementary Information

S1 The planar anisotropy analysis of spinning triangle ………………………. 1
S2 The non-uniform twist distribution ……………………………………... 5
S3 Stereological Analysis of Pore Size……………………………………... 6
S4 Measurement of the average curvature and torsion ………………………. 11

S1. The planar anisotropy analysis of spinning triangle

To examine the orientation of individual nanofibers within the spinning triangle, a sample was collected by stopping the production of the PA46 nanofiber yarn and transferring the intact spinning triangle onto a sheet of paper without deforming its fibres. The paper with attached nanofibers was then cut into 25 × 25 mm square specimens to facilitate precise localization of partial samples for the subsequent plane-anisotropy analysis performed on SEM images.

The samples taken from the spinning triangle were identified according to the scheme shown in Fig. S1.1. The triangle is divided into square regions (the grid) arranged in rows labelled a–d and columns labelled 1–5. Within each square region, individual subsamples are assigned an internal index ranging from 1 to a maximum of 5. For example, the specimen labelled 4C_3 corresponds to the square located at column 4 and row C, with 3 denoting the internal position of the sample within that square.

Each SEM-examined subsample was assigned a corresponding orientation-rose plot using the same identification code. The orientation roses describe the distribution of nanofiber-segment directions within the analysed region. These rose plots were subsequently used to construct a vector field graphically representing the spatial distribution of dominant nanofiber orientations across the spinning triangle.

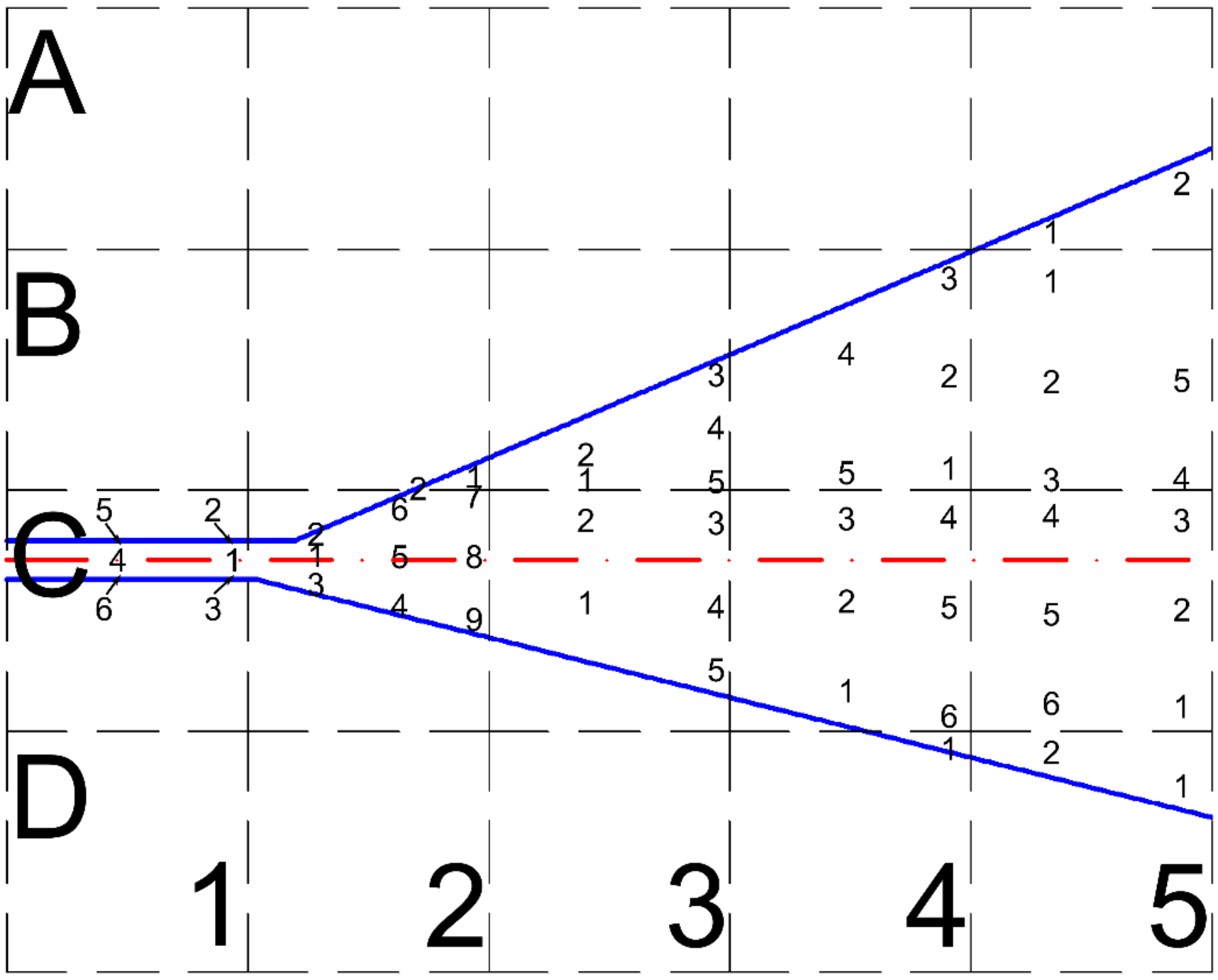

Fig. S1.1. *Schematic illustration of the sampling regions used for fibre-orientation analysis within the spinning triangle. Orientation-rose plots in Fig. S1.2 follow the notation 4C_3, where the letter–number pair defines the square region and the final digit indicates the internal position.*

The complete set of orientation-rose plots used for the analysis of in-plane anisotropy across the spinning triangle is shown in Fig. S1.2.

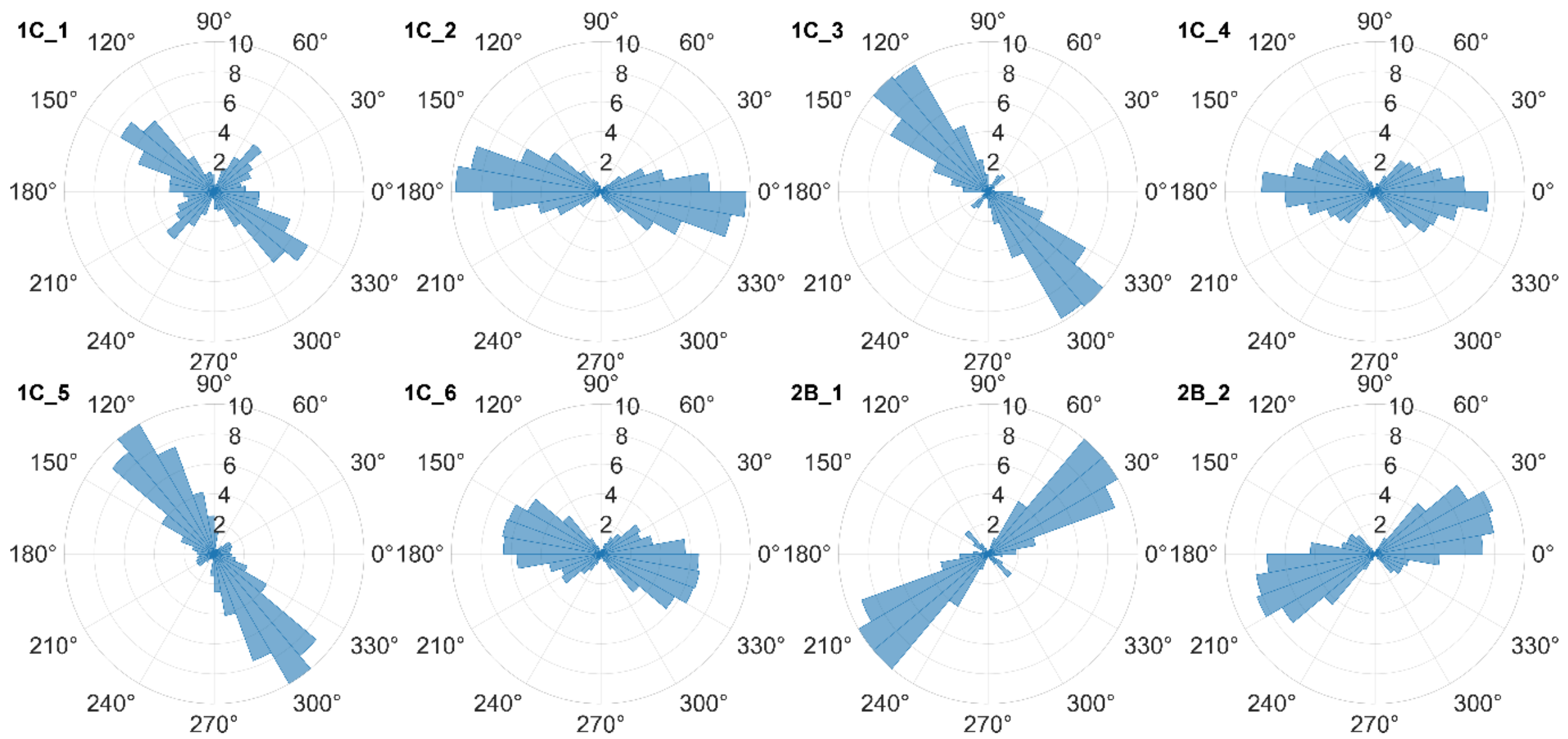

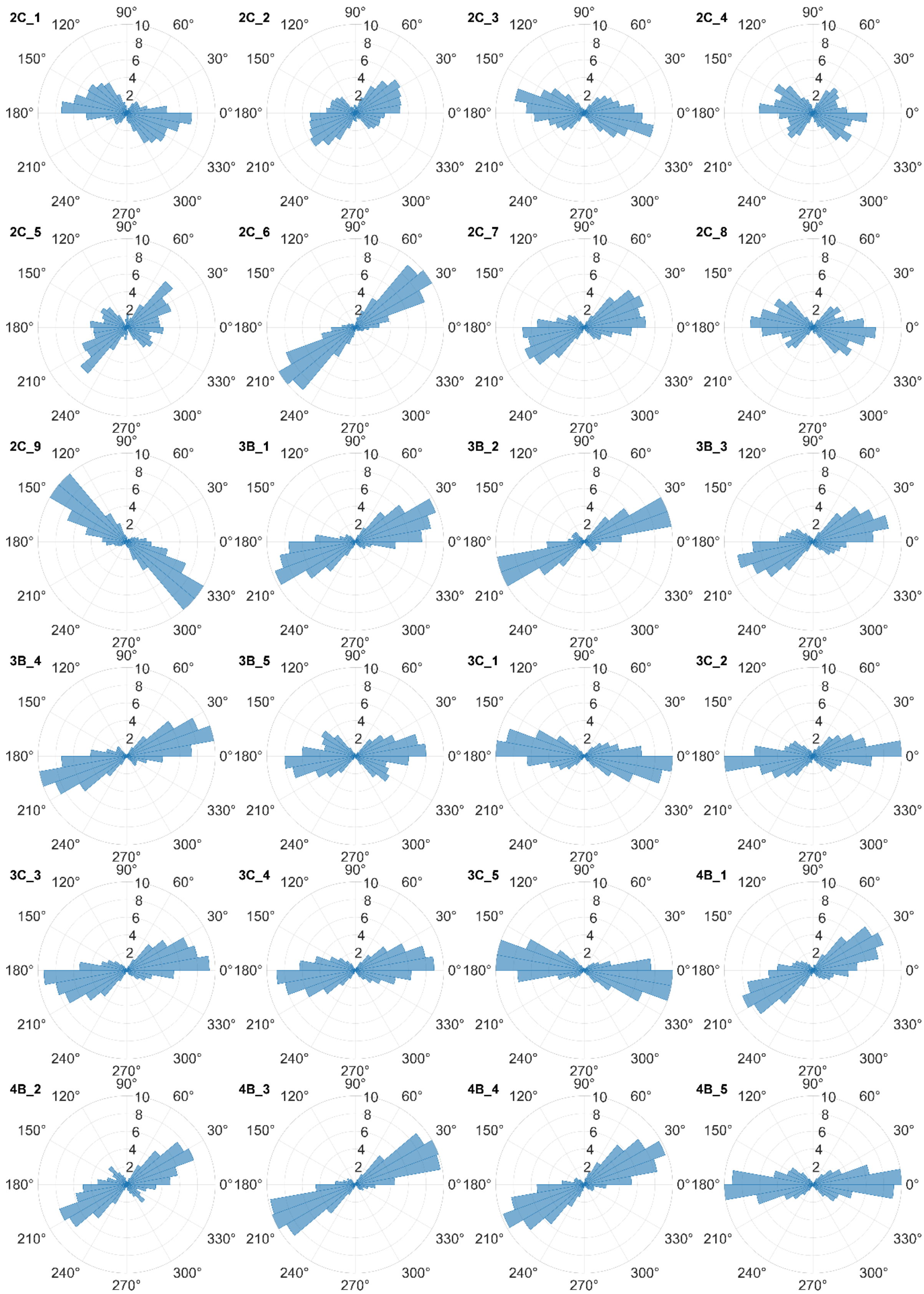
2C_1
2C_2
2C_3
2C_4
2C_5
2C_6
2C_7
2C_8
2C_9
3B_1
3B_2
3B_3
3B_4
3B_5
3C_1
3C_2
3C_3
3C_4
3C_5
4B_1
4B_2
4B_3
4B_4
4B_5

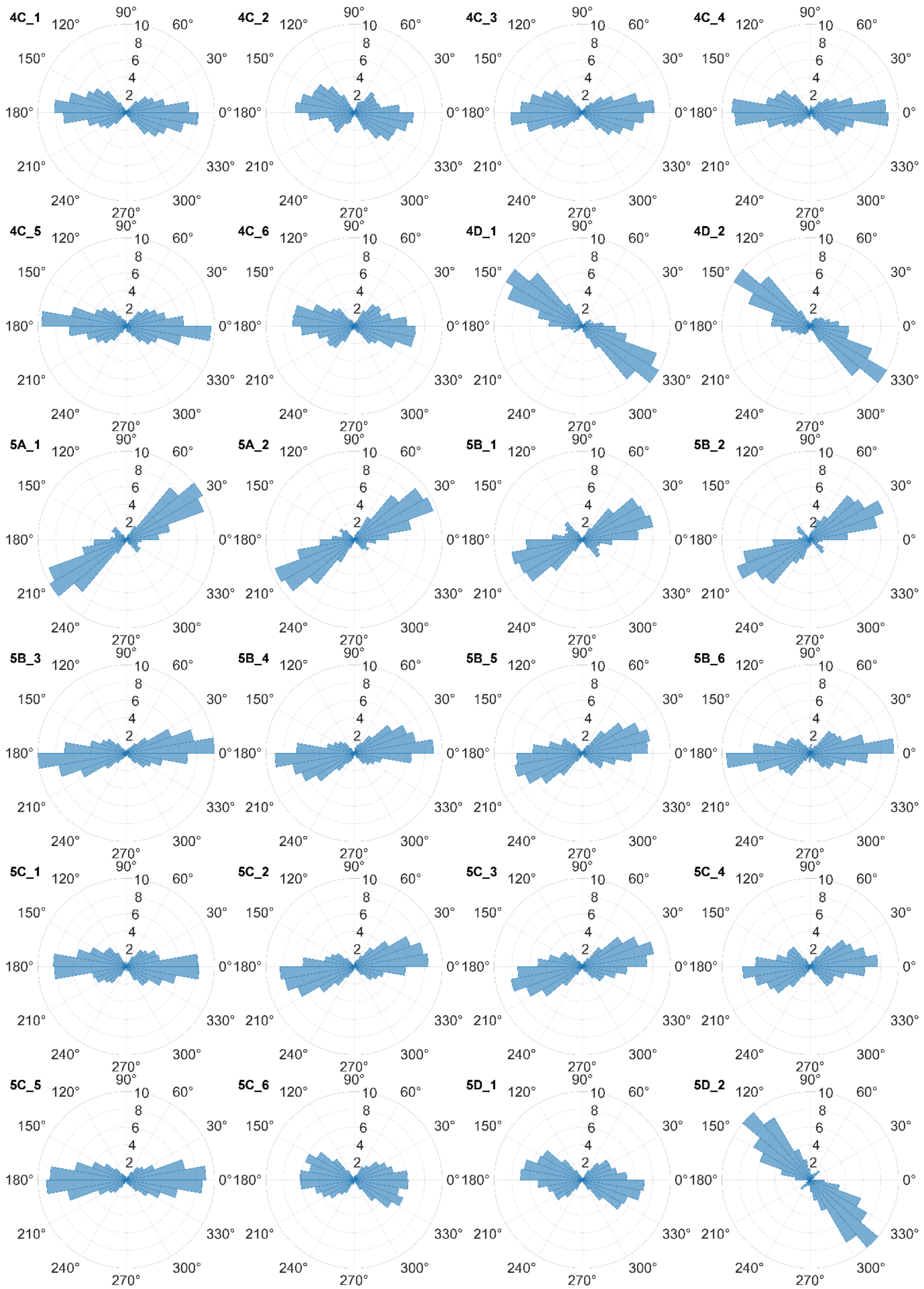

Fig. S1.2. *Orientation-rose plots for all sampling regions within the spinning triangle. Each region is identified by a code of the form* 1A_1*. The first number (1–5) and letter (A–D) designate the sampling square within the grid, while the final digit indicates the internal subsample position. The spatial arrangement of all labelled regions is shown in Fig. S1.1.*

S2. The non-uniform twist distribution

The twirling device implemented in the AC electrospinning setup imparts to the emerging nanofibrous ribbon a so-called false twist. In textile engineering, a false-twist mechanism (in the case of this work it is the twirling device) temporarily rotates the ribbon during processing, but the imparted twist should theoretically relax once the mechanical constraint is removed, resulting in a final yarn with zero net twist. Accordingly, the AC electrospinning system used in this study is designed such that the produced nanofiber yarn should remain twist-neutral.

However, SEM micrographs of nanofiber yarns fabricated from PCL, PVA, and PVB consistently exhibit a distinct and unidirectional S-type twist (Fig. S2.1). No corresponding Z-type segments were detected along the examined yarn lengths. This is in contrast to the PA46 nanofiber yarn, in which no recognizable twist of any type is observed.

The unexpected presence of a systematic S-twist in the PCL, PVA, and PVB yarns suggests that a preferred handedness emerges during the consolidation of the nanofibrous ribbon into a yarn. A plausible contributing factor is the development of chiral instabilities during ribbon collapse, accompanied by van der Waals interactions that bond together individual fibres within the ribbon. Such chiral instabilities may preserve a transient rotational bias that survives the nominally twist-neutral conditions of the false-twist process. The origin of the emergent S-twist has not yet been fully elucidated and warrants deeper investigation, which will be addressed in future work.

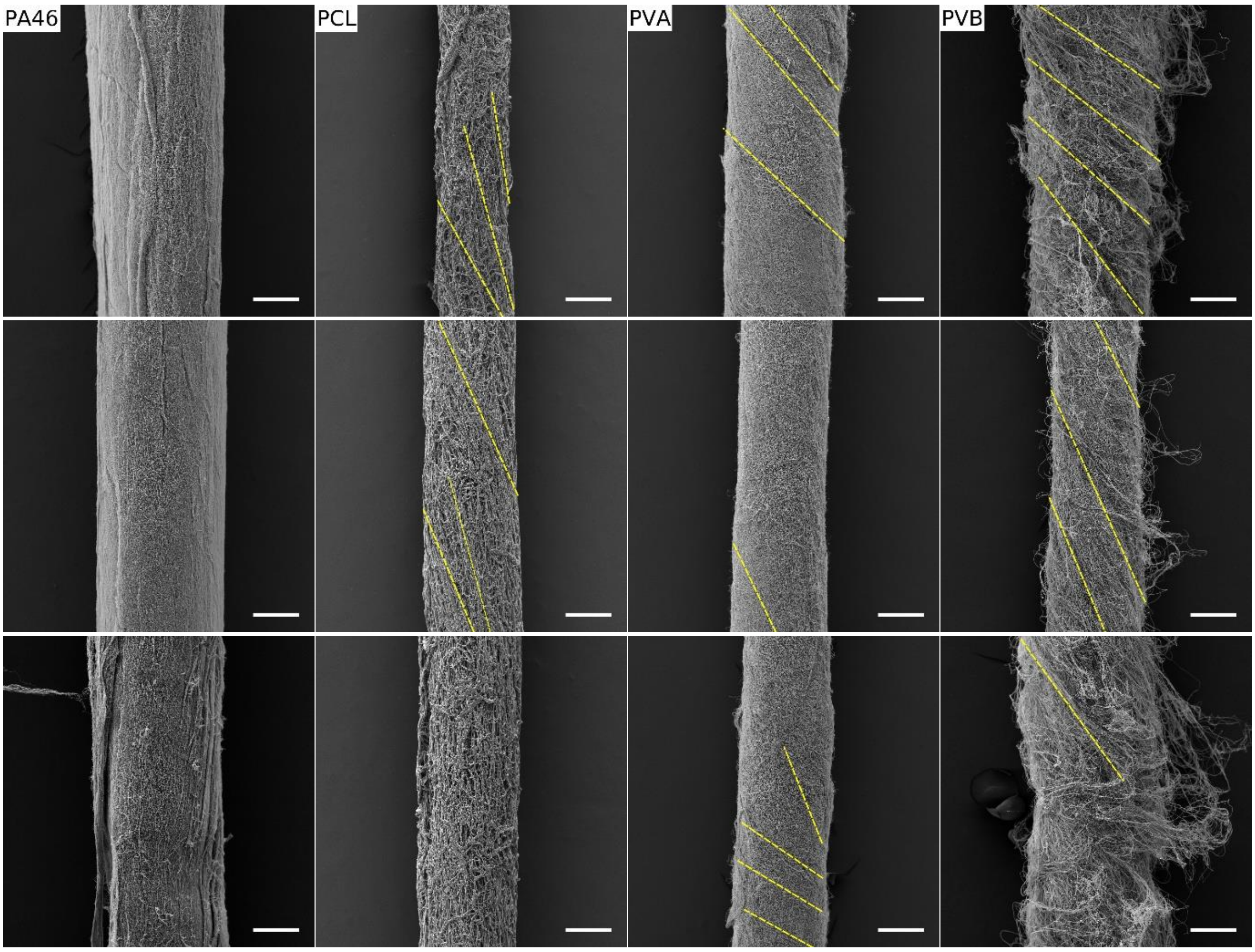

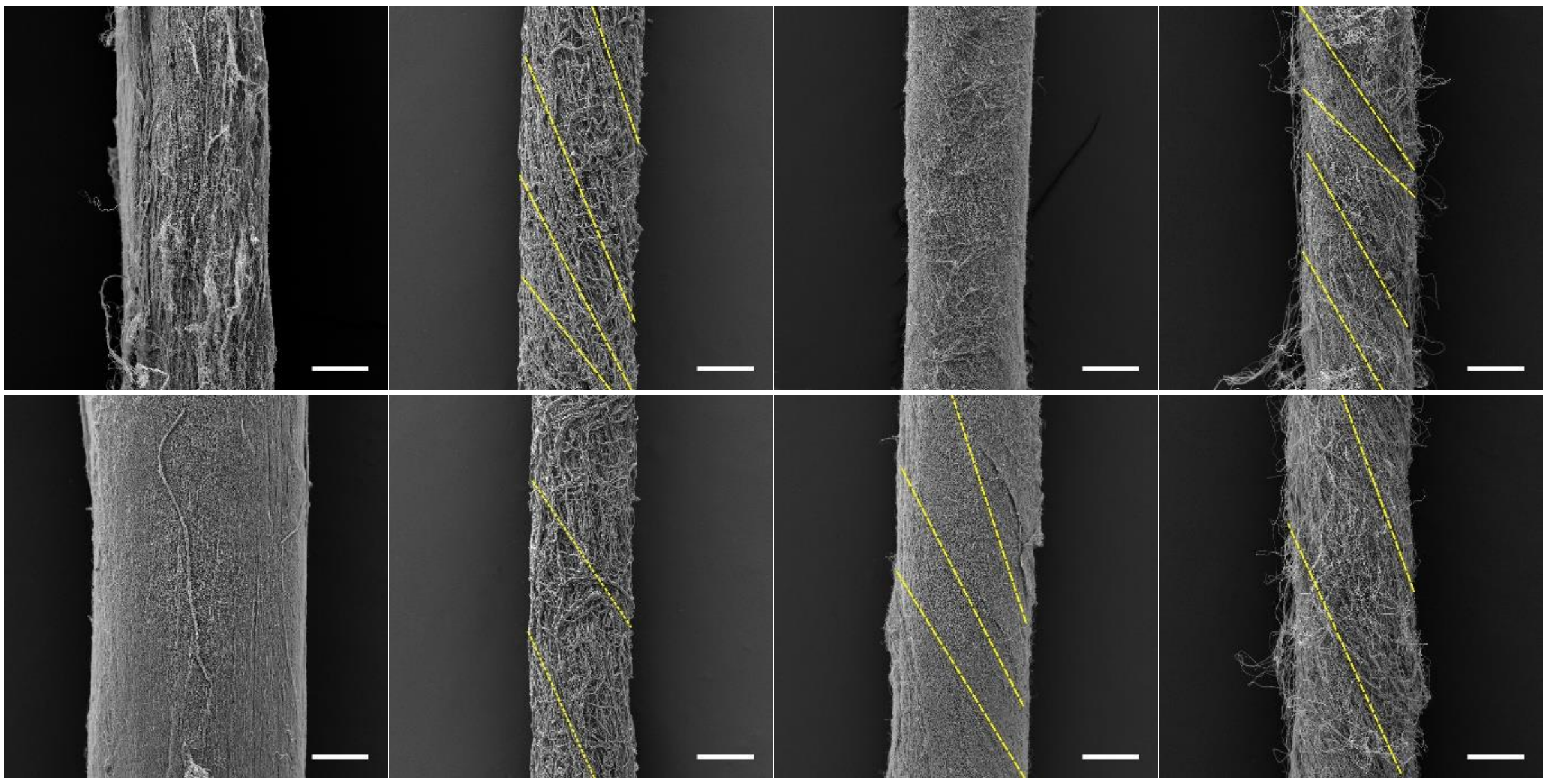

Fig. S2.1: Non-uniform twist distribution along the yarn axis. *SEM micrographs of the individual yarn samples. Columns represent identical material types to facilitate morphological comparison. A constant scale bar of 200 µm is applied throughout.*

S3 Stereological Analysis of Pore Size

To assess deviations from complete spatial randomness, i.e., Poisson point process, Monte Carlo simulation envelopes were constructed for the Ripley K-function and the corresponding $L(r)-r$ transformation. For each microphotograph, a series of simulated point patterns was generated under the assumption of complete spatial randomness, preserving both the number of points, i.e., the point process intensity, and the geometry of the observation window. For each simulated pattern, the same spatial statistics were calculated as for the experimental data. The resulting set of simulated curves was used to derive pointwise confidence envelopes, defined by the lower and upper quantiles corresponding to a 95% confidence level. The experimental curves were then compared to these envelopes to identify statistically significant deviations from spatial randomness. Values exceeding the upper envelope indicate spatial clustering, whereas values below the lower envelope indicate regularity or inhibition. For the overall analysis, envelope construction was performed on weighted averages of the simulated curves across all images, using weights proportional to the number of detected endpoints, ensuring consistency with the aggregation of the experimental data.

The stereological analysis performed for individual images yielded fibre number densities N_S in the range of 0.078–0.135 μm^{-2}. The corresponding most probable pore radii estimated from the Poisson-based model ranged from 1.08 to 1.43 µm, whereas the numerically derived values based on Monte Carlo simulations were consistently lower, spanning 0.96 to 1.32 µm.

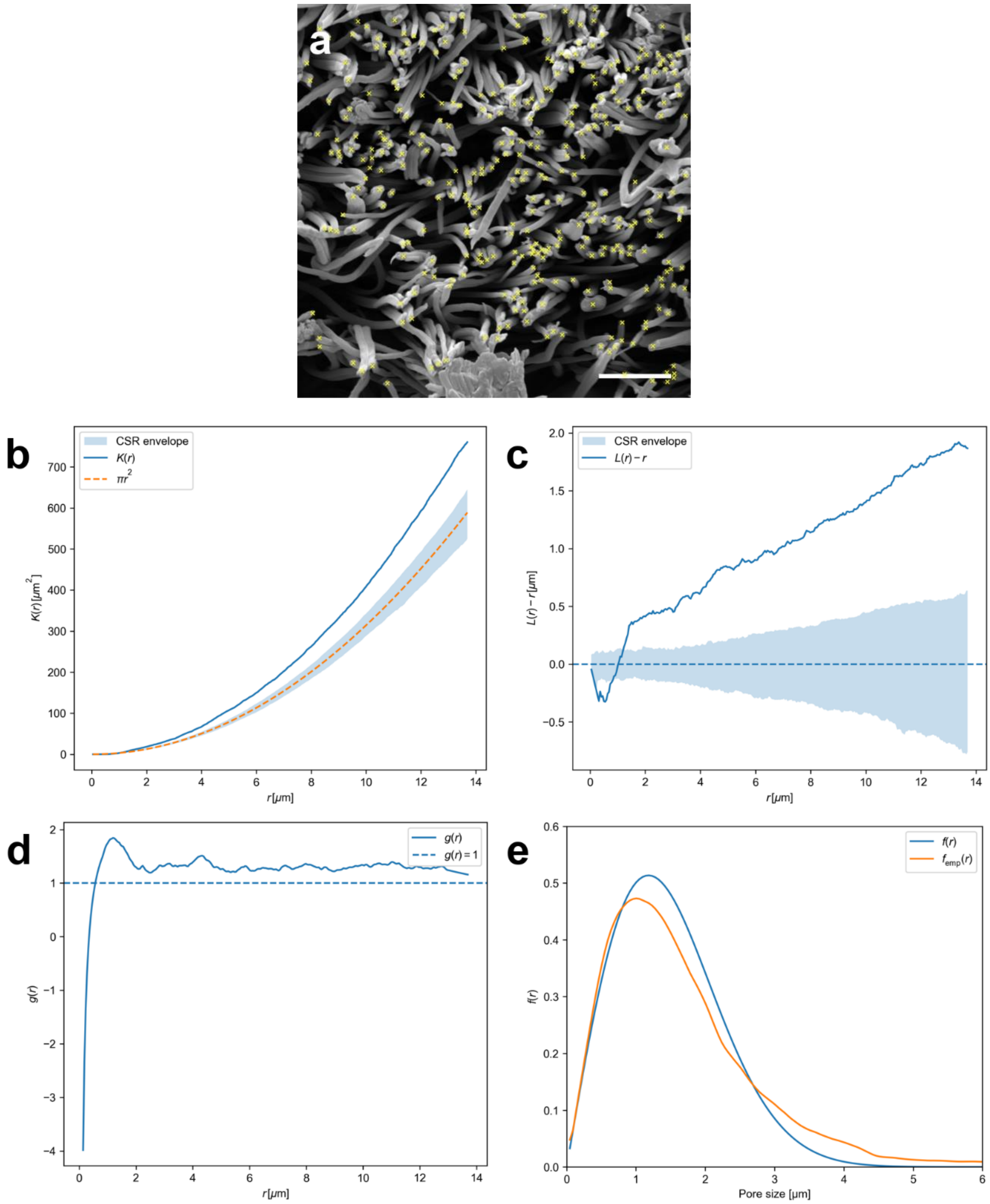

Fig. S3.1: Stereological analysis of the fibre endpoint point process. *(a) Representative micrograph with identified fibre endpoints highlighted. (b) K-function of the extracted point process. (c) L(r)−r function characterizing spatial deviations from Poisson point process. (d) Pair correlation function g(r). (e) Probability density function f(r) of the pore radius, indicating the most probable pore size of 1.18 µm.*

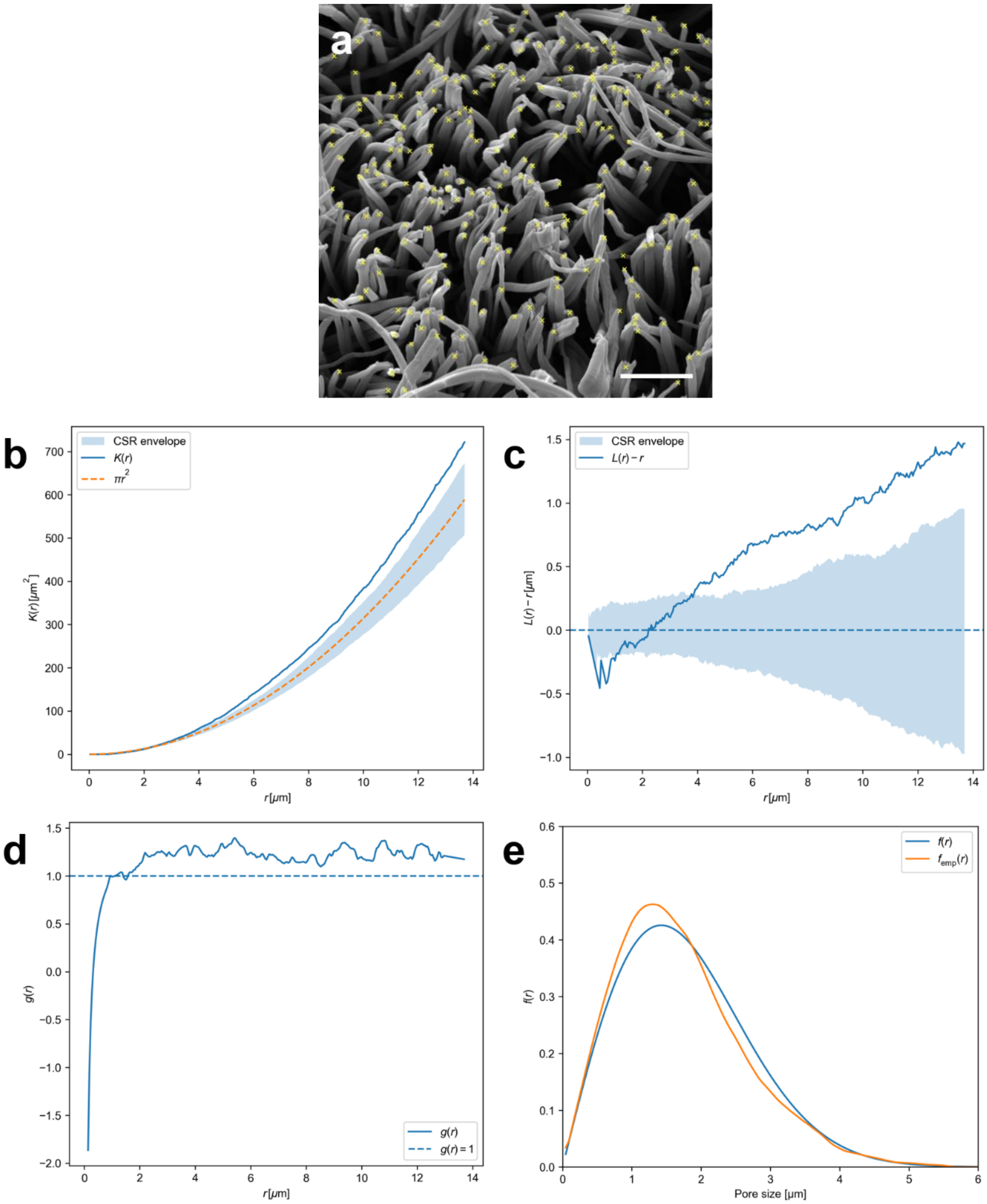

Fig. S3.2: Stereological analysis of the fibre endpoint point process. *(a) Representative micrograph with identified fibre endpoints highlighted. (b) K-function of the extracted point process. (c) L(r)−r function characterizing spatial deviations from* Poisson point process. *(d) Pair correlation function g(r). (e) Probability density function f(r) of the pore radius, indicating the most probable pore size of 1.42 µm.*

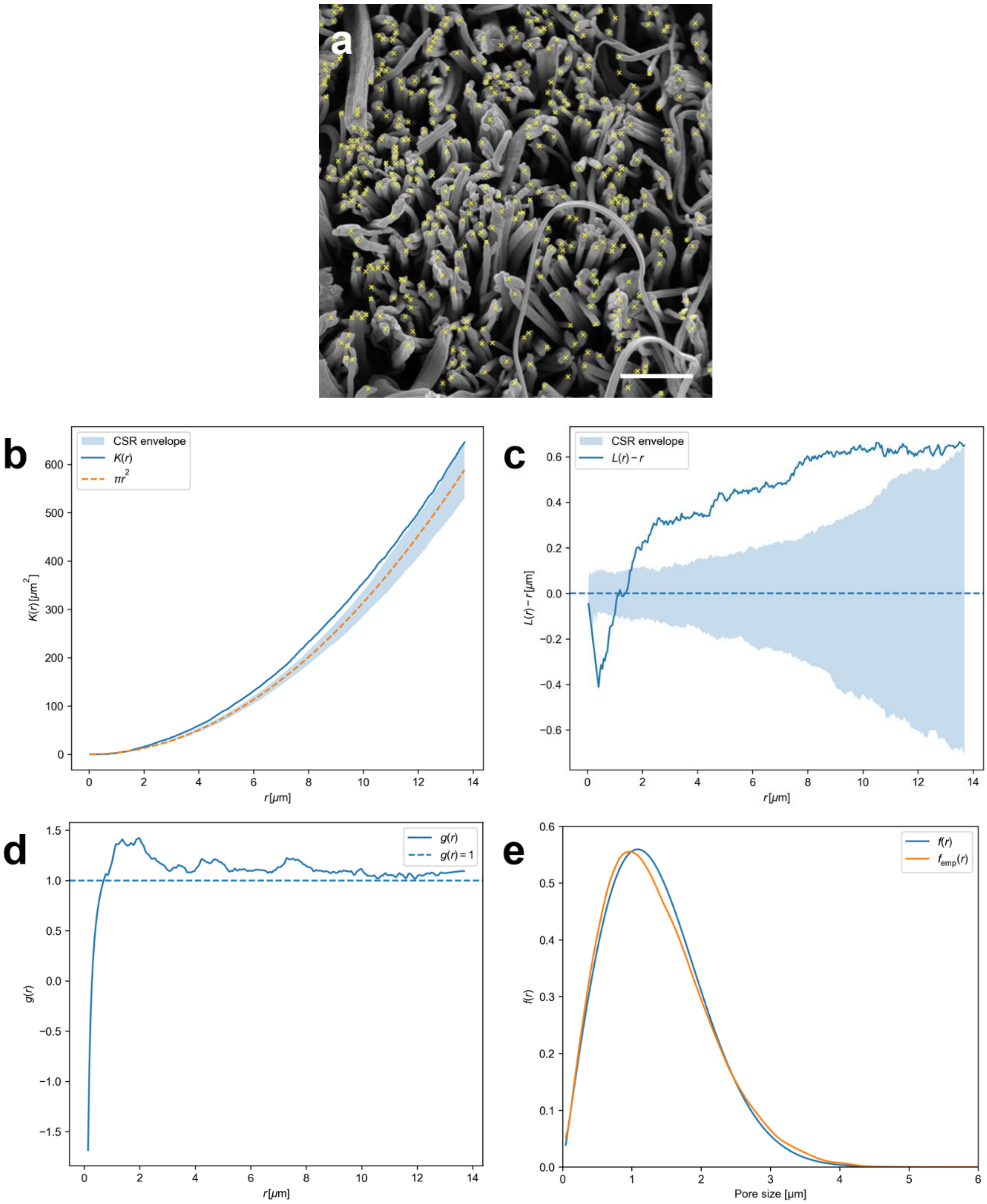

Fig. S3.3: Stereological analysis of the fibre endpoint point process. *(a) Representative micrograph with identified fibre endpoints highlighted. (b) K-function of the extracted point process. (c) L(r)−r function characterizing spatial deviations from* Poisson point process. *(d) Pair correlation function g(r). (e) Probability density function f(r) of the pore radius, indicating the most probable pore size of 1.08 µm.*

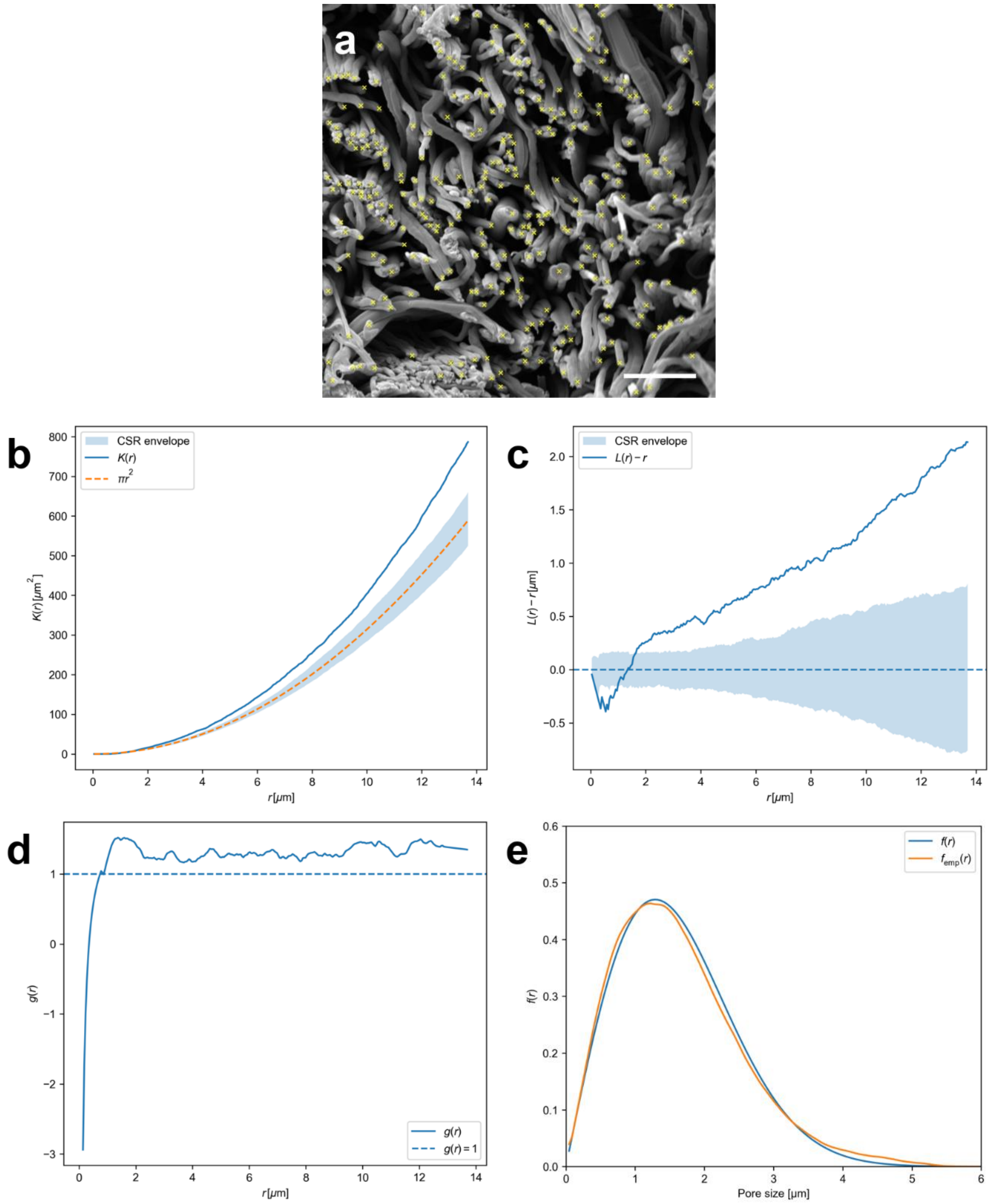

Fig. S3.4: Stereological analysis of the fibre endpoint point process. *(a) Representative micrograph with identified fibre endpoints highlighted. (b) K-function of the extracted point process. (c) L(r)−r function characterizing spatial deviations from Poisson point process. (d) Pair correlation function g(r). (e) Probability density function f(r) of the pore radius, indicating the most probable pore size of 1.28 µm.*

The global analysis based on the averaged data from all microphotographs yielded a fibre number density of $N_S = 0.106\ \mu m^{-2}$. The most probable pore radius obtained from the Poisson-based model was r =1.25 µm, while the value derived from Monte Carlo simulations was r = 1.13 µm. The close agreement between the two approaches, consistent with the trends observed

for individual images, indicates that the Poisson-based approximation provides a reasonable description of the pore size distribution despite local deviations in the point process.

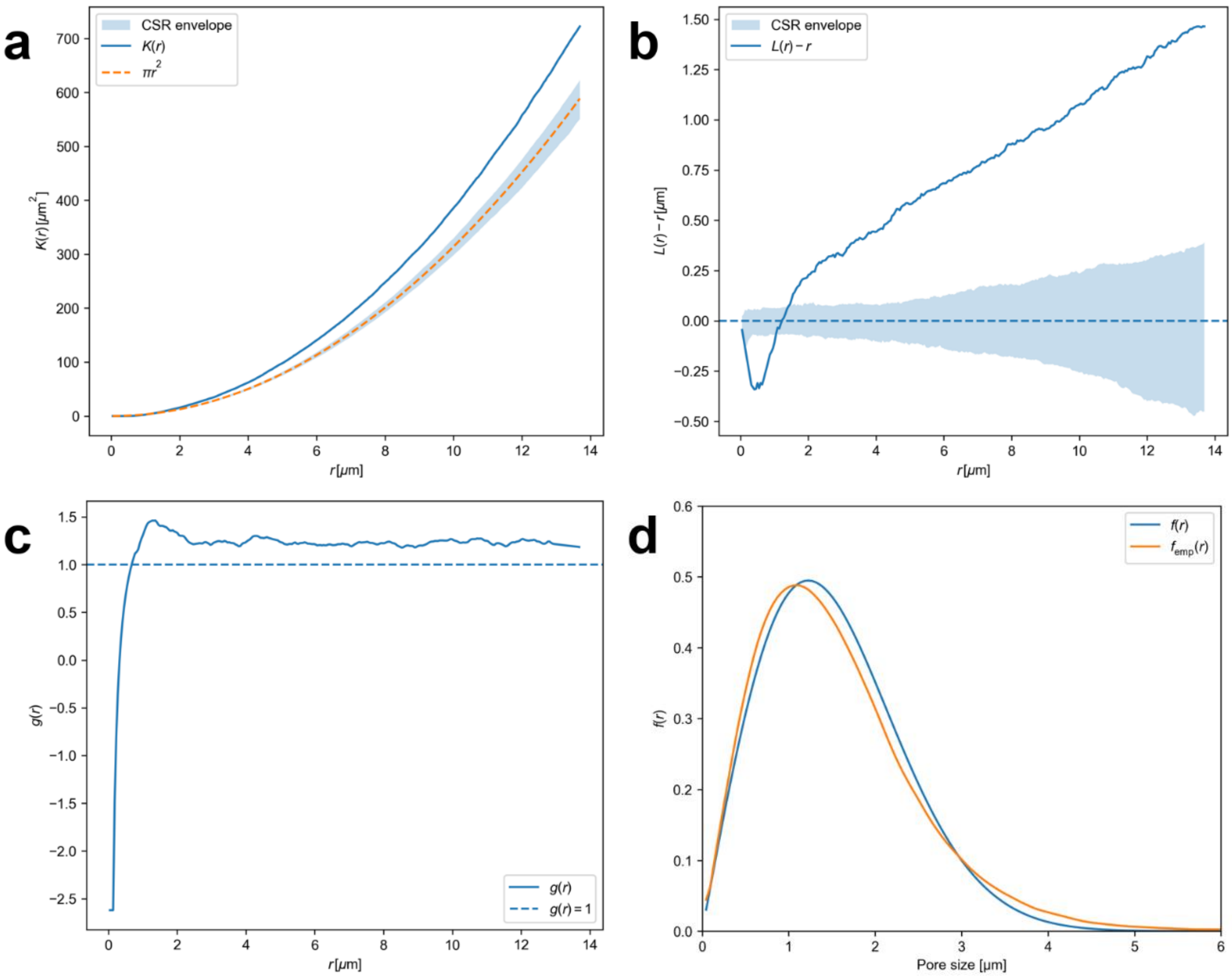

Fig. S3.5: Spatial analysis of the fibre-endpoint point process based on the weighted average of the individual image-level results, with the global point density $N_S = 0.106\ \mu m^{-2}$. *The Ripley K-function, L(r)−r function, and pair correlation function g(r) characterize deviations from complete spatial randomness. The pore-size density is shown as the Poisson-based analytical prediction f(r) (blue) and compared with the empirical Monte Carlo density derived from the experimental data (orange). The two approaches yield closely matching most probable pore radii*

S4 Measurement of the average curvature and torsion

This section summarises our approach to stereological estimation of fibre curvature and torsion on the surface of nanofibrous yarns. The definitions of curvature and torsion in fibrous systems are based on the classical concepts of the osculating plane, tangent, normal, and binormal vectors. Curvature and torsion are local geometrical characteristics that describe the spatial configuration of a linear structural element. To determine these parameters, we consider the neighbourhood of a point A located on a three-dimensional curve, as illustrated in Fig. S3.1.

The curvature, denoted as k, quantifies the rate of change of the tangent vector along the arc length in the vicinity of a point A, while the torsion, denoted as τ, represents the rate of rotation

of the osculating plane around the tangent. Averaged values of curvature and torsion provide global measures of bending and twisting within the fibrous system.

To define the curvature geometrically, two additional points B and C are placed on the same curve such that A lies between them. These three points uniquely determine a circle passing through B, A, and C. The limiting circle, obtained as B → A and C → A, is referred to as the osculating circle at point A. The plane containing this circle is called the osculating plane. Within this plane lies the normal vector ***n***, which is a unit vector originating at point A and directed toward the centre S of the osculating circle.

The unit vector lying in the osculating plane, originating at point A and perpendicular to the normal vector, is called the tangent vector ***t*** to the curve at point A. Together, the tangent (***t***) and normal (***n***) vectors uniquely define a third vector, the binormal vector ***b***, which is orthogonal to both. The three mutually perpendicular unit vectors — ***t***, ***n***, and ***b*** — constitute an orthonormal right-handed basis in three-dimensional space. This local coordinate system, known as the Frenet–Serret frame, fully describes the instantaneous orientation and spatial behaviour of the curve (or fibre axis) at point A.

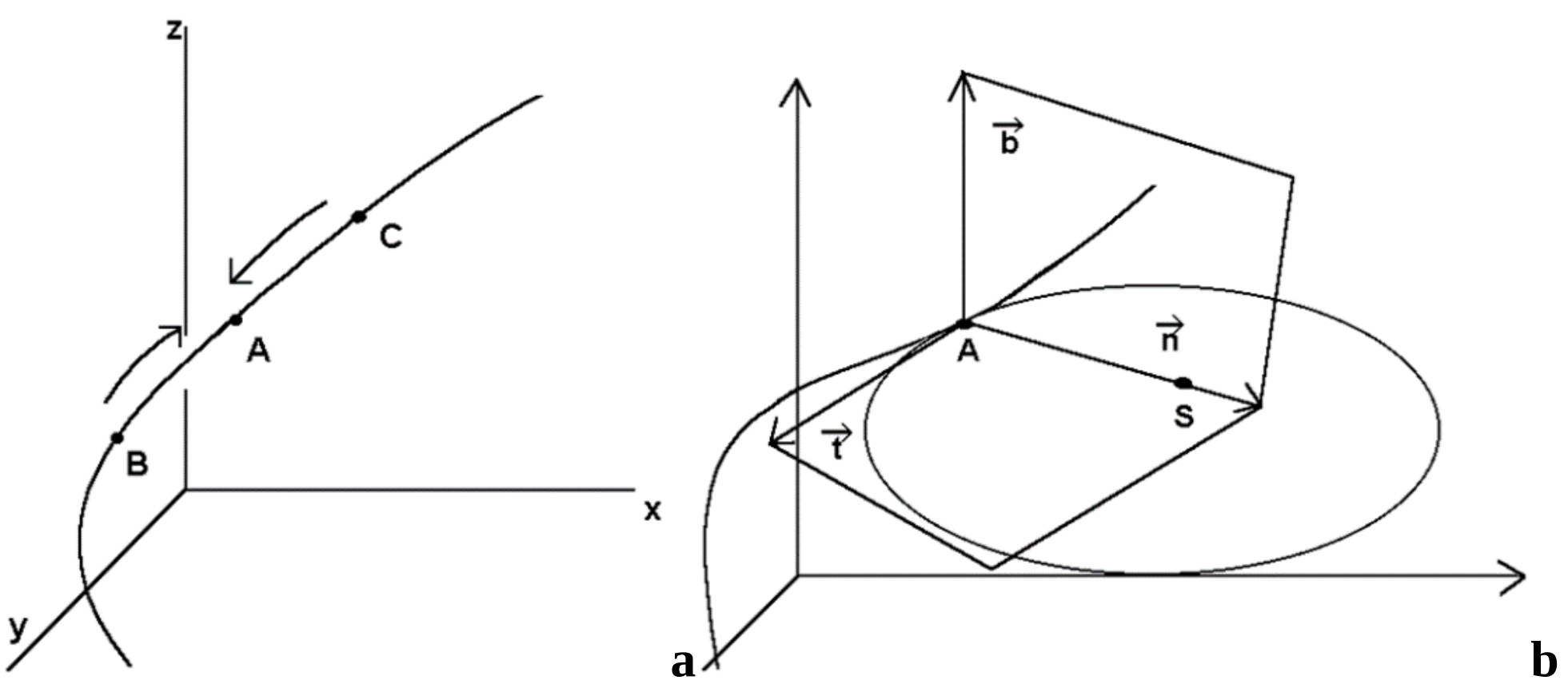

Fig. S4.1: Determination of curvature and torsion of a linear structural element. *(a) In the vicinity of point A, two neighbouring points B and C are located on the same curve such that A lies between them. (b) The osculating circle to the curve at point A defines the local curvature and the corresponding osculating plane.*

When point A is displaced along the curve by an infinitesimal arc length dλ, the orientations of all three vectors — ***t***, ***n***, and ***b*** — change accordingly. The new vectors generally form non-zero angles with their original positions. The angle between the original and displaced tangent vectors is denoted as dθ, while the angle between the original and displaced binormals is denoted as dγ.

The curvature k at point A is defined as the rate of change of the tangent vector's direction with respect to the arc length λ

$$k = \frac{d\theta}{d\lambda}. \qquad \text{(S4.1)}$$

Similarly, the torsion τ is defined as the rate of change of the binormal vector's direction with respect to the same arc length

$$\tau = \frac{d\gamma}{d\lambda}. \qquad \text{(S4.2)}$$

From these definitions, it follows that curvature is associated with changes in the orientation of the tangent vector, while torsion reflects changes in the orientation of the binormal vector along the curve.

The average curvature $\bar{k}$ is defined as the mean value of the local curvature k along a curve segment of length L

$$\bar{k} = \frac{1}{L}\int_0^L k(\lambda)\, d\lambda. \qquad \text{(S4.3)}$$

Similarly, the average torsion $\bar{\tau}$ is given by the mean value of the local torsion τ along the same segment:

$$\bar{\tau} = \frac{1}{L}\int_0^L \tau(\lambda)\, d\lambda. \qquad \text{(S4.4)}$$

The average curvature $\bar{k}$ and torsion $\bar{\tau}$ provide global quantitative descriptors of the bending and twisting behaviour of the fibrous element, respectively. The method used to determine these average values is illustrated schematically in Fig. S4.2.

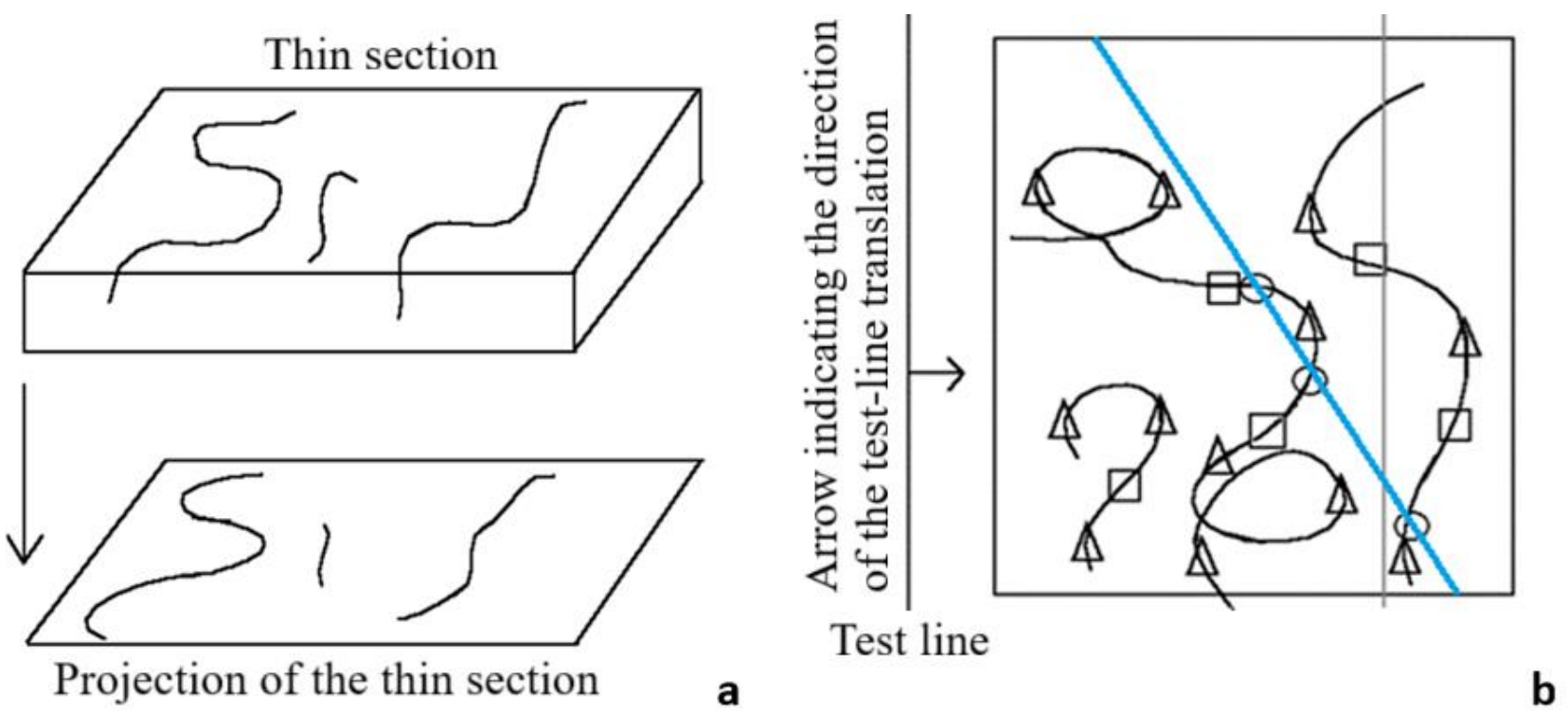

Fig. S4.2: Experimental determination of average curvature and torsion. *(a) Projection of a thin section containing linear structural elements (curves, fibres). (b) Tangential positions of the translating test line relative to the curves are marked with triangles (△). Inflection points are indicated by squares (□). Intersections with the test line (blue line) are marked with circles (○).*

First, we focus on the mean torsion. It is estimated using relation (S4.5), which we state here without proof, more information are available in De Hoff (1975).

$$[\bar{\tau}] = \frac{\pi}{2}\frac{I_A}{N_L}, \qquad \text{(S4.5)}$$

where, I_A is the number of inflection points per unit area of the projection. The quantity N_L denotes the number of intersections between a uniformly random and isotropic test line and the curve / fibrous system projection per unit length of this test line.

The mean curvature is estimated using the following relation, again after De Hoff (1975).

$$[\bar{k}] = \frac{\pi}{2}\frac{T_A}{N_L}. \tag{S4.6}$$

Here, T_A denotes the number of tangent positions of the test line per unit area of the projection. To determine these tangent positions, the test line is translated across the projection while maintaining its original orientation in all positions. The estimate is refined by averaging over an isotropically random set of orientations of the original test-line direction.

Experimental determination of curvature and torsion of nanofibrous yarns

The pronounced curvature and torsion of nanofibers assembled on the surface of nanofibrous yarns clearly distinguish these structures from classical yarns, which consist of orders-of-magnitude fewer elementary fibrils with substantially larger diameters. For the quantitative determination of the average curvature and torsion, a nanofibrous PA 4,6 yarn was selected. The SEM micrographs used for this morphological analysis are shown in Fig. S4.3. The images in the left-hand column, Fig. S4.3(a, c, e, g, i), represent magnified views of regions displayed in Fig. S4.3(b, d, f, h, j). These regions are positioned near the central axis of the yarn to minimise any artefacts arising from surface rounding toward the yarn edges.

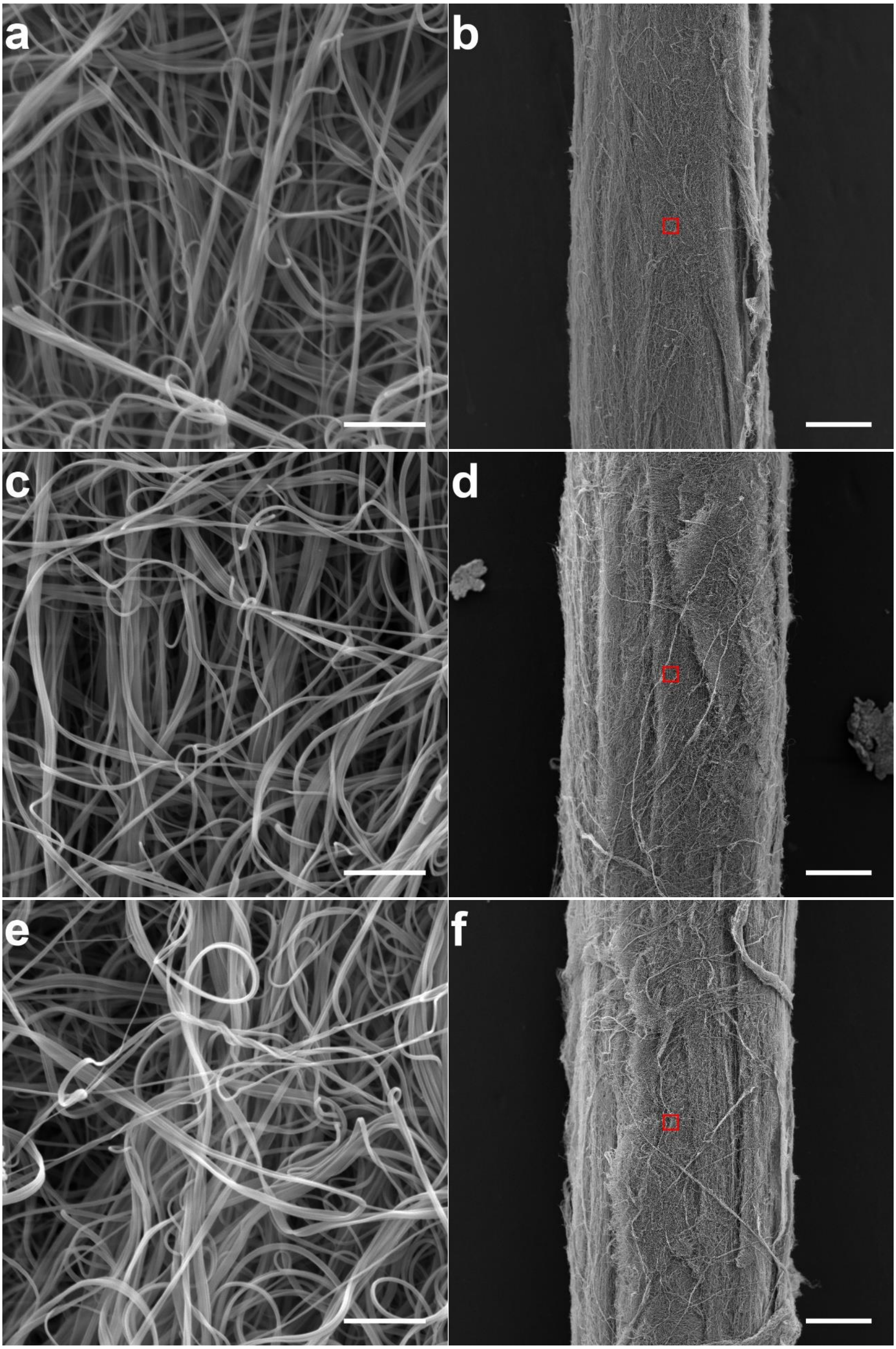
a
b
c
d
e
f

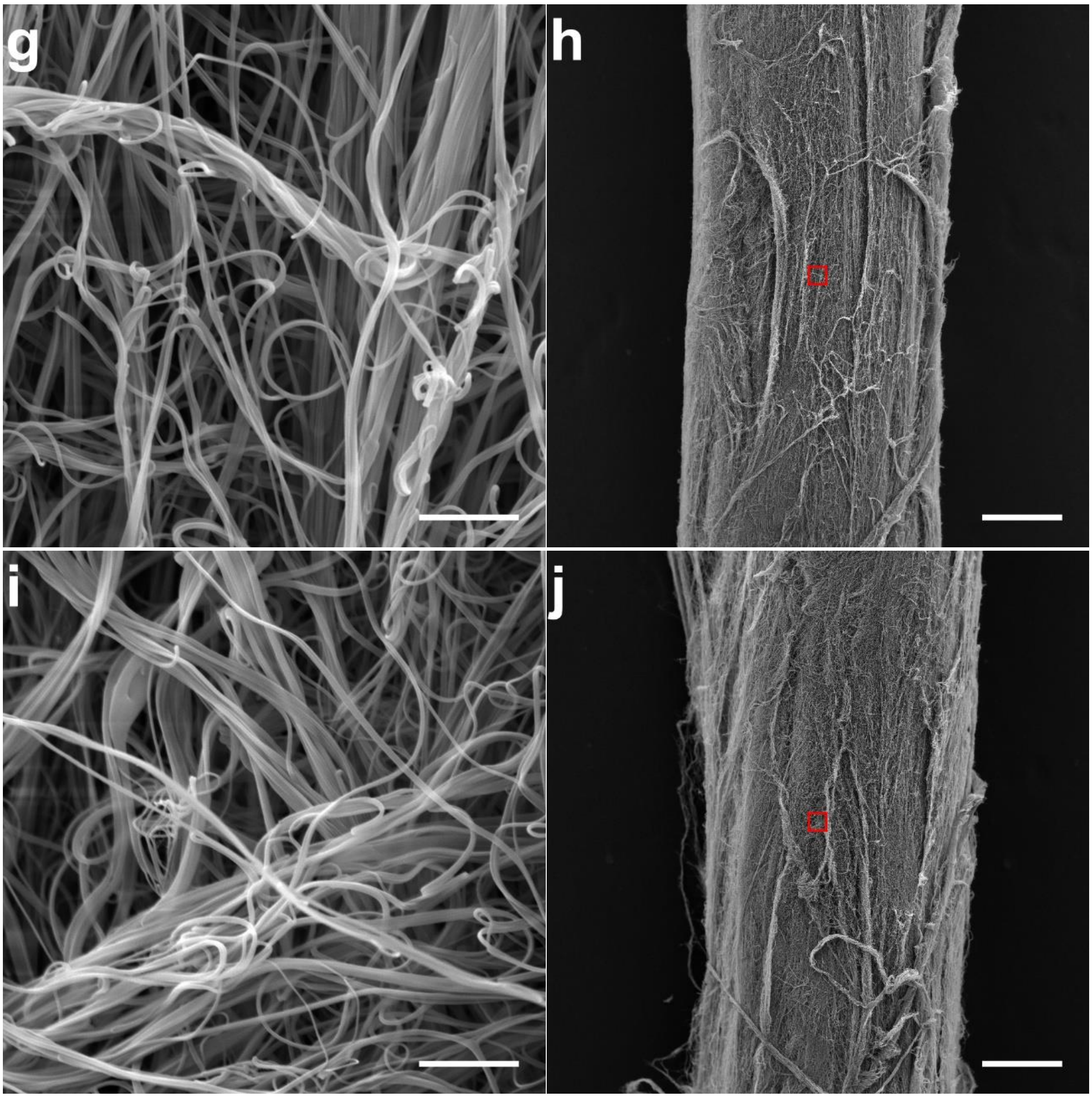

Figure S4.3: SEM microphotographs of PA4,6 nanoyarn for stereological measurements. *The images in the left-hand column (scale bar: 5 μm) represent magnified views of regions displayed in the right-hand column (scale bar: 200 μm). These regions are positioned near the central axis of the yarn to minimise any artefacts arising from surface rounding toward the yarn edges.*

Table S4.1: *Experimentally determined values of* N_L *(number of fibre–test line intersections per unit test-line length),* T_A *(number of tangential contacts of the sliding test line with the fibrous system per unit area of the reference region), and the resulting estimates of the mean curvature* $[\bar{k}]$.

Measurement ID	$T_A\ [\mu m^{-2}]$	$N_L [\mu m^{-1}]$	$[\bar{k}] = \frac{\pi}{2}\frac{T_A}{N_L} [\mu m^{-1}]$
1	0.0844	1.467	0.0844
2	0.0867	1.903	0.0716
3	0.1131	1.559	0.1139
4	0.1209	1.831	0.1038

5	0.1078	1.649	0.1064
Mean	0.1026	1.682	0.0960
Standard deviation	0.0163≅0.02	0.183≅0.2	0.0175≅0.02

The experimentally determined values of N_L (number of intersections between the fibrous system and the test line per unit test-line length), T_A (number of tangential contacts between the sliding test line and the fibrous system per unit area of the reference region), and the resulting estimates of the mean curvature $[\bar{k}]$ are summarized in Table S4.1.

For clarity, Table S4.2 again lists the experimentally determined values of N_L, together with I_A (number of inflection points per unit area of the reference region), and the corresponding estimates of the mean torsion $[\bar{\tau}]$.

Table S4.2: *Experimentally determined values of* N_L *(number of intersections between the fibrous system and the test line per unit test-line length),* I_A *(number of inflection points per unit area of the reference region), and the resulting estimates of the mean torsion* $[\bar{\tau}]$.

Measurement ID	$I_A\ [\mu m^{-2}]$	$N_L [\mu m^{-1}]$	$[\bar{\tau}] = \frac{\pi}{2}\frac{I_A}{N_L} [\mu m^{-1}]$
1	0.0342	1.467	0.0366
2	0.0355	1.903	0.0293
3	0.0302	1.559	0.0302
4	0.0381	1.831	0.0327
5	0.0342	1.649	0.0325
Mean	0.0344	1.682	0.0323
Standard deviation	0.0029≅0.003	0.183≅0.2	0.0028≅0.003

(i) The experimentally obtained mean curvature of $\bar{k}$ = 0.096 $\mu m^{-1} \pm$ 0.02 μm^{-1} corresponds to the curvature of a circle with radius $a = 1/0.096 = 10.4\ \mu m$.

(ii) The experimentally determined mean torsion of the surface fibres, $\bar{\tau}$ = 0.0323 $\mu m^{-1} \pm$ 0.003 μm^{-1} together with mean curvature can be related to an ideal helical geometry. A perfect circular helix (i.e., one with constant radius) exhibits constant curvature k and constant torsion τ. For such a helix, the curvature and torsion are given by:

$$k = \frac{a}{a^2+b^2}, \qquad \tau = \frac{b}{a^2+b^2}, \tag{S4.7}$$

where a is the helix radius and $2\pi b$ is the pitch parameter. The pitch of a helix is the height of one complete turn of the helix, measured parallel to the helix axis. These relations allow the measured pair ($\bar{k}$,$\bar{\tau}$) to be interpreted in terms of an equivalent helical geometry of the surface fibres.

$$\bar{k} = 0{,}096\ \mu m^{-1}, \qquad \bar{\tau} = 0{,}0323\ \mu m^{-1}, \tag{S4.8}$$

Using the experimentally determined values of $\bar{k}$ and $\bar{\tau}$, we compute the parameters a and b of the equivalent ideal helix. Starting from Eqs. (S4.7) and, we first isolate the common term $a^2 + b^2$ from both expressions for curvature and torsion:

$$a^2 + b^2 = \frac{a}{k}, \qquad\qquad a^2 + b^2 = \frac{b}{\tau}$$

It follows immediately that

$$a = \frac{k}{\tau} b. \tag{S4.9}$$

We substitute this relation for *a* into the torsion expression in Eq. (S4.7) and simplify:

$$\tau = \frac{b}{a^2+b^2} = \frac{b}{\left(\frac{k}{\tau} b\right)^2 + b^2} = \frac{\tau^2}{b(k^2+\tau^2)}. \tag{S4.10}$$

From this relation we directly obtain the helix parameter *b*:

$$b = \frac{\tau}{k^2+\tau^2}. \tag{S4.11a}$$

Substituting this value of *b* back into Eq. (S4.10) yields the corresponding expression for *a*:

$$a = \frac{k}{k^2+\tau^2}. \tag{S4.11b}$$

The corrugation (“crimping”) of the surface filaments can therefore be approximated by helical trajectories with a radius

$$a = \frac{k}{k^2+\tau^2} = \frac{0{,}096}{0{,}096^{\,2}+0{,}0323^2}\,\mu\text{m} = \frac{0{,}096}{0{,}0103}\,\mu\text{m} = 9{,}32\mu\text{m}.$$

Similarly, the pitch of the helix is given by

$$2\pi b = 2\pi \frac{0{,}0323}{0{,}096^{\,2}+0{,}0323^2}\,\mu\text{m} = 2\pi \frac{0{,}0323}{0{,}0103}\,\mu\text{m} = 19{,}70\mu\text{m}.$$
