# Supplementary material for: High-throughput fabrication of nanofibrous yarns produced by alternating-current electrospinning
Source: Commun Eng. 2026 Jul 22;5:196. doi: 10.1038/s44172-026-00737-x (PMC13759490; doi:10.1038/s44172-026-00737-x)
Supplement: Supplementary file 3 [file 44172_2026_737_MOESM3_ESM.pdf]

Supplementary Movie 1 | Jet and fibre formation at the edge of a rotating disc-shaped spinning electrode. High-speed-camera recording showing multiple polymer jets emerging from the edge of the rotating disc-shaped spinning-electrode during alternating-current electrospinning. The jets originate from electrohydrodynamic instabilities on the liquid surface and subsequently form nanofibres.

Supplementary Movie 2 | Nanofibrous yarn formation by alternating-current electrospinning. Video recording showing nanofibres generated from the rotating disc-shaped spinning-electrode being deposited on a drum collector to form a nanofibrous belt, which is then continuously twisted into a yarn using a twirling device.

Supplementary Movie 3 | Spinning triangle during nanofibrous yarn formation. Video recording showing the spinning triangle formed as the nanofibrous belt is drawn from the drum collector and twisted into a continuous nanofibrous yarn. Yarn ballooning can be observed between the twirling device and the tip of the spinning triangle.

Supplementary Movie 4 | Galette-assisted stabilization of nanofibrous yarn formation. Video recording showing the galette positioned downstream of the twisting zone during nanofibrous yarn formation. The galette acts as a tension stabilizer and twist brake, defining a downstream boundary condition for the twisting process. A mechanical tension sensor placed in front of the galette continuously monitors the yarn tension.
